# Bio‐Based Amphiphilic Farnesyl Glycidyl Ether Block Copolymers: Aqueous Self‐Assembly and Solubilization Boosting

**DOI:** 10.1002/chem.202502614

**Published:** 2025-11-17

**Authors:** Maximilian Krappel, Sandra Schüttner, Ingo Schneider, Pascal Schiffmann, Ralf Schweins, Holger Frey, Thomas Sottmann

**Affiliations:** ^1^ Institute of Physical Chemistry University of Stuttgart Pfaffenwaldring 55 70569 Stuttgart Germany; ^2^ Department of Chemistry Johannes Gutenberg University Mainz Duesbergweg 10‐14 55128 Mainz Germany; ^3^ DS / LSS Institut Laue‐Langevin 71 Avenue des Martyrs, CS 20 156 Grenoble CEDEX 9 38042 France

**Keywords:** amphiphilic block copolymers, biobased glycidyl ethers, efficiency boosting, self‐assembly, small‐angle neutron scattering

## Abstract

Sustainability has become essential in addressing the substitution of depleting fossil‐based resources with bio‐renewable alternatives, including products active at interfaces, such as surfactants. Enhancing their efficiency reduces both ecological and economic impact. Here, we present amphiphilic poly(ethylene glycol)‐*b*‐poly(terpenyl glycidyl ether) diblock copolymers synthesized via anionic ring‐opening polymerization from two terpenyl glycidyl ethers (TGE) based on the naturally occurring terpenoid farnesol, farnesyl glycidyl ether (FarGE), and its hydrogenated derivative hexahydrofarnesyl glycidyl ether (HHFarGE). Using poly(ethylene glycol) monomethyl ether (mPEG_114_) as a macroinitiator resulted in controlled molecular weights (5600 – 8400 g·mol^−1^) with low dispersities *Đ* (1.04–1.07). Fluorescence spectroscopy and light scattering revealed low critical micelle concentrations with a systematic decrease with increasing TGE block size due to the hydrophobic effect. The addition of small amounts of mPEG_114_‐*b*‐PTGE_m_ to microemulsions leads to a significant increase of the solubilization efficiency not limited to conventional H_2_O/NaCl – *n*‐decane – tetraethylene glycol monodecyl ether microemulsions, but also in sustainable H_2_O – isopropyl myristate – *n*‐octyl β‐D‐glucopyranoside – farnesol formulations, serving as model systems for cosmetic applications. Using SANS, we observed that adsorption of the copolymer at the amphiphilic film leads to an increased structural order of bicontinuous microemulsions due to a higher film bending rigidity.

## Graphical abstract

Bio‐derived amphiphilic poly(ethylene glycol)‐b‐poly(terpenyl glycidyl ether) diblock copolymers (mPEG114‐b‐PTGEm) were synthesized via AROP from farnesyl glycidyl ether (FarGE) and its hexahydrogenated derivative (HHFarGE). Block copolymer amphiphilicity is easily tunable via the TGE block size m and systematically affects the critical micelle concentration. Adding even small amounts of mPEG114‐b‐PTGEm to microemulsions leads to a significant increase of the solubilization efficiency.

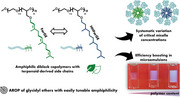

## 1 Introduction

Current predominant dependence on rapidly depleting fossil fuel resources and the awareness of our environmental footprint reset the focus toward a more sustainable design and application of polymeric materials.^[^ ^1^ ^]^ Synthetic strategies involve the substitution of fossil‐based monomers with those from bio‐renewable feedstocks.^[^ ^2^, ^3^ ^]^ In this context, terpenes and their functional derivatives, so‐called terpenoids, have emerged as highly versatile polymer building blocks.^[^ ^1^, ^2^, ^4^, ^5^, ^6^ ^]^ Their structural and functional variety enables the design and synthesis of polymers that are advanced materials, providing a vast field of polymer applications ranging from pressure‐sensitive adhesives to resins and thermoplastic elastomers.^[^ ^2^, ^7^, ^8^, ^9^, ^10^ ^]^ Not only are terpenes and terpenoids naturally abundant compounds in essential plant oils and fruits, but technological progress for microbial terpene production via biosynthesis is ongoing, thus increasing the availability and value of these building blocks.^[^ ^5^, ^11^, ^12^ ^]^

**Scheme 1:**
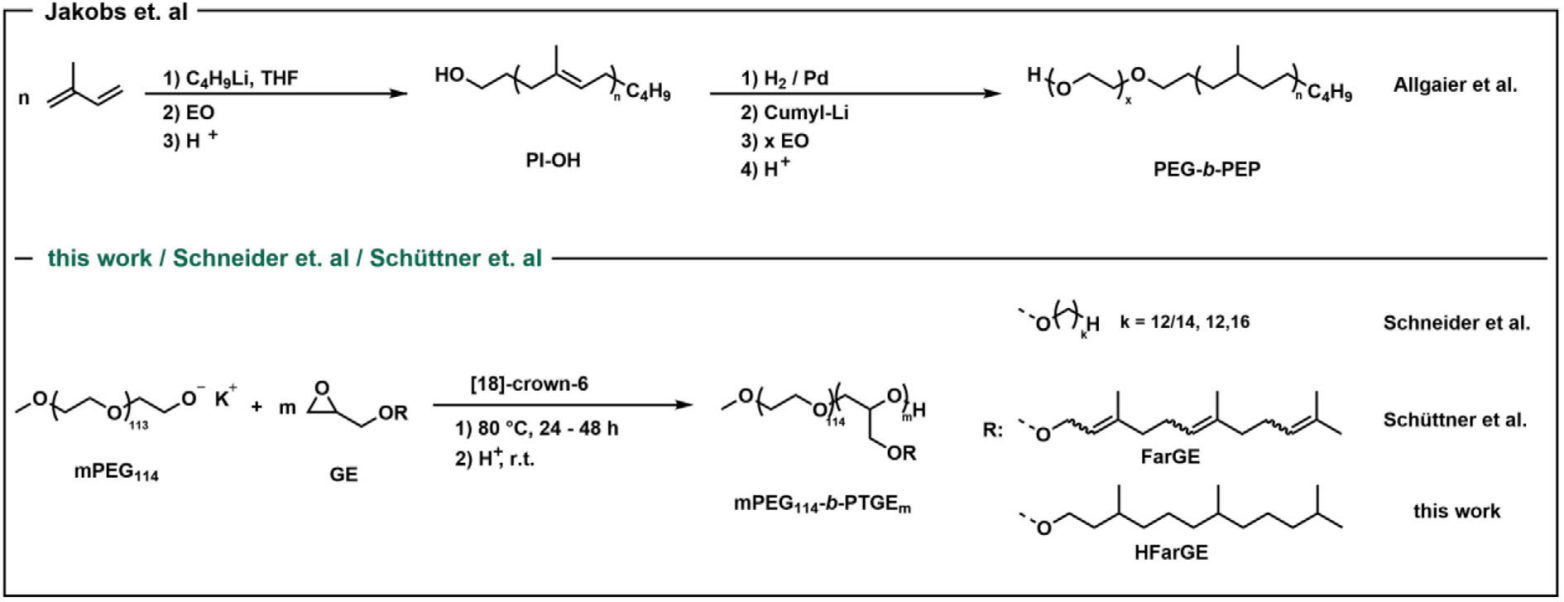
Comparison of synthetic strategies of various PEG‐based diblock copolymers, which have been investigated as cosurfactants in microemulsions.^[^ ^15^, ^31^, ^32^, ^33^ ^].^

Two acyclic terpenoids of interest, namely farnesol (3,7,11‐trimethyl‐2,6,10‐dodecatrien‐1‐ol) and its hydrogenated analogue hexahydrofarnesol (3,7,11‐trimethyl‐dodecan‐1‐ol, HHF), are characterized by their hydrophobic, branched C_15_ chain.^[^ ^5^ ^]^ Industrially, farnesol can be sourced from the isomerization of the more abundant *trans*‐nerolidol, whereas HHF is a byproduct of the microbial farnesane production, a sustainable aviation fuel.^[^ ^5^, ^12^, ^13^ ^]^ The hydrophobic nature renders the terpene‐alcohols suitable for the synthesis of monomer building blocks for polymeric amphiphiles or a surfactant itself.^[^ ^14^ ^]^ Recently, we introduced farnesyl glycidyl ether for the synthesis of amphiphilic copolyethers with a farnesyl side chain.^[^ ^15^ ^]^ They represent a novel group of amphiphilic block copolymers, whose bio‐based nature and a simplified synthesis route (Scheme 1) could open new avenues of applications, for instance in cosmetics.^[^ ^15^ ^]^ Note that Aubry and coworkers reported on the coupling of HHF with mono‐ and disaccharides, which allowed for the synthesis of a fully bio‐based sugar surfactant from an industrial byproduct.^[^ ^16^ ^]^

Surfactants enable the mutual solubilization of otherwise immiscible polar (e.g., water) and nonpolar (e.g., oil) components into each other. While ordinary emulsions are thermodynamically unstable, microemulsions are thermodynamically stable. The latter are facilitated by the surfactants’ significant reduction of the interfacial tension between water and oil, which reduces the free energy, rendering the thermal energy sufficient for the formation of a surfactant monolayer separating water and oil nanodomains.^[^ ^17^, ^18^, ^19^ ^]^ Ever since Hoar and Schulman's pioneering work in the 1940s,^[^ ^20^, ^21^ ^]^ microemulsions have gained significant research interest, which has elucidated the influence of composition, temperature, and pressure on microemulsion phase behavior and nanostructure.^[^ ^18^, ^19^, ^22^, ^23^, ^24^, ^25^, ^26^ ^]^ Combining the advantages of the achieved miscibility of typically immiscible components, the strong reduction of the interfacial tension, and the adjustable microstructures with different properties,^[^ ^18^, ^19^, ^27^ ^]^ microemulsions have become indispensable for a wide range of household, biomedical, and industrial applications.^[^ ^28^, ^29^, ^30^ ^]^

However, a major disadvantage of microemulsions compared to conventional emulsions is the significantly higher amount of surfactants that must be utilized for their formulation. One successful approach to address this disadvantage was the observation made 25 years ago by Jakobs et al. They found that the solubilization efficiency of a nonionic tetraethylene glycol monodecyl ether (C_10_E_4_) surfactant in a ternary H_2_O – *n*‐decane – C_10_E_4_ microemulsion can be significantly increased by adding small amounts of amphiphilic block copolymers of the type poly(ethylene glycol)‐*b*‐poly(propylene) (PEG_n_‐*b*‐PEP_m_), which became known as the “efficiency boosting effect”, established a way to significantly reduce the amount of surfactant required to formulate microemulsions.^[^ ^33^ ^]^

This striking effect could be assigned to the adsorption of the PEG_n_‐*b*‐PEP_m_ copolymers into the interfacial film, proven by double contrast variation small‐angle neutron scattering (SANS) studies,^[^ ^34^, ^35^, ^36^, ^37^ ^]^ which influences the properties of the amphiphilic film, that is, particularly the elastic moduli, bending rigidity κ and saddle splay modulus $\bar{\kappa }$. Accordingly, the efficiency boosting effect of the PEG_n_‐*b*‐PEP_m_ copolymers could be scaled against the polymer coverage of the amphiphilic film.^[^ ^34^, ^35^, ^36^, ^37^, ^38^, ^39^, ^40^ ^]^

However, while the PEG_n_‐*b*‐PEP_m_ copolymers studied by Jakobs et al. showed a strong efficiency boosting not only in microemulsions stabilized by nonionic but also by ionic surfactants, a complex copolymer synthesis prevented utilization at least on a larger scale (cf. Scheme 1).^[^ ^33^ ^]^ Since then, multiple works have explored a variety of amphiphilic polymer structures to investigate their respective efficiency boosting effect in microemulsions. Examples include PEG‐based di‐ and triblock copolymers with varying hydrophobic blocks as well as comb‐like, Y‐shaped, and random copolymers.^[^ ^32^, ^41^, ^42^, ^43^, ^44^, ^45^, ^46^, ^47^ ^]^ In one of the more recent works, Schneider et al. studied the efficiency boosting of fully polyether‐based amphiphilic copolymers, produced in a simplified synthetic approach applying long‐chain alkyl glycidyl ethers (AlkGE) as hydrophobic block.^[^ ^32^ ^]^ By performing a systematic phase behavior and SANS study, they could prove a similar scaling for the poly(ethylene glycol)‐*b*‐poly(alkyl glycidyl ether) (mPEG_113_‐*b*‐PAlkGE_m_) copolymers if the same oil (*n*‐decane) is used, whereas another oil (*n*‐octacosane) showed a different factor, which the authors attributed to polymers at higher temperatures behaving more like ideal chains.^[^ ^32^, ^48^ ^]^

Inspired by the growing interest in bio‐based monomers and to further expand the available “green” monomer toolkit, we have studied aqueous self‐assembly and solubilization boosting of diblock copolymers of the type poly(ethylene glycol)‐*b*‐poly(farnesyl glycidyl ether), mPEG_114_‐*b*‐PFarGE_m_, as well as their derivatives with a fully hydrogenated hydrophobic block, poly(ethylene glycol)‐*b*‐poly(hexahydrofarnesyl glycidyl ether), mPEG_114_‐*b*‐PHHFarGE_m_, both comprising the naturally occurring terpenoid farnesol.^[^ ^15^ ^]^ It is an intriguing question whether hydrogenation of the double bonds of the terpene influences self‐assembly and solubilization boosting. Fluorescence spectroscopy and light scattering measurements were used to investigate their self‐assembly in aqueous solution to determine whether their critical micelle concentrations (CMCs) systematically depend on the size of the hydrophobic TGE block. Extending the investigation from binary water/copolymer mixtures to pseudo‐ternary microemulsion systems, we examined whether the addition of the novel block copolymers results in an efficiency boosting effect in the microemulsion system H_2_O/NaCl – *n*‐decane – tetraethylene glycol monodecyl ether (C_10_E_4_) and how the boosting effect compares to literature‐known but nonbio‐based block copolymers. Transitioning to more sustainable microemulsion systems, we further studied the influence of mPEG_114_‐*b*‐PHHFarGE_5_ on the quaternary microemulsion system water – isopropyl myristate (IPM) – *n*‐octyl β‐D‐glucopyranoside (C_8_G_1_) – farnesol. Alkyl polyglucosides are commercially available products (e.g., Plantacare) that can be synthesized from renewable materials and are particularly relevant for the personal care sector.^[^ ^49^ ^]^ The oil IPM is nonirritating to humans^[^ ^50^ ^]^ and is frequently used in cosmetics and pharmaceutics. Accompanying SANS experiments were performed to investigate the influence of mPEG_114_‐*b*‐PHHFarGE_5_ on the bicontinuous microstructure of conventional and bio‐based microemulsions, revealing how the novel amphiphilic block copolymer affects the structural order and the effective bending rigidity of the mixed amphiphilic film separating oil and water nanodomains.

## 2 Experimental Section

Details regarding the reagents used, instrumentation, methodical procedures, and monomer synthesis are given in the Supporting Information.

## 3 Results and Discussion

### 3.1 Monomer Synthesis

**Scheme 2:**
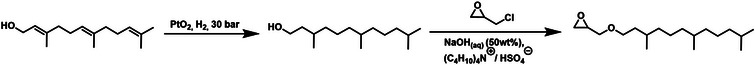
Two‐step synthesis sequence of HHFarGE, starting from the hydrogenation of farnesol and followed by phase transfer catalysis.

The hydrophobic glycidyl ethers presented in this work, namely FarGE and its hydrogenated derivative (HHFarGE), have been synthesized via literature‐known aqueous phase transfer catalysis from epichlorohydrin (ECH) and the respective alcohols.^[^ ^15^, ^51^ ^]^ It is important to note that ECH is available as a fully bio‐sourced option via the Solvay's Epicerol process. In this context, glycerol, accrued as a byproduct in biofuel production, is transformed into “green” ECH at a production scale of 100 kilotons per annum.^[^ ^52^, ^53^, ^54^ ^]^ While the synthesis as well as an in‐depth characterization of FarGE have been previously published,^[^ ^15^ ^]^ HHFarGE represents a novel, fully saturated TGE with increased chain flexibility compared to FarGE. Considering the polarizable π bond in FarGE, a marginally higher hydrophobicity is anticipated for the hexahydrogenated derivative. The synthesis relies on a two‐step sequence (Scheme 2), starting from a commercially available stereoisomeric mixture of farnesol.

**Figure 1:**
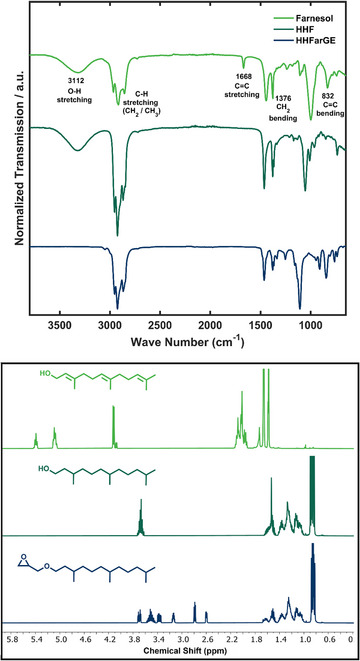
HHFarGE synthesis sequence starting from farnesol. FT‐IR spectra (top) and ^1^H NMR spectra (400 MHz, CDCl_3_, bottom) confirm the successful synthesis.

In the first step, a catalytic hydrogenation relying on platinum(IV) oxide (PtO_2_) transforms farnesol into HHF in quantitative yields (> 90%) without elaborate purification.^[^ ^15^ ^]^ The hydrogen addition to the different farnesol stereoisomers produces a stereoisomeric HHF mixture as characterized by the doublets of carbon resonances in ^13^C NMR analysis (Figure S2).^[^ ^15^, ^55^, ^56^ ^]^ Secondly, the straightforward phase transfer catalysis requires two consecutive distillation steps to afford HHFarGE in high purity. The successful synthesis sequence is further attested by FT‐IR and ^1^H NMR spectra (Figure 1). Hydrogenation leads to the disappearance of the alkene signals of farnesol (5.41 – 5.05 ppm, right) as well as the associated C = C stretching and bending bands at 1668 and 832 cm^−1^ (left), respectively. Furthermore, the single FTIR band near 1400cm^−1^ splits into a doublet as hydrogenation induces a change of carbon hybridization from sp^2^ to sp^3^. The resulting CH_2_ feature generates inequivalent methylene environments, leading to two distinct bending vibrations instead of a single one. The etherification with ECH during the second sequence step is demonstrated as the O‐H stretching band at 3112 cm^−1^ disappears (left, bottom) and the epoxide functionality becomes visible between 3.20 and 2.52 ppm (right, bottom). Detailed NMR analyses of HHF and HHFarGE are displayed in Figures S1–S10. An additional pretreatment of the TGEs with methyl iodide and NaH aims at the deactivation of potential protic impurities and serves as an additional drying process (Scheme S2).^[^ ^15^, ^57^ ^]^

### 3.2 Synthesis and Characterization of mPEG_114_‐*b*‐PHHFarGE_m_ and mPEG_114_‐*b*‐PFarGE_m_ Diblock Copolymers

A key objective of this work is to investigate and compare the amphiphilic diblock copolymers regarding their self‐assembly in aqueous solution as well as in microemulsion systems with respect to polymer hydrophobicity. According to the literature, the amphiphilicity has a significant impact on the strength of the boosting effect.^[^ ^58^ ^]^ Hence, relying on the crown ether‐assisted AROP in bulk, we synthesized two series of diblock copolymers starting from commercial poly(ethylene glycol) monomethyl ether (mPEG_114_) with varying TGE feed ratios.^[^ ^15^, ^32^ ^]^ Maintaining the size of the hydrophilic block, the degree of polymerization (DP) of the hydrophobic block represents a switch to tailor the amphiphilicity and the respective diblock copolymer properties. Table 1 gives an extensive overview of the well‐controlled polymerization results as well as the thermal characterization. The number‐averaged molecular weights (*M*_n_s) of the synthesized amphiphilic diblock copolymers range from 5600 to 8400 gmol^−1^, as determined via ^1^H NMR spectroscopy. Characterization is in line with the targeted value. However, the DP is slightly lower (approximately one TGE unit (*m*)) than targeted for monomer batches not treated with methyl iodide and sodium hydride prior to polymerization. We have previously reported on the deactivation of residual protic impurities in the epoxide, which otherwise leads to homopolymer formation and a lower DP.^[^ ^15^ ^]^ For the mPEG_114_‐*b*‐PHHFarGE_m_ block copolymers, a viscosity increase with incremental DP has been observed.

**Table 1:** Characterization data for the series of amphiphilic mPEG_114_‐*b*‐P(HH)FarGE_m_ block copolymers with respect to molecular weights, dispersity, and thermal properties.

Entry	Sample	*M*_n_^[^ ^a^ ^]^/ g∙mol^−1^	*M*_n_^[^ ^b^ ^]^ / g∙mol^−1^	*Ð* ^[^ ^b^ ^]^	*T*_g_^[^ ^c^ ^]^ / °C	*T*_m_^[^ ^c^ ^]^ / °C	Δ*H*_m_^[^ ^c^ ^]^ / J ∙g^−1^
1	mPEG_114_	5000	5000	1.04	n.d.	61	217
2^[^ ^d^ ^],[^ ^e^ ^]^	mPEG_114_‐*b*‐PFarGE_2_	5600	5800	1.07	‐	59	147
3^[^ ^d^ ^],[^ ^e^ ^]^	mPEG_114_‐*b*‐PFarGE_5_	6400	6300	1.04	−71	56	139
4^[^ ^e^ ^]^	mPEG_114_‐*b*‐PFarGE_6_	6700	6700	1.05	−70	55	99
5^[^ ^d^ ^],[^ ^e^ ^]^	mPEG_114_‐*b*‐PFarGE_7_	7000	7000	1.05	−72	54	93
6^[^ ^e^ ^]^	mPEG_114_‐*b*‐PFarGE_9_	7500	7100	1.05	−71	56	88
7^[^ ^d^ ^],[^ ^e^ ^]^	mPEG_114_‐*b*‐PFarGE_12_	8400	7800	1.04	−73	54	81
8	mPEG_114_‐*b*‐PHHFarGE_3_	5900	5900	1.04	−	57	169
9	mPEG_114_‐*b*‐PHHFarGE_5_	6500	6200	1.05	−67	57	115
10^[^ ^d^ ^]^	mPEG_114_‐*b*‐PHHFarGE_6_	6700	6400	1.05	−65	57	113
11	mPEG_114_‐*b*‐PHHFarGE_7_	7000	6500	1.05	−70	56	104
12	mPEG_114_‐*b*‐PHHFarGE_8_	7300	6600	1.06	−71	56	102
13^[^ ^d^ ^]^	mPEG_114_‐*b*‐PHHFarGE_10_	7900	6900	1.05	−71	55	98

^[a]^Number‐average molecular weight determined via ^1^H NMR spectroscopy.

^[b]^Determined via SEC measurements using DMF as eluent and PEG as standard.

^[c]^Glass transition temperature, melting temperature and melting enthalpy determined by DSC at a heating rate of 10 °C/min. Errors are estimated as ± 2 °C.

^[d]^Monomer batch without treatment with MeI was applied.

^[e]^Data recently published by Schüttner et al.^[^ ^15^ ^]^

To ensure sufficient mixing, the temperature was raised to 120 °C. Surprisingly, this did not promote the occurrence of the well‐known chain transfer reaction.^[^ ^59^ ^]^ We tentatively ascribe this to the highly hydrophobic and sterically demanding character of the monomer, hindering or slowing down the nucleophilic attack in the α‐position to the epoxide ring.^[^ ^60^ ^]^ Accordingly, DOSY analysis shows a single diffusion coefficient for the pure diblock copolymer, verifying the absence of PTGE homopolymer (Figure S16).

The well‐controlled nature of the AROP is demonstrated by the monomodal molecular weight distributions and low dispersities *Ð* (1.04 –1.07) (Figures S24 and S25).^[^ ^61^ ^]^ Furthermore, the variation in TGE block size is illustrated by a continuous shift of the SEC elugrams toward lower elution volumes (higher molecular weights) compared to the mPEG_114_ macroinitiator when the PTGE block size increases. Molecular weights determined via NMR and SEC show a systematic discrepancy with increasing incorporation of TGE units, commonly known for multifunctional PEG.^[^ ^62^ ^]^ This is attributed to a decrease in hydrodynamic radii of the block copolymers in DMF due to the presence of the hydrophobic side chain and polarity difference in comparison to the PEG calibration. Moreover, as the chain collapse is more pronounced for the more hydrophobic mPEG_114_‐*b*‐PHHFarGE_m_, the corresponding homopolymers PHHFarGE_m_ are not soluble in DMF, and thus, SEC characterization in THF as an eluent was carried out (Figure S22). Two homopolymers of each block type, PHHFarGE_m_ and PFarGE_m_, were prepared in analogy to a medium and large‐size PTGE block (Table S1, Figures S22 and S23). The PFarGE_m_ samples have been previously reported. Nevertheless, they are included in Tables 1 and 2 for comparison with the newly prepared hydrogenated samples. Representative in‐depth NMR characterization for mPEG_114_‐*b*‐PHHFarGE_m_ and PHHFarGE_m_ is provided in the Supporting Information, Figures S11–S15 and Figures S17–S21, respectively.

**Table 2:** CMCs of amphiphilic, terpenoid‐derived mPEG_114_‐*b*‐PHHFarGE_m_ and mPEG_114_‐*b*‐PFarGE_m_ diblock copolymers in water at *T* = 23 ± 1 °C, obtained via fluorescence spectroscopy and static light scattering.

Entry	Sample	*M*_n_^[^ ^a^ ^]^/ g∙mol^−1^	*M*_n,TGE block_^[^ ^a^ ^]^/ g∙mol^−1^	HLB^[^ ^b^ ^]^	CMC_FS_^[^ ^c^ ^]^ / mgL^−1^	CMC_LS_^[^ ^d^ ^]^ / mgL^−1^
1^[^ ^e^ ^]^	mPEG_114_‐*b*‐PFarGE_2_	5600	560	18.0	90 ± 5	−
2^[^ ^e^ ^]^	mPEG_114_‐*b*‐PFarGE_5_	6400	1250	16.1	53 ± 4	44 ± 7
3^[^ ^e^ ^]^	mPEG_114_‐*b*‐PFarGE_6_	6700	1670	15.0	25 ± 2	−
4^[^ ^e^ ^]^	mPEG_114_‐*b*‐PFarGE_7_	7000	1950	14.4	21 ± 1	−
5^[^ ^e^ ^]^	mPEG_114_‐*b*‐PFarGE_9_	7500	2500	13.4	15 ± 1	16 ± 4
6^[^ ^e^ ^]^	mPEG_114_‐*b*‐PFarGE_12_	8400	3340	12.0	14 ± 1	−
7	mPEG_114_‐*b*‐PHHFarGE_3_	5900	860	17.1	56 ± 3	−
8	mPEG_114_‐*b*‐PHHFarGE_5_	6500	1420	15.6	28 ± 2	24 ± 5
9	mPEG_114_‐*b*‐PHHFarGE_6_	6700	1710	14.9	24 ± 1	−
10	mPEG_114_‐*b*‐PHHFarGE_7_	7000	1990	14.3	22 ± 1	16 ± 3
11	mPEG_114_‐*b*‐PHHFarGE_8_	7300	2280	13.7	18 ± 1	−
12	mPEG_114_‐*b*‐PHHFarGE_10_	7900	2850	12.7	14 ± 1	−

^[a]^Number‐averaged molecular weight of diblock copolymers and FarGE block determined via ^1^H NMR spectroscopy.

^[b]^Hydrophilic‐lipophilic balance calculated according to Griffin: HLB = 20 (1‐ *M*_lipophilic_ / *M*_lipophilic+hydrophilic_) = 20 (1‐ *M*_n,TGE block_/ *M*_n_).^[^ ^65^ ^]^

^[c]^CMC determined via fluorescence spectroscopy.

^[d]^CMC determined via static light scattering.

^[e]^Data partially published by Schüttner et al.^[^ ^15^ ^]^

Differential scanning calorimetry (DSC) measurements of both the diblock copolymers and homopolymers were carried out to evaluate the thermal properties. All diblock copolymers feature a distinct single melting endotherm with a melting temperature (*T*_m_) ranging between 54 and 59 °C (Figures S26–S28). Compellingly, the absence of a second melting endotherm indicates that the branching within the side chain structure impedes its crystallization.^[^ ^15^, ^60^ ^]^ The marginal decrease of the *T*_m_ compared to the crystalline mPEG_114_ macroinitiator (Table 1, Entry 1) reflects the block copolymer architecture, indicating nanosegregated domains due to block incompatibility and mPEG crystallization. For mPEG_114_‐*b*‐PFarGE_m_ and mPEG_114_‐*b*‐PHHFarGE_m_, the glass transition temperature (*T*_g_) values vary between ‐70 and ‐73 °C and ‐65 to ‐71 °C, respectively.^[^ ^15^ ^]^ The results are in line with the determined *T*_g_s of the amorphous, low‐DP homopolymers (Table S1 and Figure S29). Equally, the detection of a single *T*_g_ for the PTGE block further suggests nano‐segregated blocks driven by the strong PEG crystallization, resulting in the absence of the *T*_g_ of the PEG block.^[^ ^15^ ^]^

### 3.3 Self‐Assembly in Aqueous Solution

As a prerequisite for the envisaged utilization of the novel poly(ethylene glycol)‐*b*‐poly(terpenyl glycidyl ether) copolymers as efficiency boosters in microemulsions, their amphiphilic properties were first studied in aqueous solution. Similar to conventional surfactants, amphiphilic diblock copolymers can self‐assemble in water.^[^ ^63^, ^64^ ^]^ A variety of ordered morphologies, such as spherical or cylindrical micelles or vesicles, can be formed when exceeding a certain amphiphile concentration.^[^ ^64^, ^65^ ^]^ We have recently shown that dynamic light scattering experiments of mPEG_114_‐*b*‐PFarGE_m_ copolymers performed well above the CMC reveal monomodal micelles for lower degrees of polymerization (i.e., few FarGE units), while heterogeneous aggregate sizes were found for the more hydrophobic counterparts.^[^ ^15^ ^]^ However, to be consistent with the notation widely used in the literature for other conventional and polymeric amphiphiles, we use the term CMC instead of the more general term “critical aggregation concentration (CAC)”.

### 3.4 Determination of Critical Micelle Concentrations (CMC)

**Figure 2:**
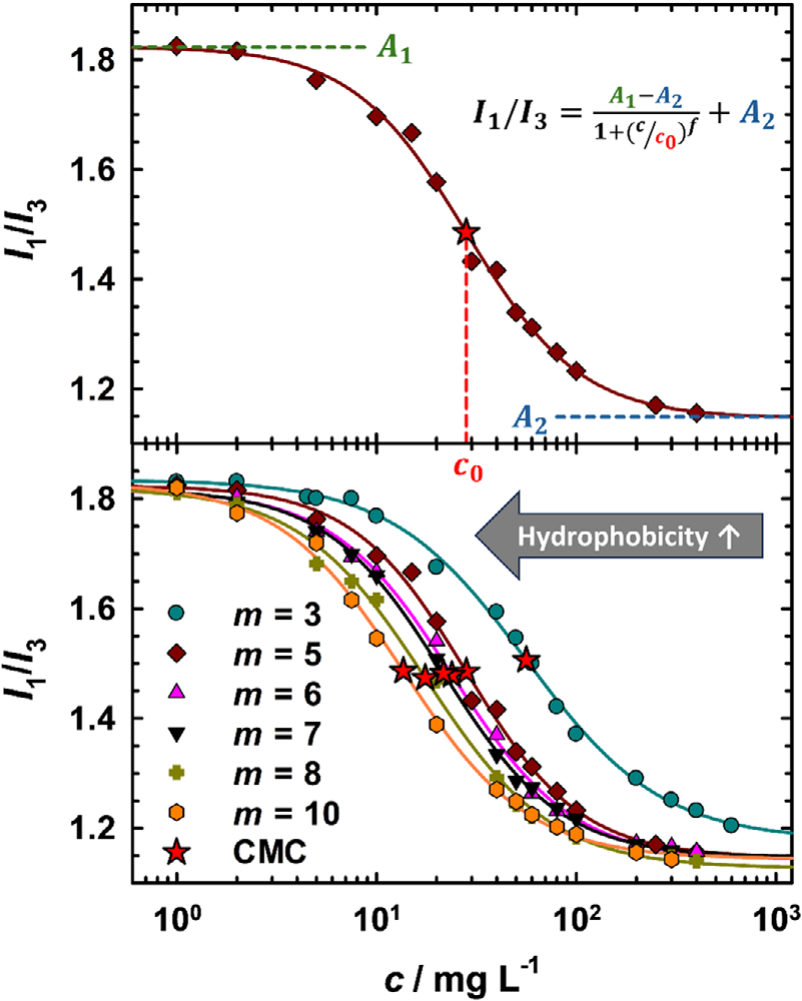
Concentration‐dependent micellization of mPEG_114_‐*b*‐PHHFarGE_m_ in water, studied by fluorescence spectroscopy (FS) measurements at *T* = 23 ± 1 °C, with pyrene *I*_1_/*I*_3_ ratio plotted versus polymer concentration *c*. CMCs are denoted with red stars. Top: FS data and demonstration of the power law fitting function for mPEG_114_‐*b*‐PHHFarGE_5_. Bottom: FS measurements for various mPEG_114_‐*b*‐PHHFarGE_m_ reveal a systematic CMC shift to lower concentrations with increasing hydrophobic block size *m*.

As polymer CMCs are generally expected to be considerably lower than those of conventional surfactants, we used fluorescence spectroscopy as a highly sensitive method to study the aggregation behavior of the mPEG_114_‐*b*‐P(HH)FarGE_m_ block copolymers in aqueous solution, taking advantage of the sensitivity of the pyrene emission spectrum to the polarity of its microenvironment.^[^ ^15^, ^66^, ^67^, ^68^, ^69^, ^70^ ^]^ Figure 2, top, exemplarily shows the intensity ratio $I_{1}/I_{3}$ of the maximum of the first and third pyrene vibronic bands as a function of concentration for the block copolymer mPEG_114_‐*b*‐PHHFarGE_5_. A sigmoidal trend of the $I_{1}/I_{3}$ ratio is found, as has been observed for conventional surfactants^[^ ^69^ ^]^ as well as for the structurally similar mPEG_114_‐*b*‐PFarGE_m_ copolymers.^[^ ^15^ ^]^ For common surfactants, in view of their overall higher CMCs, an exponential fitting function is usually used.^[^ ^69^ ^]^ We have recently shown that this approach is disadvantageous if amphiphile concentrations are very low because minimizing least squares can result in negative CMCs.^[^ ^15^ ^]^ Instead, we introduced a power law given as

(1) $$I_{1}/I_{3}=\frac{A_{1}-A_{2}}{1+{\left(c/c_{0}\right)}^{f}}+A_{2},$$

which is beneficial because it is applicable solely to positive concentrations and further reduces the least squares. $A_{1}$ and $A_{2}$ are the $I_{1}/I_{3}$ limits toward *c* → 0 and *c* → ∞, respectively, f quantifies the steepness of the sigmoid, and $c_{0}$ is the concentration at the inflection point of the sigmoid, which is defined as the CMC.^[^ ^15^ ^]^ $I_{1}/I_{3}$ reaches a value of $A_{1}\;\approx$ 1.8 at low mPEG_114_‐*b*‐PHHFarGE_5_ concentrations, which is in agreement with studies on aqueous amphiphile solutions using a similar excitation wavelength.^[^ ^15^, ^67^, ^69^, ^70^ ^]^ This is not surprising considering that $A_{1}$ corresponds to infinite dilution and should therefore not depend on the solute, but the solvent. With increasing copolymer concentration, pyrene partitions into the hydrophobic micellar core, resulting in a decreasing $I_{1}/I_{3}$ ratio down to $A_{2}\;\approx$ 1.2. The inflection point of the sigmoid is denoted with a red star and gives a CMC of 28 ± 2 mgL^−1^ for mPEG_114_‐*b*‐PHHFarGE_5_. Additional static and dynamic light scattering experiments (SLS/DLS) recorded with a laser wavelength of λ = 561 nm at a scattering angle of θ = 90° were performed for some copolymers to verify the results obtained from fluorescence spectroscopy. Experimental details and results are provided in the Supporting Information (cf. Figure S34) and Table 2.

### 3.5 Influence of the Degree of Polymerization on CMCs

**Figure 3:**
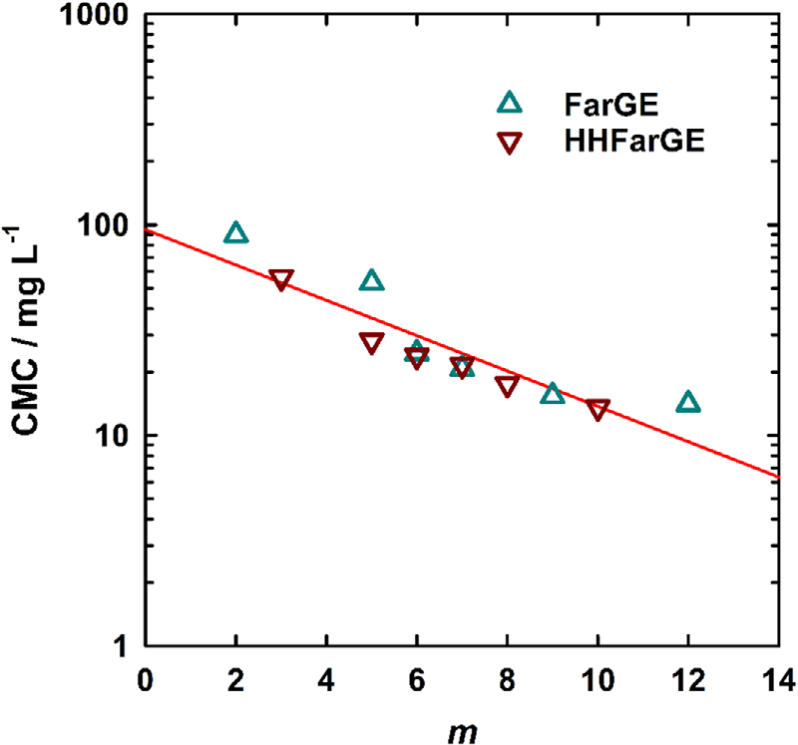
CMCs determined via fluorescence spectroscopy as a function of hydrophobic block size *m* for mPEG_114_‐*b*‐PFarGE_m_ (teal upward triangles) and mPEG_114_‐*b*‐PHHFarGE_m_ (brown downward triangles).

Adjustability of the degree of polymerization of our copolymers offers facile tuning of amphiphilicity. Available literature data of different amphiphiles, which includes conventional surfactants as well as amphiphilic block copolymers, indicates that the hydrophobic part of the amphiphile serves as the primary influence on micellization, largely governed by the hydrophobic effect.^[^ ^71^, ^72^, ^73^, ^74^, ^75^, ^76^ ^]^ Given the wide range of copolymers with different numbers of TGE units, this should also manifest itself in an altered self‐assembly in aqueous solution and therefore a varying CMC. Figure 2, bottom, shows the $I_{1}/I_{3}$ ratio versus polymer concentration plots for all block copolymers of the mPEG_114_‐*b*‐PHHFarGE_m_ series. Qualitatively uniform trends, in particular with regard to the sigmoidal upper ($A_{1}\approx$ 1.8) and lower ($A_{2}\approx$ 1.2) limit, are found irrespective of hydrophobicity. On a quantitative level, a systematic trend of the inflection point of the sigmoid, that is, the CMC, is clearly visible. An increasing number of TGE units (*m*) shifts the sigmoid and therefore the inflection points toward lower concentrations. Figure 3 emphasizes that a logarithmic plot of the CMCs against *m* results in a linear reduction of log(CMC) with increasing hydrophobic block size. Similarly, a decreasing CMC with increasing amphiphile hydrophobicity has been observed for conventional nonionic surfactants^[^ ^71^, ^72^, ^73^ ^]^ as well as for ionic liquids^[^ ^74^ ^]^ and polymeric systems,^[^ ^75^ ^]^ which can – irrespective of the amphiphile – be explained based on the hydrophobic effect. The increasing size of the hydrophobic part of the molecule enhances the entropic benefit during micelle formation, as more water molecules are released from their ordered arrangement around the hydrophobic moieties, which is why self‐assembly occurs at lower concentrations.^[^ ^77^, ^78^ ^]^ CMCs for all block copolymers investigated in this work, including light scattering results, are compiled in Table 2.

Intriguingly, Figure 3 highlights that the CMCs of the nonhydrogenated mPEG_114_‐*b*‐PFarGE_m_ copolymers (teal upward triangles) almost match those of the hydrogenated mPEG_114_‐*b*‐PFarGE_m_ derivatives (brown downward triangles). This observation emphasizes that hydrogenation of the farnesyl side chain does not lead to significantly different behavior of the copolymers in aqueous solution, at least at low concentrations. Consequently, it is furthermore clear that the influence of hydrogenation on block copolymer hydrophobicity is much smaller than the introduction of another TGE unit, which is readily understood when considering that each unit contains another C_15_ chain, providing a significant increase in hydrophobicity. Fluorescence spectroscopy data for the individual mPEG_114_‐*b*‐P(HH)FarGE_m_ copolymers and the influence of the degree of polymerization can be found in Figures S30–S33. Comparison to previously reported CMC values of PEG‐based diblock copolymers shows agreement with the results obtained in this work, considering that the CMC generally resides in a lower range when a more hydrophobic block is present.^[^ ^62^, ^67^, ^79^, ^80^ ^]^ Overall, the data corroborate the highly amphiphilic character of the diblock copolymers, while the potential to incorporate hydrophobic compounds is demonstrated. This motivates the transition to the formulation of microemulsions, where a nonpolar oil is introduced as a hydrophobic component, and could further pave the way to various applications, such as nanoparticle synthesis in confinement, drug delivery, cosmetics, or cleaning.

### 3.6 Efficiency Boosting in Conventional Microemulsions

Having observed the micellization of the mPEG_114_‐*b*‐P(HH)FarGE_m_ block copolymers in aqueous solutions, the influence of these copolymers on ternary water – oil – nonionic surfactant microemulsion systems was subsequently investigated. One‐phase microemulsions can be formed if there is a sufficiently high amount of surfactant to completely solubilize water and oil. As demonstrated by Jakobs et al. with their poly(ethylene glycol)‐*b*‐poly(propylene) (PEG_n_‐*b*‐PEP_m_) copolymers,^[^ ^33^ ^]^ the solubilization efficiency of surfactants can be drastically enhanced by adding suitable amphiphilic copolymers as cosurfactants. Known as the “efficiency boosting effect,”^[^ ^33^ ^]^ this can be attributed to the adsorption of amphiphilic copolymers into the surfactant membrane, from where they extend in a mushroom‐like conformation into the oil and water sub‐phases, respectively.^[^ ^34^, ^35^, ^36^, ^37^ ^]^

To determine the applicability of mPEG_114_‐*b*‐P(HH)FarGE block copolymers as cosurfactants and efficiency boosters, *T*(γ) sections through the Gibbs phase prism (“fish cuts”) were first measured in the model microemulsion system H_2_O/NaCl – *n*‐decane – tetraethylene glycol monodecyl ether (C_10_E_4_), utilizing equal volumes of water and oil (ϕ = 0.5). Minor amounts of NaCl (0.1 wt% in water, ε = 0.001) were added to screen undesired electrostatic interactions that might result from ionic impurities in the copolymer. The same microemulsion system (albeit without NaCl) was studied in the original boosting study by Jakobs et al.,^[^ ^33^ ^]^ which allows for a comparison with their copolymers. Adjusting the respective copolymer content δ, with δ denoting the mass fraction of the copolymer in the amphiphilic mixture, samples were prepared at high amphiphile concentrations γ and subsequently diluted until close to the optimum point (also called “$\tilde{X}$ point” or “fish tail point”), characterized by the phase inversion temperature $\tilde{T}$ and $\tilde{\gamma }$, with the latter denoting the minimum amphiphile mass fraction required to form a one‐phase microemulsion. A low $\tilde{\gamma }$ therefore indicates a high solubilization efficiency. More details on the mentioned parameters and the experimental procedure are provided in the Supporting Information.

**Figure 4:**
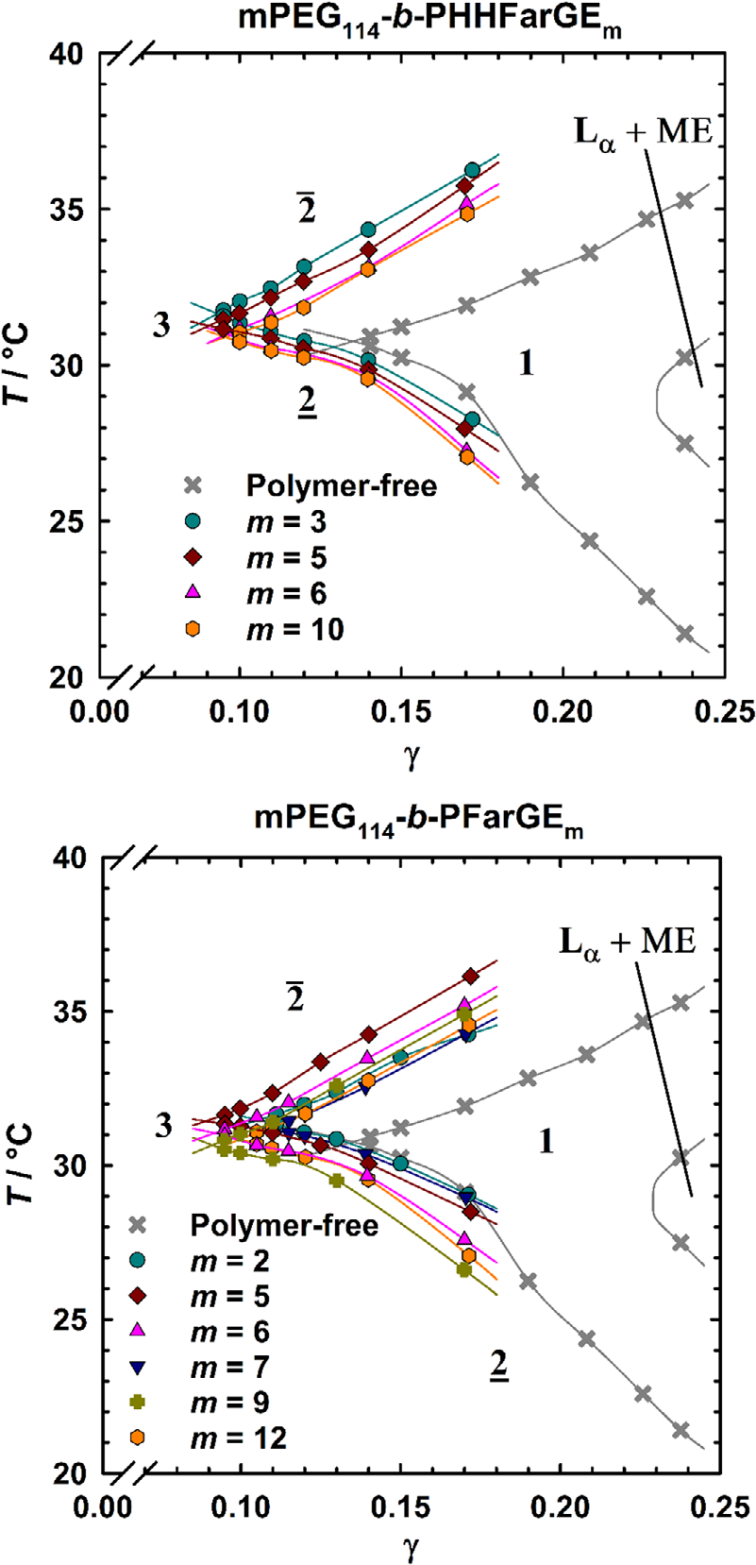
*T*(γ) diagrams of the system H_2_O/NaCl – *n*‐decane – C_10_E_4_/mPEG_114_‐*b*‐P(HH)FarGE_m_, recorded at constant oil‐water‐ratio (*ϕ* = 0.5) and salinity (*ε* = 0.001) with 5 wt.% of copolymer in the amphiphile mixture (δ = 0.05). Top: mPEG_114_‐*b*‐PHHFarGE_m_ Bottom: mPEG_114_‐*b*‐PFarGE_m_. The strong shift of $\tilde{\gamma }$ upon the addition of the different block copolymers demonstrates a marked efficiency boosting effect, with a similar boosting strength for both copolymer types.

Fish cuts for the symmetric microemulsion systems (*ϕ* = 0.5) containing mPEG_114_‐*b*‐PHHFarGE_m_ (top) or mPEG_114_‐*b*‐PFarGE_m_ (bottom) copolymers are shown in Figure 4, with the gray crosses being the copolymer‐free reference system, which was found to be in agreement with literature data.^[^ ^32^, ^33^ ^]^ The observed phase behavior is typical for nonionic surfactant systems:^[^ ^81^ ^]^ At low temperatures, an oil‐in‐water microemulsion co‐exists with an oil excess phase, denoted as $\underline{2}$. When the temperature is raised and γ > $\tilde{\gamma }$, a one‐phase microemulsion (1) is obtained. If the surfactant concentration is not high enough to fully solubilize water and oil (γ < $\tilde{\gamma }$), a three‐phase region (3) is found instead. Increasing temperature decreases the mutual solubility of the nonionic surfactant and water, eventually resulting in a water‐in‐oil microemulsion that co‐exists with a water excess phase, denoted as $\bar{2}$. At high surfactant concentrations, a two‐phasic co‐existence of lamellar and microemulsion phase (L_α_ + ME) is embedded into the one‐phase region.

It is visible from Figure 4, top that the addition of all mPEG_114_‐*b*‐PHHFarGE_m_ results in a significant efficiency increase, corresponding to a decreasing value of $\tilde{\gamma }$. Upon replacement of 5 wt.% C_10_E_4_ with mPEG_114_‐*b*‐PHHFarGE_m_, $\tilde{\gamma }$ shifts from 0.135 in the polymer‐free system to approximately $\tilde{\gamma }$ ≈ 0.085–0.095 for the copolymer‐containing systems, indicating a strong efficiency gain. Indeed, such a gain may be expected as the basic prerequisites for efficiency boosting are fulfilled,^[^ ^82^ ^]^ namely that the chemical nature of the copolymer blocks and the resulting amphiphilicity ensure the adsorption of the copolymer in the surfactant membrane. In addition, the size of the blocks is chosen to fit into the solvent domains of the microemulsion and the concentration of the copolymer is low enough to prevent their overlap (brush regime). Intriguingly, however, unlike found for copolymers with mushroom‐like blocks, it is not the copolymers with the largest blocks that led to the largest gain in solubilization performance, but rather those with a medium hydrophobic block size. Based on this observation, it appears that the (HH)FarGE block, unlike the PEG block in the H_2_O domains, does not form a mushroom conformation in the *n*‐decane domains. Sophisticated contrast variation SANS measurements with partially deuterated copolymers would be necessary to investigate the structure of the block and clarify whether, due to the FarGE side chains, this block is located rather close to the amphiphilic film in a manner similar to comb‐like polymers^[^ ^83^ ^]^ and only slightly extends into the *n*‐decane domains. If this hypothesis is correct, similar to so‐called sticker polymers,^[^ ^45^ ^]^ the hydrophobic (HH)FarGE block would thus mainly ensure that the copolymers are forced into the amphiphilic film. Thus, for this new class of mPEG_114_‐*b*‐P(HH)FarGE block copolymers, improved efficiency could just be achieved by increasing the size of the PEG block.

It is clear from Figure 4, bottom, that similar boosting strengths are found for the nonhydrogenated mPEG_114_‐*b*‐PFarGE_m_ copolymers, indicating that the complete hydrogenation of the farnesyl chain does not have any noticeable impact on the solubilization boosting of the copolymers. For both copolymer types investigated in this work, the strongest efficiency boosting was found with (HH)FarGE blocks in the range of *m* = 5–6.

As opposed to efficiency, the optimum temperature ($\tilde{T}$) is only marginally affected by the addition of the copolymers, showing deviations of less than 1 K compared to the copolymer‐free reference system. A closer look at the temperature effect of mPEG_114_‐*b*‐P(HH)FarGE_m_ copolymers shows that, as expected,^[^ ^24^ ^]^ copolymers with shorter hydrophobic blocks produce a somewhat stronger shift toward higher temperatures.

**Figure 5:**
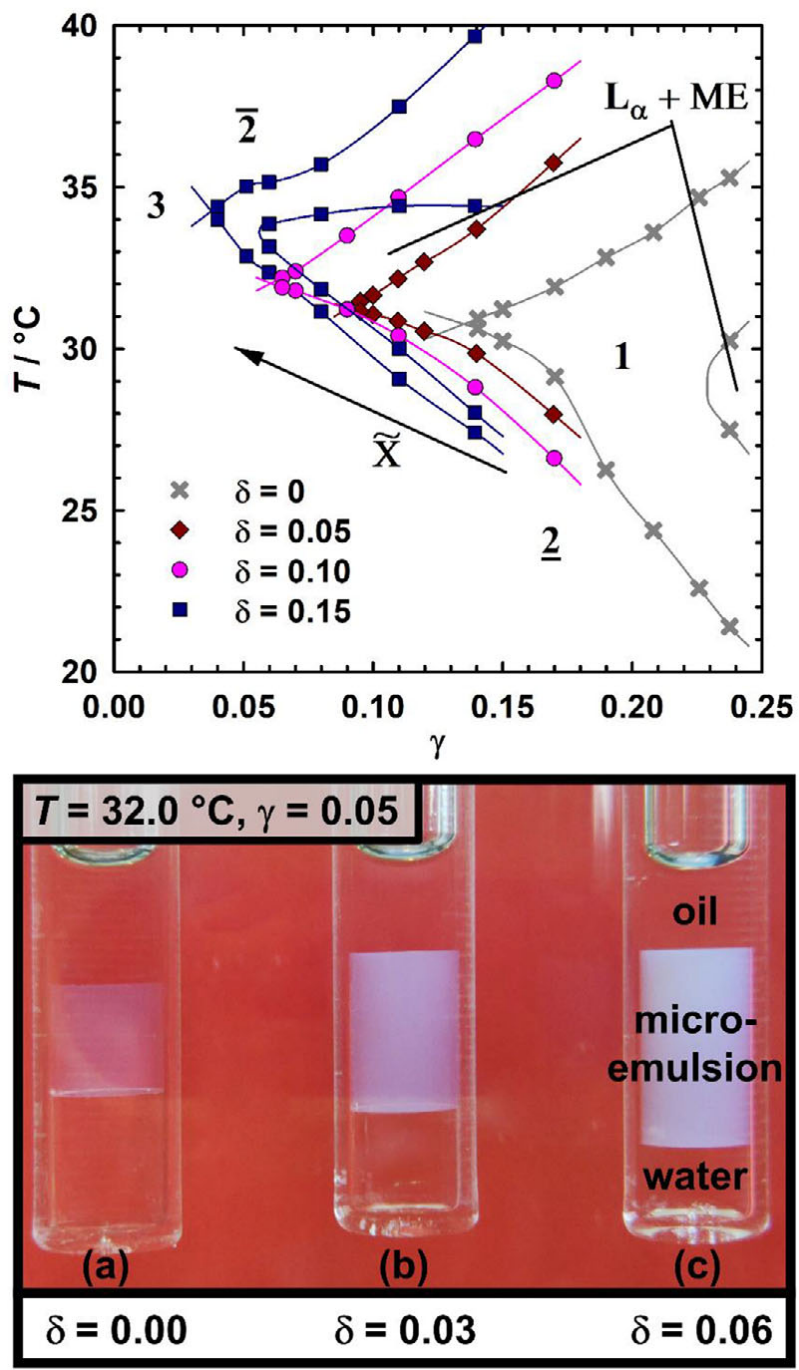
Top: *T*(γ) diagrams of the system H_2_O/NaCl – *n*‐decane – C_10_E_4_/mPEG_114_‐*b*‐PHHFarGE_5_, recorded at constant oil‐water‐ratio (*ϕ* = 0.5) and salinity (*ε* = 0.001) with varying amounts of copolymer in the amphiphile mixture (*δ*), showing the continuing boosting effect with increasing *δ*. Bottom: Boosting effect of mPEG_114_‐*b*‐PHHFarGE_5_ at constant amphiphile mass fraction (*γ* = 0.05) and *T* = 32 °C, close to the phase inversion temperature of the polymer‐free system. Polymer concentrations in the amphiphilic mixture are (a) *δ* = 0.00, (b) *δ* = 0.03, and (c) *δ* = 0.06. With increasing *δ*, the microemulsion phase grows while oil and water excess phases shrink. The middle microemulsion phases appear more turbid because of larger water/oil domain sizes, which result in an increased scattering.

After proving the boosting potential of the amphiphilic mPEG_114_‐*b*‐P(HH)FarGE_m_ block copolymers, two of the most efficient copolymers, mPEG_114_‐*b*‐PFarGE_5_ and mPEG_114_‐*b*‐PHHFarGE_5_, were selected to investigate the influence on efficiency at higher polymer concentrations δ. Fish cuts for the system H_2_O/NaCl – *n*‐decane – C_10_E_4_/mPEG_114_‐*b*‐PHHFarGE_5_ are shown in Figure 5 (top). Results with mPEG_114_‐*b*‐PFarGE_5_ can be found in Figure S36, revealing that the two block copolymer types again only show marginal differences regarding their phase behavior, as was already observed for δ = 0.05. By gradually increasing the content of mPEG_114_‐*b*‐PHHFarGE_5_ in the amphiphilic mixture, the amphiphile amount needed for complete solubilization could be strongly reduced to 3.7 wt.% at δ = 0.15. As a consequence of this drastically reduced amphiphile demand, the water/oil nanodomains in the bicontinuous microemulsion become significantly larger (as will be quantified later via SANS). The strong boosting effect as well as the increasing domain sizes, can be conveniently visualized by preparing samples with different polymer concentrations close to the phase inversion temperature at a fixed amphiphile concentration in the three‐phase region of the microemulsion. As presented in Figure 5, bottom, increasing the concentration of mPEG_114_‐*b*‐PHHFarGE_5_ leads to a pronounced growth of the middle phase (i.e., the microemulsion phase) at the expense of water and oil excess phases. An increasingly milky appearance stems from a pronounced scattering due to the increasing domain sizes.

While initial copolymer addition suppresses the formation of liquid‐crystalline phases, the highest polymer concentration shows a pronounced co‐existence of lamellar phase and microemulsion up to low γ, caused by the copolymer‐induced changes of the bending elastic constants κ and $\bar{\kappa }$ of the amphiphilic film, which has also been observed in other studies.^[^ ^32^, ^33^, ^47^ ^]^ Furthermore, the phase inversion temperature $\tilde{T}$ is about 2 K higher than for the polymer‐free sample, continuing the anticipated trend to higher temperatures resulting from the hydrophilic PEG block being larger than the hydrophobic PHHFarGE_5_ block.

**Figure 6:**
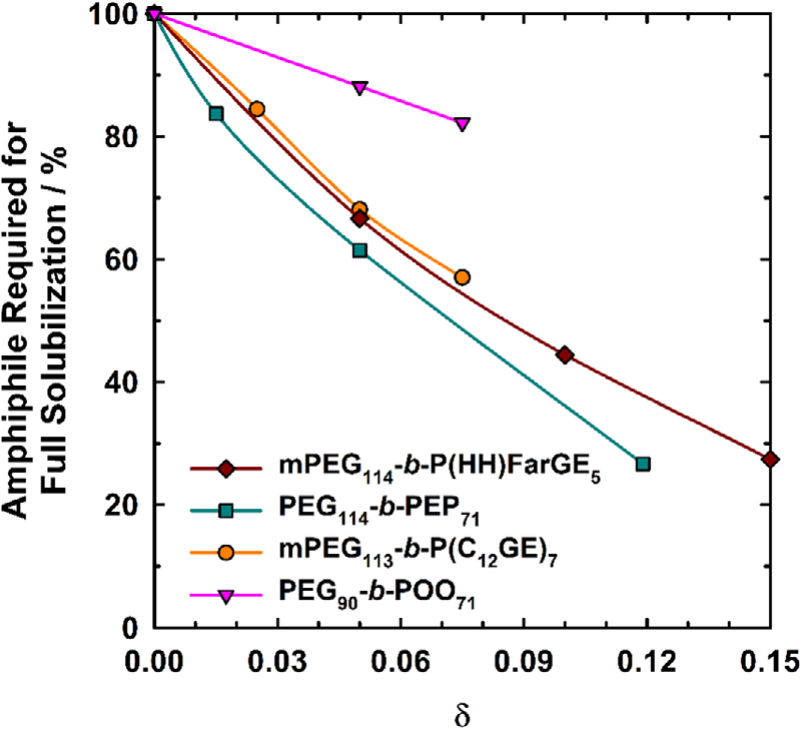
Efficiency boosting effect of the novel mPEG_114_‐*b*‐PHHFarGE_5_ synthesized in this work (brown diamonds) in comparison to different literature‐known block copolymers in the microemulsion system H_2_O/NaCl – *n*‐decane – C_10_E_4_. Within measurement and analytical accuracy, data for mPEG_114_‐*b*‐PFarGE_5_ are in quantitative agreement with those of its hydrogenated analog. Teal squares: PEG_114_‐*b*‐PEP_71_ investigated by Jakobs et al. (no NaCl used).^[^ ^33^ ^]^ Orange circles: mPEG_113_‐*b*‐P(C_12_GE)_7_ investigated by Schneider et al.^[^ ^32^ ^]^ Pink triangles: PEG_90_‐*b*‐POO_71_ investigated by Schneider.^[^ ^84^ ^].^

In order to assess the boosting effect of the novel bio‐based amphiphilic FarGE block copolymers as compared to that of the poly(ethylene glycol)‐*b*‐poly(ethylene propylene)^[^ ^33^ ^]^ and the mPEG_113_‐*b*‐P(CO_2_)C_i_GE_m_ copolymers,^[^ ^32^ ^]^ the amount of amphiphile required for complete mutual water/oil solubilization (calculated via $\tilde{\gamma }/{\tilde{\gamma }}_{0}$, where the index 0 refers to the polymer‐free system) is plotted in dependence on the copolymer mass fraction in the amphiphilic mixture in Figure 6. Notably, replacing 15 wt. % of C_10_E_4_ with mPEG_114_‐*b*‐PHHFarGE_5_ reduces the amount of amphiphile required for the complete solubilization of water and oil by over 70 %, which is in quantitative agreement with the boosting effect of the nonhydrogenated mPEG_114_‐*b*‐PFarGE_5_ (not shown). It can be seen that the observed boosting effect is comparable to the boosting effect of mPEG_114_‐*b*‐PAlkGE_7_ block copolymer,^[^ ^32^ ^]^ much stronger than for poly(ethylene glycol)‐*b*‐poly(octylene oxide) PEG_90_‐*b*‐POO_71_, but somewhat less pronounced than for the PEG_114_‐*b*‐PEP_71_ copolymer.^[^ ^33^ ^]^ The fact that the gradient of the curve and thus the increase in the boosting effect decreases with increasing *δ* can be attributed to the high steric demand of the copolymers, which leads to limited polymer coverage and restricted flexibility of the amphiphilic film. Additionally, the fish head region of the *T*(γ) cut does not extend up to γ = 0, since some of the surfactant is always monomerically dissolved in water and mainly oil, further limiting the relative maximum surfactant savings.

A more profound description of the boosting effect, which even allows its scaling, is achieved by theoretically analyzing the influence of the copolymers adsorbed on the thermally fluctuating surfactant film.^[^ ^34^, ^35^, ^36^, ^37^, ^38^, ^39^, ^40^ ^]^ This concept has been successfully employed for PEG_n_‐*b*‐PEP_m_^[^ ^34^, ^35^, ^38^, ^39^ ^]^ and mPEG_113_‐*b*‐PAlkGE_m_ copolymers,^[^ ^32^ ^]^ revealing a similar scaling factor for *n*‐decane microemulsions, while a different scaling factor was found for an *n*‐octacosane microemulsion being studied at higher temperature, at which the copolymers might behave more like ideal chains.^[^ ^32^, ^34^, ^48^ ^]^ However, applying this scaling relation requires information on the end‐to‐end distances of hydrophilic and hydrophobic blocks in water and oil, respectively, with the hydrophobic P(HH)FarGE_m_ end‐to‐end distances not being available to us. Considering the structural similarity to the mPEG_113_‐*b*‐PAlkGE_m_ copolymers^[^ ^32^ ^]^ combined with the boosting effect observed in this work, it can be assumed that the mPEG_114_‐*b*‐P(HH)FarGE_m_ copolymers used here would reveal a similar scaling factor as the PEG_n_‐*b*‐PEP_m_^[^ ^34^, ^35^, ^38^, ^39^ ^]^ and mPEG_113_‐*b*‐PAlkGE_m_ copolymers^[^ ^32^ ^]^ in *n*‐decane microemulsions.

### 3.7 Efficiency Boosting in Bio‐Based Microemulsions

With the aim of formulating more sustainable microemulsions and given that the used alcohol ethoxylates are typically not synthesized from renewable sources and have been thoroughly assessed with regard to their potential ecotoxicity,^[^ ^85^, ^86^ ^]^ C_10_E_4_ was replaced by a sugar surfactant, *n*‐octyl β‐D‐glucopyranoside (C_8_G_1_), which is readily accessible from renewable feedstocks. With the same motivation, *n*‐decane was replaced by isopropyl myristate (IPM), which is commonly used in cosmetic and pharmaceutical preparations as a moisturizer that is nonirritating to the skin.^[^ ^50^ ^]^ Note that investigations of microemulsions of the type H_2_O – IPM – sugar surfactant (plus alcohol as cosurfactant) can be found in the literature for a range of pure and commercially available technical‐grade sugar surfactants.^[^ ^87^, ^88^ ^]^

Combining the advantages of sustainable microemulsions and solubilization‐enhancing copolymers, we therefore investigated the pseudo‐quaternary system H_2_O – IPM – C_8_G_1_/mPEG_114_‐*b*‐PHHFarGE_5_ – farnesol, with the latter being chosen as a suitable alcohol/cosurfactant to match the terpenyl side chain of the block copolymer and to reduce the high curvature of the amphiphilic film around the oil resulting from the use of the highly hydrophilic sugar surfactant. Note that in contrast to the first set of experiments with the conventional microemulsion system, here the phase inversion was accomplished by the isothermal addition of the hydrophobic cosurfactant farnesol (*T* = 25 °C). This accounts for the fact that microemulsions containing sugar surfactants are typically less sensitive to the influence of temperature than those with alcohol ethoxylates because the strong hydrogen bonds between sugar head group and water prevent dehydration.^[^ ^89^ ^]^

**Figure 7:**
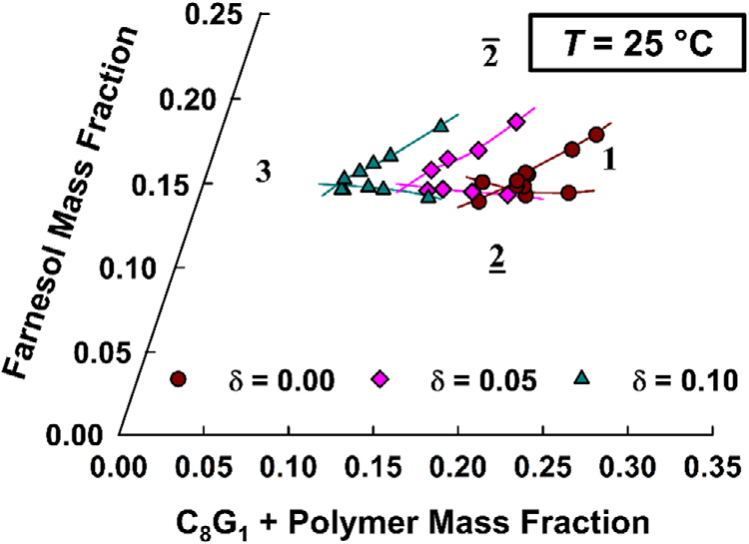
Isothermal (*T* = 25 °C) section through the phase tetrahedron of the system H_2_O – IPM – C_8_G_1_/mPEG_114_‐*b*‐PHHFarGE_5_ – farnesol, in which the farnesol mass fraction is plotted as a function of the total amphiphile mass fraction at equal volumes of water and IPM (*ϕ*=0.5). Increasing the mass fraction (*δ*) of copolymer in the C_8_G_1_/copolymer mixture shifts the phase boundaries to lower amphiphile mass fractions, indicating the solubilization efficiency boosting of the mPEG_114_‐*b*‐PHHFarGE_5_ copolymer.

Figure 7 shows a section through the phase tetrahedron in which the farnesol mass fraction is plotted as a function of the total amphiphile mass fraction at equal volumes of water and IPM (ϕ = 0.5), and in which the phase diagrams for different ratios (δ) of mPEG_114_‐*b*‐PHHFarGE_5_ in the C_8_G_1_/mPEG_114_‐*b*‐PHHFarGE_5_ mixture are given. For the copolymer‐free system (brown circles), around 18 wt.% of C_8_G_1_ and another 15 wt.% of farnesol are required to fully solubilize water and IPM into a one‐phase microemulsion. Using 1,2‐octanediol as cosurfactant, Peng et al. required 15.4 wt.% of C_8_G_1_ but almost 19.5 wt.% of 1,2‐octanediol for complete solubilization.^[^ ^88^ ^]^ It is intuitive to explain the larger amount of 1,2‐octanediol required by its relatively high hydrophilicity compared to farnesol. However, the effect of the long‐chain farnesol is less pronounced than might be anticipated. Our understanding is that farnesol and IPM have a higher mutual solubility than 1,2‐octanediol and IPM. Therefore, some of the farnesol molecules are not available to function as a hydrophobic cosurfactant at the interface; instead, farnesol is partly dissolved in the oil phase, leading to a net loss of solubilization efficiency and an unexpectedly high amount of required cosurfactant.

Further comparing these observations to the H_2_O/NaCl – *n*‐decane – C_10_E_4_ microemulsion system, it can be assumed that the limiting factor for solubilization of the oil is not only its hydrophobicity, but also its size. Both IPM with its long alkyl chain and C_8_G_1_ with its sugar head group are sterically more demanding than their conventional counterparts, *n*‐decane and C_10_E_4_. Additionally, considering that surfactant hydrophobicity is typically associated with an increased solubilization efficiency,^[^ ^25^ ^]^ the shorter chain length of C_8_G_1_ compared to C_10_E_4_ paired with a stronger incompatibility with the long‐chain IPM impedes the solubilization of the nonpolar component in the bio‐based formulation.

Regarding the boosting potential, it becomes evident from Figure 7 that mPEG_114_‐*b*‐PHHFarGE_5_ can indeed function as a suitable efficiency booster in this sustainable microemulsion formulation. At δ = 0.10, the amount of C_8_G_1_ and mPEG_114_‐*b*‐PHHFarGE_5_ necessary for the complete solubilization of water and IPM into a one‐phase microemulsion is halved. Intriguingly, the lower phase boundary ($\underline{2}$ → 1) is almost unaffected by polymer addition. Irrespective of C_8_G_1_/mPEG_114_‐*b*‐PHHFarGE_5_ content and ratio, approximately 15 wt.% of farnesol is required to reach the one‐phase region, which serves as further proof for the assumption of a high mutual solubility of farnesol and IPM. In contrast, the upper phase boundary (1 → $\bar{2}$) shift closely resembles what was found for the H_2_O/NaCl – *n*‐decane – C_10_E_4_ microemulsion. Even though the efficiency boosting of the IPM/C_8_G_1_/farnesol system was exemplified by means of mPEG_114_‐*b*‐PHHFarGE_5_, it should be emphasized that the influence of other mPEG_114_‐*b*‐PHHFarGE and mPEG_114_‐*b*‐PFarGE copolymers on the phase behavior of this sustainable microemulsion system is expected to be qualitatively comparable to the trends observed in H_2_O/NaCl/*n*‐decane/C_10_E_4_.

While this study focused on the use of a pure C_8_G_1_ surfactant to demonstrate the boosting effect of mPEG_114_‐*b*‐PHHFarGE_5_ also in bio‐based microemulsions, previous studies with microemulsions stabilized by technical‐grade sugar surfactants (Plantacare) have shown the same general properties, offering an easy substitution of the pure C_8_G_1_, with longer alkyl chain surfactants providing more efficient solubilization.^[^ ^87^, ^88^ ^]^ An interesting option would be using recently synthesized farnesene‐based sugar surfactants^[^ ^16^ ^]^ to see how these sterically more demanding surfactants would behave alongside our block copolymers.

### 3.8 Small‐Angle Neutron Scattering of Conventional Microemulsions

SANS is a powerful method to investigate microemulsion nanostructure. Bulk contrast scattering curves were recorded with different amounts of mPEG_114_‐*b*‐PHHFarGE_5_ in the conventional (H_2_O/NaCl – *n*‐decane – C_10_E_4_) as well as the bio‐based (H_2_O – IPM – C_8_G_1_ – farnesol) microemulsion systems to understand the influence of the novel amphiphilic block copolymers on the structural order of the microemulsions and the bending rigidity of the amphiphilic film. For all specimens, H_2_O was replaced by D_2_O in order to maximize the scattering intensity by increasing the scattering length density difference. Given that the use of D_2_O typically results in a minor shift of the phase boundaries, phase behavior studies were performed in advance to ensure the presence of a one‐phase region and to keep proximity to the respective phase inversion. Additionally, due to the higher density of D_2_O, all amphiphile mass fractions (γ) were converted to volume fractions ($\phi_{C+P}$). Equal volumes of water and oil were again used (ϕ = 0.5). Before moving onto the actual scattering curves, the fitting functions used for the analysis of the bulk contrast scattering data are briefly outlined.

**Figure 8:**
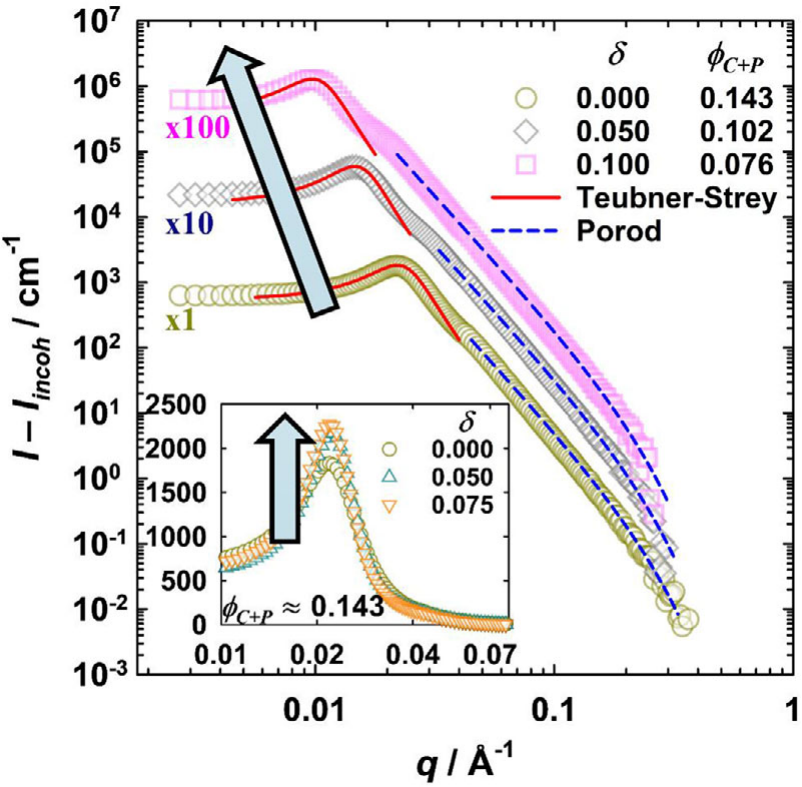
Background‐corrected bulk contrast SANS curves for the microemulsion system D_2_O/NaCl – *n*‐decane – C_10_E_4_/mPEG_114_‐*b*‐PHHFarGE_5_, recorded at different mass fractions δ of copolymer in the mixture of surfactant and copolymer. Peak region analyzed with the Teubner‐Strey model^[^ ^95^ ^]^ (red solid lines); high *q* data analyzed with Porod's law, considering the diffusivity of the interface^[^ ^91^, ^92^ ^]^ (blue dashed lines). Main: In proximity to the respective $\tilde{X}$ point. Curves are displaced by a factor of 10. The arrow indicates the shift to lower *q* with increasing δ. Inset: At a constant amphiphile volume fraction ($\phi_{C+P}\approx$ 0.143, near $\tilde{X}$ of the polymer‐free system). Curves are not displaced. Fits are omitted for clarity. The arrow indicates the increasing scattering peak sharpness with increasing δ.

Figure 8 shows the scattering data of the system D_2_O/NaCl – *n*‐decane – C_10_E_4_/mPEG_114_‐*b*‐PHHFarGE_5_, recorded at different amphiphile volume fractions and copolymer‐to‐amphiphile ratios, but always close to the respective optimum point $\tilde{X}$. All scattering curves reveal several typical characteristics expected for bicontinuous microemulsions. In the low *q* region, the scattering intensity remains constant before a pronounced correlation peak occurs at *q* = *q*_max_. Toward higher *q*, the intensity decreases and characteristically follows a q ^−4^ decay, which is indicative of an internal interface formed by the amphiphilic monolayer separating oil and water subdomains and originates from the strong change of the scattering length density when crossing the nanoscopic interface.^[^ ^90^, ^91^, ^92^ ^]^ An even stronger decay toward the highest experimental *q* is explained by the diffusivity of the interface.^[^ ^92^ ^]^ Multiple scattering contributions explain the occurrence of a shoulder at *q* ≈ 2*q*_max_.^[^ ^32^, ^93^, ^94^ ^]^

Analysis of the peak region of all scattering curves was done with the Teubner‐Strey model,^[^ ^95^ ^]^ describing the absolute scattering intensity I(q) according to

(2) $$I\left(q\right)=\frac{I_{0}}{\left(1-\frac{I_{0}}{I_{max}}\right){\left(\frac{q^{2}}{q_{max}^{2}}-1\right)}^{2}+\frac{I_{0}}{I_{max}}}$$

$I_{0}$ is the scattering intensity toward q→ 0, while $I_{max}$ is the scattering intensity at $q=q_{max}$. Note that the incoherent background scattering has been subtracted from the recorded SANS data. Further details are provided in the Supporting Information. In the intermediate and high q range, the Teubner‐Strey model (red solid lines) typically is qualitatively suitable to describe the q ^−4^ decay, but leads to quantitative deviations from the anticipated trend due to multiple scattering contributions (cf. the shoulder at $q\approx 2q_{max}$).^[^ ^32^, ^93^, ^94^ ^]^

Describing the entire *q* range is possible for instance via a fractal scattering‐based Beaucage model^[^ ^96^ ^]^ or the clipped random wave model,^[^ ^97^ ^]^ both of which introduce a third length scale (radius of gyration and surface roughness parameter, respectively), or by trying to consider multiple scattering by simply combining multiple Teubner‐Strey fits;^[^ ^32^ ^]^ however, given that the Teubner‐Strey model in particular targets the scattering peak, we refrain from using these models. Instead, we analyze the high q data with Porod's law, taking into account the diffusivity of the amphiphilic film,^[^ ^90^, ^91^, ^92^ ^]^ via

(3) $$I\left(q\right)-I_{incoh}=\frac{S}{V}\frac{Q}{\pi \phi_{A}\phi_{B}}q^{-4}\exp\left(-q^{2}t^{2}\right),\;$$

considering the volume fractions of the two domains ($\phi_{A}$ and $\phi_{B}$), the diffusivity of the interface *t* ^[^ ^92^ ^]^ and minimizing the uncertainties in the absolute scattering intensity by means of the so‐called invariant *Q*.^[^ ^91^ ^]^ The incoherent background scattering is subtracted from the absolute intensities to provide a precise analysis of the high q data, allowing for the determination of the specific internal interface *S*/*V*.

When comparing the scattering curves recorded at different δ in proximity to the respective $\tilde{X}$ point, a strong shift of the scattering peak toward lower *q* is seen, indicating an increasing periodicity $d_{TS}$ and thus increasing the size of the oil and water domains in the sponge‐like bicontinuous microemulsion. $d_{TS}$ increases from $d_{TS}$ = (273 ± 6) Å at a surfactant plus copolymer volume fraction of $\phi_{C+P}$ = 0.143 (green circles) to $d_{TS}$ = (618 ± 12)  Å at $\phi_{C+P}$ = 0.076 (pink squares), with the intermediate $\phi_{C+P}$ (gray diamonds) resulting in an intermediate periodicity as anticipated. The increasing $d_{TS}$ can be readily understood given that the boosting effect results in less amphiphile being required to form the monolayer, thereby increasing the size of oil and water subdomains. At the same time, the analysis of the Porod region at higher *q* demonstrates a systematic decrease of the specific internal interface *S*/*V* by roughly a factor of 2 from (0.017 ± 0.002) Å^−1^ to (0.008 ± 0.001) Å^−1^. Considering the multiple scattering contributions evident from the scattering profiles, their influence on the absolute values of *S*/*V* must be kept in mind. All results are compiled in Table 3.

**Table 3:** SANS samples (mass fraction *δ* of mPEG_114_‐*b*‐PHHFarGE_5_ in the mixture of C_10_E_4_ and mPEG_114_‐*b*‐PHHFarGE_5_ and volume fraction $\phi_{C+P}$ of mPEG_114_‐*b*‐PHHFarGE_5_ and C_10_E_4_ in the overall sample) for the investigated polymer‐doped microemulsion systems with the two length scales, periodicity $d_{TS}$ and correlation length $\xi_{\mathrm{TS}}$, obtained via the Teubner‐Strey model,^[^ ^95^ ^]^ as well as the amphiphilicity factor $f_{a}$ and the effective bending rigidity $\kappa_{eff}$. Specific internal interface *S*/*V* and diffusivity of the amphiphilic film *t* determined via Porod's law.^[^ ^91^, ^92^, ^105^ ^]^ Relative errors are $\Delta d_{TS}/d_{TS}$ = 0.02, $\Delta \xi_{TS}/\xi_{TS}$ = 0.03, $\Delta f_{a}/f_{a}$ = 0.015, $\Delta \kappa_{eff}/\kappa_{eff}$ = 0.035, Δ(S/V)/(S/V) = 0.1, Δt/t = 0.1.

δ	$\phi_{C+P}$	*T* ^[^ ^a^ ^]^ / °C	$d_{TS}$ / Å	$\xi_{TS}$ / Å	$f_{a}$	$\kappa_{eff}$ / $k_{B}T$	S/V/Å^−1^	*t* / Å
D_2_O/NaCl – *n*‐decane – C_10_E_4_/mPEG_114_‐*b*‐PHHFarGE_5_								
0.000	0.143	28.7	273	147	−0.84	0.46	0.017	4.0
0.050	0.102	29.4	413	235	−0.85	0.48	0.011	4.2
0.100	0.076	30.6	618	331	−0.84	0.46	0.008	4.5
0.050	0.143	29.4	276	193	−0.90	0.59	0.017	4.5
0.075	0.144	30.6	280	201	−0.91	0.61	0.017	4.5
D_2_O – IPM – C_8_G_1_/mPEG_114_‐*b*‐PHHFarGE_5_ – farnesol								
0.000	0.164 (C_8_G_1_/Polymer)^[^ ^b^ ^]^	25.0	167	51	−0.57	0.26	0.031	3.7
0.050	0.121 (C_8_G_1_/Polymer)^[^ ^b^ ^]^	25.0	267	90	−0.64	0.29	0.019	3.1

^[a]^SANS measurement temperature was chosen close to the phase inversion temperature of the deuterated microemulsion.

^[b]^ $\phi_{Farnesol}$ is 0.15 in both cases.

In a second set of experiments, the influence of copolymer on the microemulsion's structure is studied at constant amphiphile volume fraction $\phi_{C+P}$. As it becomes visible in the inset of Figure 8, increasing the polymer content *d* in the interfacial film leads to a sharper peak while the peak position itself is unaffected. Note however, that the intensity scale is linear, not logarithmic. Given that *q*_max_ strongly relates to the domain size, it is anticipated that the periodicity $d_{TS}$ remains roughly constant (cf. Table 3). In contrast, the increasing peak sharpness suggests an increasing correlation length $\xi_{TS}$, indicative of an increasing order of the bicontinuous structure. Indeed, the value of $\xi_{TS}$ increases from (147 ± 5) Å to (201 ± 6) Å when 7.5 wt% of surfactant are replaced by mPEG_114_‐*b*‐PHHFarGE_5_. Within uncertainty, the Porod analysis does not reveal any change in the specific internal interface *S*/*V*, with *S*/*V* = (0.017 ± 0.002) Å^−1^. Note that, as evident from Figure 5, top, the lamellar phase interferes at higher polymer concentrations if the amphiphile content remains unchanged, which is why higher δ were not accessible for the microemulsion systems recorded at $\phi_{C+P}\approx$ 0.143.

**Figure 9:**
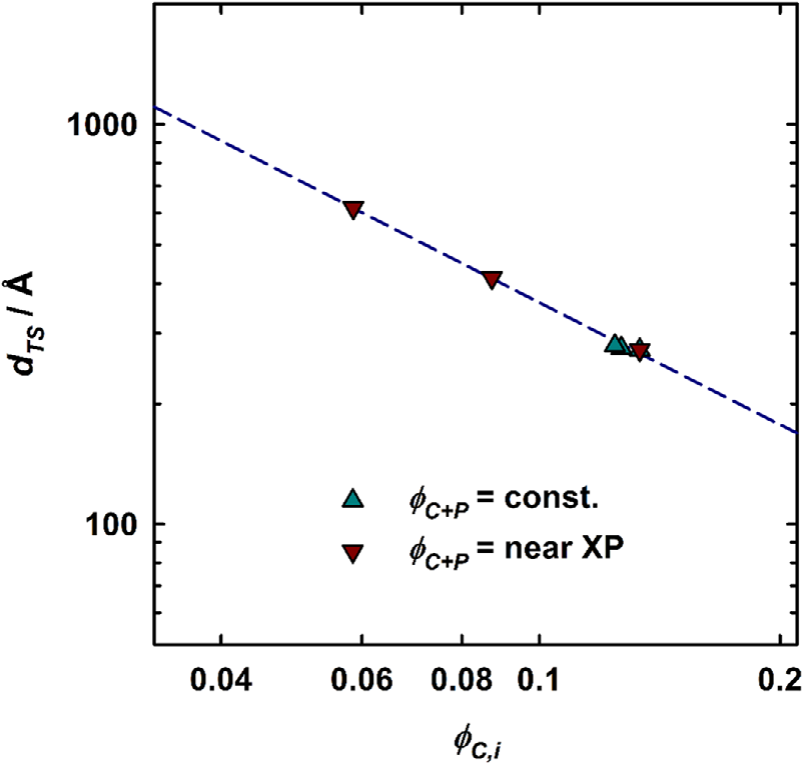
Periodicity *d*_TS_ obtained from the analysis of the SANS curves via the Teubner‐Strey model,^[^ ^95^ ^]^ plotted against the surfactant volume fraction in the interface, $\phi_{C,i}$ for measurements at constant amphiphile volume fraction ($\phi_{C+P}\approx$ 0.143, teal upward triangles) and near the respective $\tilde{X}$ points (brown downward triangles).

Figure 9 shows the periodicity $d_{TS}$ obtained from the analysis of the SANS curves with the Teubner‐Strey model in dependence on the surfactant volume fraction in the interface, $\phi_{C,i}$. Details on the determination of $\phi_{C,i}$ under consideration of the monomeric solubility of the surfactant, mainly in *n*‐decane are provided in the Supporting Information. Given that the characteristic length scale of the domains is proportional to $d_{TS}$ and inversely proportional to the interfacial surfactant volume fraction,^[^ ^98^ ^]^ one would anticipate a linear trend with a slope of –1 in a double‐logarithmic plot. In excellent agreement with this straightforward reasoning, the periodicities $d_{TS}$ obtained from the SANS curves indeed follow a linear trend with a slope of –1.02 ± 0.03. Note that this considers both datasets, that is, the measurements recorded close to the respective $\tilde{X}$ of each polymer‐boosted microemulsion system ($\phi_{C+P}\approx \;\phi_{C+P@\tilde{X}}$ + 0.02, brown downward triangles) and those at a constant amphiphilic volume fraction ($\phi_{C+P}\approx$ 0.143, teal upward triangles).

The length scales $d_{TS}$ and $\xi_{TS}$ determined via SANS can be employed further to determine the bending rigidity of the amphiphilic film in bicontinuous microemulsions, κ. Using Gompper et al.’s modification of the random interface model developed by Pieruschka and Safran,^[^ ^38^, ^99^, ^100^, ^101^ ^]^ the bending rigidity can be calculated via

(4) $$\kappa_{\mathrm{eff}}=\frac{10\pi \sqrt{3}}{64}\frac{\xi_{TS}}{d_{TS}}k_{B}T$$

Note that simulations and experimental studies have indicated that this bending rigidity obtained from the peak analysis of the bicontinuous microemulsion is, in fact a mixture of the actual bending rigidity and the saddle splay modulus $\bar{\kappa }$.^[^ ^102^, ^103^ ^]^ Given that NSE was not available within the scope of this work and an estimation would only be possible using a theoretical value, the determined value in Equation 4 is therefore conceived as an effective bending rigidity, $\kappa_{eff}$, as done in other works.^[^ ^32^, ^41^ ^]^

Theoretically, the influence of thermal membrane fluctuations as well as membrane‐anchored block copolymer is considered in the so‐called renormalized bending rigidity κ_R_ given by ^34^, ^101^, ^102^

(5) $$\kappa_{R}=\kappa_{0}-\frac{3}{4\pi }\ln\left(\frac{l}{a}\right)k_{B}T+\frac{1+\frac{\pi }{2}}{12}\sigma \left(R_{w}^{2}+R_{o}^{2}\right)k_{B}T,$$

where *κ*_0_ is the bare bending rigidity, l/a is the ratio between structural (∼ domain size) and molecular length scale (surfactant length) and $\sigma (R_{w}^{2}+R_{o}^{2})$ quantifies the polymer coverage of the amphiphilic film under consideration of the polymer grafting density σ as well as the end‐to‐end distances of hydrophilic and hydrophobic copolymer blocks in water and oil ($R_{w}$ and $R_{o}$), respectively.

**Figure 10:**
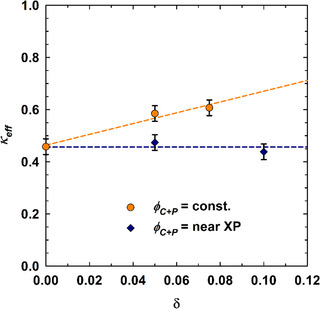
Effective bending rigidity $\kappa_{eff}$ obtained from the analysis of the SANS curves via the Teubner‐Strey model,^[^ ^95^ ^]^ plotted against the mass fraction of copolymer δ in the amphiphilic mixture.

Recall now the two different sets of SANS experiments shown in Figure 8. The obtained bending rigidities are presented in Figure 10 and reveal a clear distinction between the two pathways, that is, when comparing different polymer concentrations δ at fixed amphiphile concentration or at each system's respective $\tilde{X}$ point. Within measurement uncertainty, bending rigidities that were determined near $\tilde{X}$ of each microemulsion system (blue diamonds) do not change as a function of polymer concentration, with $\kappa_{eff,average}$ = (0.47 ± 0.02) $k_{B}T$, indicating a compensation of thermal fluctuations (making the film softer) and polymer adsorption in the interfacial film (making the film more rigid). In contrast, $\kappa_{eff}$ increases if the amphiphile content in the sample remains constant (orange circles). Since the amphiphile fraction remains constant, which in turn leads to a δ‐independent periodicity, the thermal fluctuation term in Equation 5 remains unaffected, and polymer adsorption becomes the sole influence on the bending rigidity. Note that the pre‐factor of this term could not be determined as it requires information on the end‐to‐end distances of hydrophilic and hydrophobic blocks in water and oil, respectively, with the hydrophobic P(HH)FarGE_m_ end‐to‐end distances not being available to us.

In order to quantify the structural order of microemulsions, it has proven useful to determine the so‐called amphiphilicity factor $f_{a}$ (cf. Supporting Information).^[^ ^95^, ^104^ ^]^ On one hand, values of $f_{a}$ > 1 are found for disordered solutions with uncorrelated interfaces. On the other hand, the more negative $f_{a}$, the higher the structural order, with ‐1 corresponding to perfectly aligned lamellar sheets. Well‐structured microemulsions exhibit amphiphilicity factors of $f_{a}$ < –0.6.^[^ ^104^ ^]^ For the microemulsion system D_2_O/NaCl – *n*‐decane – C_10_E_4_/mPEG_114_‐*b*‐PHHFarGE_5_, the first set of experiments measured near the respective $\tilde{X}$ points reveals an almost constant $f_{a}$ = –0.84 ± 0.02, indicating an overall high structural order. From the second set at $\phi_{C+P}$ = const. ≈ 0.143, a decrease from $f_{a}$ = ‐0.84 ± 0.01 to $f_{a}$ = ‐0.91 ± 0.01 is found, indicating a strong increase in structural order caused by the addition of mPEG_114_‐*b*‐PHHFarGE_5_. Both results are in excellent quantitative agreement with the results obtained by Schneider et al. for their mPEG_113_‐*b*‐PAlkGE_m_ block copolymers in the same microemulsion system.^[^ ^32^ ^]^

### 3.9 Small‐Angle Neutron Scattering of Bio‐Based Microemulsions

**Figure 11:**
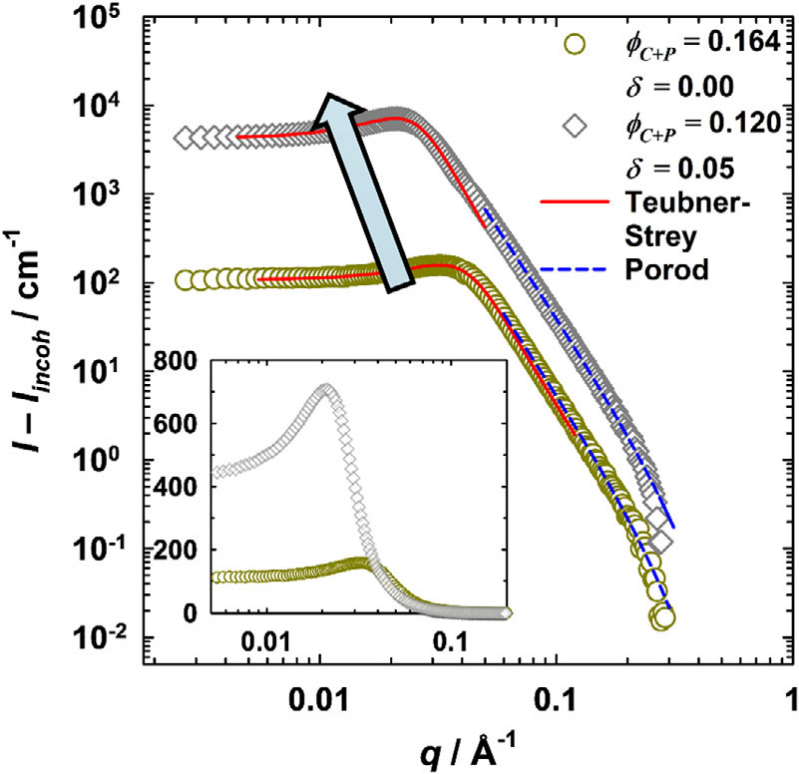
Background‐corrected bulk contrast SANS curves for the microemulsion system D_2_O – isopropyl myristate – C_8_G_1_/mPEG_114_‐*b*‐PHHFarGE_5_ – farnesol, recorded at different mass fractions of copolymer δ in the amphiphilic mixture in proximity to the respective $\tilde{X}$ point at *T *= 25 °C. The farnesol volume fraction is constant with $\phi_{\mathrm{Farnesol}}$ = 0.15. Peak region analyzed with the Teubner‐Strey model^[^ ^95^ ^]^ (red solid lines); high *q* data analyzed with Porod's law, considering the diffusivity of the interface^[^ ^91^, ^92^ ^]^ (blue dashed lines). Main: Log‐log representation. Curves are displaced by a factor of 10. Inset: Lin‐log representation around the peak region. Curves are not displaced.

Moving to the bio‐based microemulsion system D_2_O – isopropyl myristate (IPM) – C_8_G_1_/mPEG_114_‐*b*‐PHHFarGE_5_ – farnesol, it is evident that the scattering curves shown in Figure 11 likewise demonstrate the typical characteristics of bicontinuous microemulsion outlined above. Polymer addition, which shifts the $\tilde{X}$ point to lower amphiphile volume fractions, again leads to a shift toward lower scattering vectors (gray diamonds), in agreement with larger domain sizes, resulting in $d_{TS}$ increasing from $d_{TS}$ = (167 ± 3) Å to $d_{TS}$ = (267 ± 5)  Å. Nonetheless, several differences compared to the H_2_O/NaCl – *n*‐decane – C_10_E_4_ microemulsion system can be observed. First, the multiple scattering is much less pronounced, in particular for the polymer‐free sample, due to the smaller size of the water and oil domains, which scatter significantly less. Correspondingly, the main peak has shifted to higher *q* values compared to the C_10_E_4_ microemulsion because of the overall larger amount of amphiphile. In agreement, the analysis of the high *q* data reveals a specific internal interface *S*/*V* higher than for the C_10_E_4_ microemulsions, even in the presence of polymer, with *S*/*V* being (0.031 ± 0.003) Å^−1^ and (0.019 ± 0.002) Å^−1^, respectively. Second, the peak is not as sharp as for the C_10_E_4_ microemulsion. Thus, the ratio of the length scales $d_{TS}$/$\xi_{TS}>$ 2 deviates noticeably from $d_{TS}$/$\xi_{TS}\approx$ 2 reported by Sottmann et al. for different D_2_O – *n*‐alkane – C*_i_* E*_j_* formulations.^[^ ^98^ ^]^ As a consequence, the structural order of the amphiphilic monolayer is lower in the sustainable microemulsion. Correspondingly, the amphiphilicity factor is less negative with $f_{a}$ = ‐0.57 ± 0.01 for the polymer‐free bio‐based microemulsion. Polymer addition results in a small gain of structural order and the amphiphilicity factor decreases to $f_{a}$ = ‐0.64 ± 0.01 with 5 wt% polymer in the polymer/C_8_G_1_ mixture (δ = 0.05). With respect to $\kappa_{eff}$, the bending rigidity of the amphiphilic monolayer is significantly lower than in the C_10_E_4_ microemulsion, with values ranging from $\kappa_{eff}$ = (0.26 ± 0.01) $k_{B}T$ to $\kappa_{eff}$ = (0.29 ± 0.01)  $k_{B}T$ for the bio‐based microemulsions with δ = 0.00 and δ = 0.05, respectively.

These observations can be attributed to the altered oil and surfactant components in the sustainable formulation when compared to the conventional microemulsion system. It has been shown by Sottmann et al. that the bare bending rigidity ($\kappa_{0}$) increases with an increasingly hydrophobic alcohol ethoxylate surfactant if the alkane remains unchanged.^[^ ^98^ ^]^ Ryan and Kaler have observed lower amphiphilicity factors (i.e., a higher structural order) for more hydrophobic alkyl polyglucosides.^[^ ^106^ ^]^ At the same time, the bending rigidity decreases with increasing oil chain length if an identical alcohol ethoxylate is used.^[^ ^98^ ^]^ IPM has a long alkyl chain and C_8_G_1_ has a short tail length, while *n*‐decane and C_10_E_4_ have a medium alkyl chain. Additionally, further taking into account the sterically demanding cyclic sugar head group of the surfactant and the fact that IPM is a comparatively polar oil, this could then also be reflected in the bare bending rigidity of the *n*‐octyl β‐D‐glucopyranoside (C_8_G_1_) membrane being smaller than that of the C_10_E_4_ membrane. Further experiments using, for instance NSE would be required to confirm this hypothesis.

## 4 Conclusion

Aiming at more economic and ecological strategies for the synthesis and application of surfactants, we present the crown ether‐assisted one‐step AROP of bio‐renewable terpenyl glycidyl ethers (TGEs), namely FarGE and its novel saturated derivative HHFarGE. These amphiphilic diblock copolymers mPEG_114_‐*b*‐P(HH)FarGE_m_ feature molecular weights in the range of 5600–8400 g·mol^−1^ and low dispersities of 1.04 – 1.07. The degree of polymerization of the TGEs represents a simple handle to adjust amphiphilicity. Like conventional surfactants, the diblock copolymers self‐assemble in aqueous solution when exceeding the critical micelle concentration (CMC), which was studied via fluorescence spectroscopy and light scattering, revealing low CMCs (14 – 90 mg·L^−1^) that systematically decrease with TGE block size due to the increasing hydrophobic effect.

The applicability of hydrogenated and nonhydrogenated diblock copolymers as cosurfactants in microemulsions was investigated through the phase behavior of a conventional pseudo‐ternary, nonionic microemulsion system, H_2_O/NaCl – *n*‐decane – C_10_E_4_, by varying the number *m* of TGE repeating units. The observed reduction in the amphiphile amount required for complete water/oil solubilization indicates a strong efficiency boosting effect. Intriguingly, it is not the copolymers with the largest (HH)FarGE block that led to the largest gain in solubilization performance, but rather those with a medium block size in the range of *m* = 5–6. We thus hypothesize that the (HH)FarGE block, unlike the PEG block in the H_2_O domains, does not form a mushroom conformation in the *n*‐decane domains, but instead is located rather in and close to the amphiphilic film and only slightly extends into the *n*‐decane domains due to the FarGE side chains. No significant effect by virtue of hydrogenation is observable, despite the slight increase in side chain flexibility due to the removal of double bonds in FarGE, signaling that the boosting effect is predominantly related to the number of TGE units. Given the bio‐renewable nature of the block copolymers, their suitability as additives in bio‐based formulations was successfully demonstrated, utilizing the diblock copolymer mPEG_114_‐*b*‐PHHFarGE_5_ as a highly effective efficiency booster in a H_2_O – isopropyl myristate – C_8_G_1_ – farnesol microemulsion.

We further determined the length scales of both microemulsion systems doped with mPEG_114_‐*b*‐PHHFarGE_5_ via SANS, observing a higher structural ordering upon polymer addition, quantified by means of the amphiphilicity factor. At the same time, the bending rigidity increases with a higher polymer coverage of the amphiphilic film. We confirmed a lower structural order of the sustainable microemulsion system, which was attributed to the weakened solubilization of the sterically demanding isopropyl myristate by C_8_G_1_ and farnesol.

The strong boosting potential of the novel TGEs in bio‐based microemulsions serves as an excellent foundation for future formulations targeting rhamnolipids^[^ ^107^ ^]^ or hexahydrofarnesyl‐based sugar surfactants^[^ ^16^ ^]^ and provides avenues to enhance the performance and sustainability of microemulsions, valorizing their potential in various biomedical applications.

## Supporting Information

General information for reagents, instrumentation, and experimental procedure for the monomer synthesis, as well as for aqueous, microemulsion, and SANS studies; additional characterization data of the synthesized monomers and polymers (NMR spectroscopy, SEC, and DSC analysis); additional figures on copolymer aqueous self‐assembly and microemulsion phase behavior (fluorescence spectroscopy, light scattering, phase diagrams).

## Conflict of Interest

The authors declare no competing financial interest.

## Supporting information



Supporting Information

## Data Availability

Data openly available in a public repository that issues datasets with DOIs
