# Supplementary material for: Bio‐Based Amphiphilic Farnesyl Glycidyl Ether Block Copolymers: Aqueous Self‐Assembly and Solubilization Boosting
Source: Chemistry. 2025 Nov 17;31(72):e02614. doi: 10.1002/chem.202502614 (PMC12731559; doi:10.1002/chem.202502614)
Supplement: Supplementary file 1 — Supporting Information [file CHEM-31-e02614-s001.docx]

**Supporting Information**

Bio-Based Amphiphilic Farnesyl Glycidyl Ether Block Copolymers: Aqueous Self-Assembly and Solubilization Enhancement

Maximilian Krappel^1^, Sandra Schüttner^2^, Ingo Schneider^1^, Pascal Schiffmann^2^, Ralf Schweins^3^, Holger Frey^2^_,_ Thomas Sottmann^1,*^

^1^Institute of Physical Chemistry, University of Stuttgart, Pfaffenwaldring 55, 70569 Stuttgart, Germany

^2^Department of Chemistry, Johannes Gutenberg University Mainz, Duesbergweg 10-14, 55128 Mainz, Germany

^3^Institut Laue-Langevin, DS / LSS, 71 Avenue des Martyrs, CS 20 156, 38042 Grenoble CEDEX 9, France

E-Mail: thomas.sottmann@ipc.uni-stuttgart.de

Table of Contents

[1. Experimental Section 2](#_Toc205728107)

[2. Monomer Synthesis 9](#_Toc205728108)

[3. Polymerization Procedures 13](#_Toc205728109)

[4. NMR and IR Spectra of HHF and HHFarGE 17](#_Toc205728110)

[5. NMR Characterization of Diblock Copolymers of HHFarGE and mPEG_114_ 22](#_Toc205728111)

[6. NMR Characterization of PHHFarGE_m_ 25](#_Toc205728112)

[7. Characterization of PHHFarGE_m_ and PFarGE_m_ Homopolymers 28](#_Toc205728113)

[8. SEC Measurements of Diblock Copolymers of FarGE and mPEG_114_ 30](#_Toc205728114)

[9. DSC Thermograms of the Synthesized Diblock Copolymers and Homopolymers 31](#_Toc205728115)

[10. Determination of CMCs via Fluorescence Spectroscopy (FS) 33](#_Toc205728116)

[11. Determination of CMCs via Static and Dynamic Light Scattering (SLS/DLS) 37](#_Toc205728117)

[12. Phase Behavior of H_2_O/NaCl – *n*-decane – C_10_E_4_ / mPEG_114_-*b*-PFarGE_5_ 39](#_Toc205728118)

[13. References 40](#_Toc205728119)

1. Experimental Section

Reagents

**Reagents for Polymer Synthesis.**

Solvents and chemicals were generally acquired from commercial suppliers (Sigma-Aldrich, Fluka, Alfa Aesar, Acros, TCI) and were used without prior purification, unless stated otherwise. Deuterated solvents were purchased from Deutero GmbH. Farnesol was obtained as an isomeric mixture from Aldrich (95% purity) and ethylene oxide (EO) from Air Liquide. All terpenyl glycidyl ethers (TGEs), namely hexahydrofarnesyl glycidyl ether (HHFarGE) and farnesyl glycidyl ethers (FarGE), were dried azeotropically with benzene under reduced pressure overnight prior to polymerization. For homopolymer synthesis, the TGEs were dried over calcium hydride (CaH_2_) and vacuum transferred before polymerization. Poly (ethylene glycol) monomethyl ether (mPEG_114_), received from Fluka, was applied as a macroinitiator for the diblock copolymer synthesis and the following data was revealed by ^1^H NMR and SEC analysis: *M*_n, NMR_: 5050 g·mol^-1^, degree of polymerization (*P*_n_) = 114, *Đ* = 1.05. Dialysis membranes (regenerated cellulose, *MWCO* = 1,000 gmol^‑1^) were purchased from Orange Scientific.

**Reagents for Microemulsion & Small-Angle Neutron Scattering Studies.**

Water was double-distilled before usage. For SANS experiments, it was replaced by D_2_O (Deutero, 99.9 %). NaCl was purchased from Carl Roth (99.5 %). *n*-Decane was acquired from Thermo Scientific (99 %), isopropyl myristate from TCI Chemicals (98 %) The surfactants tetraethylene glycol monodecyl ether (C_10_E_4_) and *n*-octyl β-D-glucopyranoside (C_8_G_1_) were purchased from Bachem (99 %) and Glycon (99.5 %), respectively. Farnesol was obtained from Thermo Scientific (96 %, isomeric mixture). All chemicals used were used without further purification.

Instrumentation

**Nuclear Magnetic Resonance (NMR) Spectroscopy.**

^1^H (400 MHz), ^13^C (100 MHz), ^1^H-^1^H-COSY, ^1^H-^13^C HSQC and ^1^H-^13^C HMBC NMR spectra were recorded on a Bruker Avance II HD 400 spectrometer (equipped with a 5 mm BBFO-Smartprobe (Z-gradient), an automated tuning and matching device (ATM) as well as a SampleXPress 60 auto sampler) at 23 °C. All spectra were referenced internally to residual proton signals of the deuterated solvent (^1^H NMR spectra) and the deuterated solvent itself (^13^C NMR spectra). NMR spectroscopy data is reported as follows: chemical shift, multiplicity and integration. Spectra annotation uses lowercase letters for proton signals and capital letters for carbon signals.

**Size Exclusion Chromatography (SEC).**

SEC was performed in dimethylformamide (DMF) with 1 g·mL^-1^ lithium bromide as an eluent (flow rate of 1mL·min^-1^, at 50 °C) on an Agilent 1100 series SEC system with a HEMA 300/100/40 Å column cascade, equipped with UV (254 nm) and RI detectors. The DMF SEC calibration was carried out with poly(ethylene glycol) standards provided by Polymer Standard Service (PSS). Additional SEC measurements of the homopolymers were conducted in THF (flow rate of 1mL·min^-1^) using an instrument consisting of a Waters 717 plus autosampler, a set of three MZ-Gel SDPlus columns based on SDV with particle sizes of 5 μm (porosities of 100, 10^3^ and 10^5^ Å) and a TSP Spectra P 100 pump. An Agilent Technologies 1260 Infinity RI-detector as well as a SpectraSYSTEM UV2000 UV-detector (254 nm) were used. Polystyrene standards provided by PSS were used for calibration and toluene was applied as an internal standard. All data recording and analysis was carried out using PSS WinGPC Unity.

**Fourier-Transformations-Infrared (FT-IR) Spectroscopy.**

FT-IR spectroscopy was conducted using a Nicolet iS10 FT-IR spectrometer (Thermo Scientific, Waltham, MA, USA) equipped with a diamond ATR unit in a frequency range of 650–3500 cm^-1^.

**Differential Scanning Calorimetry (DSC).**

DSC for thermal analyses of the synthesized polymers was performed on a DSC 250, TA Instrument, utilizing indium and *n*-octane as calibration standard. All samples were dried azeotropically, hermetically sealed in an aluminium pan and measured against an empty pan as reference under a nitrogen atmosphere. The DSC analysis temperature protocol ranges from -90 °C to 100 °C and consists of two cooling and heating cycles at a rate of 10 °C·min^‑1^ to remove the thermal history. For the diblock copolymer analysis, a 5 min hold after each cycle was added. The temperature for the homopolymer analysis was set to -90 °C to 50 °C. All analysis data were obtained from the second heating cycle using the TRIOS software.

**Fluorescence Spectroscopy (FS) for CMC determination.**

The determination of the CMC was realized fluorometrically using pyrene as a hydrophobic fluorescence dye and has been widely established.^1–4^ Two polymer stock solutions (concentration range of 0.10 – 1 g·L^‑1^) per diblock copolymer were prepared using Milli-Q water and equilibrated overnight. For each measurement, a defined aliquot of an ethanolic pyrene solution (0.7 mM) was added to a volumetric flask, followed by solvent evaporation. Then, a calculated amount of the polymeric stock solution was added to the volumetric flask and diluted with distilled water (Milli-Q) to obtain a final pyrene concentration (*c*_pyrene_) of 7·10^-7^ M. For each polymer, a concentration series of pyrene-containing polymer solutions (0.0005 to 0.60 g·L^-1^) were measured. The samples were gently shaken at room temperature (r.t.) overnight to enable micelle equilibration and solubilization of pyrene by the micelles. Pyrene emission spectra were recorded on a Jasco FP-8200 spectrofluorometer through a quartz cell at *T* = (23 $\pm$ 1) °C in a wavelength range of 350-450 nm. Quartz cells were washed once with the corresponding polymer solution prior to the measurement. Selected instrument parameters were as follows: excitation wavelength λ_ex_ = 334 nm, slit_em_ = 2.5 nm, slit_ex_ = 2.5 nm, scan speed: 20 nm·min^-1^, data interval = 0.5 nm, response = 0.5 sec. The intensity ratio *I*_1_/*I*_3_, referring to the first (λ_peak_ = 372-373 nm) and third (λ_peak_ = 383-384 nm) vibronic bands in the pyrene fluorescence spectrum,^4,5^ is extremely sensitive to the polarity of the medium surrounding pyrene molecules. In an aqueous solution of amphiphilic poylmers, a ratio change of *I*_1_/*I*_3_ hence reflects the pyrene location in the polar aqueous solution or a non-polar micellar core.^1,6^ Plotting *I*_1_/*I*_3_ against polymer concentration is a convenient approach to estimate the CMC of the respective polymer.

**Static and Dynamic Light Scattering (SLS/DLS).**

Light scattering experiments were performed with a 3D LS Spectrometer (LS Instruments, Switzerland) equipped with a diode-pumped laser operating at a wavelength of $\lambda$ = 561 nm (Cobolt, Sweden). A combination of dynamic (DLS) and static light scattering (SLS) was conducted at a scattering angle of $\theta$ = 90° and a maximum incident laser intensity of $I_{0}$ $\approx$ 82 mW. A decalin bath connected to a thermostat (Julabo, Germany) was used to maintain a temperature of *T* = (23.0 $\pm$ 0.1) °C. The block copolymer concentrations were in the range between 0.003 g·L^-1^ and 1 g·L^‑1^ and solutions were prepared in 10 mL volumetric flasks. For each sample concentration, an aliquot of 1 mL of the polymer solution was filled into a borosilicate test tube (Fisher Scientific, USA), which were previously cleaned with acetone and filtered double-distilled water. As measurement setup, 60 repetitions of 5 s each was chosen for all polymer concentrations. The 2D Pseudo Cross Correlation mode was applied to avoid errors in the correlation function at short lag times, which is caused by after-pulsing of the detectors. The static light scattering intensity was evaluated using the mean count rate ($meanCR$ in kHz). $meanCR$ is derived from the geometric mean of the count rates recorded at the two detectors (${CR}_{A}$ and ${CR}_{B}$), following $meanCR= \sqrt{{CR}_{A}*{CR}_{B}}*\sin\left( \theta\right)\equiv C$. All values were further corrected by considering the scattering intensity of a polymer-free water sample (see below). The conversion of count rates to absolute units (1/cm) was possible by using the scattering intensity of a known scatterer, toluene. The reference Rayleigh ratio for our setup was obtained according to a procedure by Wu et al..^7^ The Rayleigh ratio with unpolarized incident light and detection without a polarizer for toluene follows $R_{u,25}$, ($\theta$ = 90°, *T = 25 °C*)

| $R_{u,25}=4.9*{10}^{6}*\lambda^{-4.17}$ $\left[ \frac{1}{cm} \right]$ | (1) |
| --- | --- |

Vertically polarized incident light was applied while a vertical polarizer detected the signal. Hence, we convert $R_{u}$ to $R_{vv}$ via the depolarization factor $\rho_{u}$ ($\rho_{u}$ = 0.491 at *T* = 23 °C)^8^ and the relation:

| $R_{vv}=R_{u}(2-\rho_{u})/(1+\rho_{u}) \left[ \frac{1}{\mathrm{cm}} \right]$. | (2) |
| --- | --- |

As the temperature dependence of the Rayleigh ratio of toluene is similar to that of benzene, it is incorporated via

| $R_{vv,T}=R_{vv,25}\left( 1+0.00368\left( T-25 ^{\circ}C \right) \right) \left[ \frac{1}{\mathrm{cm}} \right].$ | (3) |
| --- | --- |

The excess Rayleigh ratio is determined from

| $R_{vv,T,sample}={\left( \frac{C_{sample}}{I_{0,sample}}-\frac{C_{water}}{I_{0,water}} \right)*\left( \frac{I_{0,toluene}}{C_{toluene}} \right)R}_{vv,T,toluene},$ | (4) |
| --- | --- |

with $I_{0,x}$ labelling the incident laser beam intensity used for measurements of water, toluene, and the sample. The setup conditions (λ = 561 nm, *T* = 23 °C, $\theta$ = 90°) lead to $R_{vv,T,toluene}=1.694*{10}^{-5}$ $\frac{1}{\mathrm{cm}}$.

**Phase Behavior Studies.**

Phase behavior of pseudo-ternary H_2_O/NaCl – *n*-decane – tetraethylene glycol monodecyl ether (C_10_E_4_) / mPEG_114_-*b*-P(HH)FarGE_m_ systems was studied in the form of *T*(γ) cuts, also known as fish cuts. For all measurements, a constant salinity (0.1 wt.% of NaCl in water to buffer electrostatic interactions with ionic surfactant impurities) and a constant water-to-oil ratio $\phi$ was set, using equal volumes of water and oil, corresponding to

$\phi=\frac{V_{o}}{V_{w}+V_{o}} = 0.5$.

Temperature *T*, amphiphile mass fraction $\gamma$ and polymer content in the amphiphilic mixture $\delta$ were varied, with

$$\gamma=\frac{m_{Surfactant+Polymer}}{m_{Sample}}$$

and

$$\delta=\frac{m_{Polymer}}{m_{Surfactant+Polymer}}.$$

Components were filled into 5 or 10 mL test tubes in the order polymer > surfactant > oil > brine. A stirring bar was inserted afterwards and the test tube was sealed. Temperature of the water bath was regulated via a thermostat (Thermo Scientific Haake SC 100 and Thermo Scientific Haake A 25) and read off a thermometer with an accuracy of ± 0.01 K (Greisinger GTF 401 and Greisinger GMH 3750). A magnetic stirrer (Heidolph MR Hei-Mix L) with a speed range of 0 to 1400 rpm was used to ensure mixing of the samples. Two-phase regions could be determined from sample turbidity and after awaiting phase separation. The presence of anisotropic phases can be detected using vertically oriented polarizing filters. After successful determination of all phase boundaries, the sample was diluted by adding equal volumes of water and oil, therefore obtaining lower γ.

Phase behavior of pseudo-quaternary H_2_O/NaCl – isopropyl myristate – *n*-octyl β-D-glucopyranoside (C_8_G_1_) / mPEG_114_-*b*-P(HH)FarGE_5_ – farnesol systems was studied in an isothermal fish cut (*T* = 25°C) through the phase tetrahedron. In contrast to the first measurement, samples were prepared at a given composition (i.e., denoted by C_8_G_1_/polymer and farnesol mass fraction, see below), after which farnesol was added dropwise. The system was left to equilibrate after each droplet to detect the number and appearance of phases. Further droplets were added until all phase boundaries were determined. Afterwards, a fresh sample was prepared at a lower C_8_G_1_/polymer content; the process was then repeated to complete the fish. As for the systems before, a constant water-to-oil ratio of $\phi$ = 0.5 was set. The polymer content in the polymer/surfactant mixture is defined as above. Note that the parameter $\gamma$ is now represented as $w_{CP}$, with

$$w_{CP}=\frac{m_{Surfactant+Polymer}}{m_{Sample}},$$

which is formally identical to $\gamma$ but measurement-wise different because (1) it keeps changing when the alcohol (farnesol) is added to sample (since $m_{Sample}$ increases continually) and (2) farnesol represents a separate edge of the phase tetrahedron, leading to another parameter,

$$w_{D}=\frac{m_{Farnesol}}{m_{Sample}}.$$

**Small-Angle Neutron Scattering.**

Samples for small-angle neutron scattering (SANS) experiments were prepared with D_2_O instead of H_2_O, making use of the vastly different scattering length densities of hydrogen and deuterium in order to adjust the so-called bulk contrast. Note that the density of D_2_O is significantly higher than that of H_2_O. Mass fraction notation used for the non-deuterated microemulsion systems (parameters $\gamma$, $w_{C}$, $w_{D}$) was therefore adjusted to volume fractions, with

$$\phi_{CP}=\frac{V_{Surfactant+Polymer}}{V_{Sample}}$$

and (for the pseudo-quaternary system)

$$\phi_{Farnesol}=\frac{V_{Farnesol}}{V_{Sample}}$$

Phase behavior of the investigated microemulsion systems was checked before each experiment. The sample tube was then placed inside a water bath set to the target temperature (in the one-phase region) before transferring it into a Hellma quartz cell (path length 1 mm), which was also kept at the target temperature. The cells were placed inside a thermo-controlled sample holder pre-heated to the measurement temperature, which was then manually shaken to ensure a homogeneous sample during the measurement. It was always checked that specimen showed no signs of demixing/phase separation.

All experiments were performed at the D22 spectrometer at the Institut Laue-Langevin (Grenoble, France), using a neutron wavelength of $\lambda$ = 6 Å with a wavelength spread of $\Delta\lambda/\lambda$ = 10 %. A detector/collimation distance of 17.6 m/17.6 m was set and an additional side detector placed at 1.4 m was used to cover a $q$ range from roughly 0.003 Å^-1^ to 0.5 Å^-1^, with the scattering vector $q$ defined as

$$q=\frac{4\pi}{\lambda}\cdot\sin\left( \frac{\theta}{2} \right),$$

with the scattering angle $\theta$. Raw data were treated and analyzed with the Matlab-based GRASP V10.17e software package. Absolute scattering intensities $I(q)$ were obtained from direct beam measurements, taking into account sample transmission as well as empty cell and incoherent background scattering $I_{incoh}$.

The model of Teubner and Strey is based on an order parameter expansion of the free energy density using Landau theory,^9,10^ from which the parameters $a_{2}$, $c_{1}$, $c_{2}$ are incorporated into the scattering intensity via

$$I\left( q \right)\propto\frac{1}{a_{2}+c_{1}q^{2}+c_{2}q^{4}}.$$

Using more descriptive parameters, the fitting function presented in the main manuscript is obtained,

$$I\left( q \right)= \frac{I_{0}}{\left( 1-\frac{I_{0}}{I_{max}} \right)\left( \frac{q^{2}}{q_{max}^{2}}-1 \right)^{2}+\frac{I_{0}}{I_{max}}}.$$

The parameters $a_{2}$, $c_{1}$, $c_{2}$ can be related to the Teubner-Strey fitting parameters via

$$a_{2}=I_{0}^{-1}$$

$$c_{1}=-2q_{max}^{2}c_{2}$$

$$c_{2}=q_{max}^{-4}(I_{0}^{-1}-I_{max}^{-1})$$

From this, it is possible to calculate the length scales, namely the periodicity $d_{TS}$, which quantifies the size of the oil and water nanodomains in the sponge-like bicontinuous microemulsion, as well as the correlation length $\xi_{TS}$, which is a characteristic length for positional correlation and order.^9,11^ Here,

$$d_{TS}=2\pi\left[ \frac{1}{2}\left( \frac{a_{2}}{c_{2}} \right)^{0.5}-\frac{c_{1}}{{4c}_{2}} \right]^{-0.5},$$

$$\xi_{TS}=\left[ \frac{1}{2}\left( \frac{a_{2}}{c_{2}} \right)^{0.5}+\frac{c_{1}}{{4c}_{2}} \right]^{-0.5}.$$

The structural order of microemulsions can be quantified via the amphiphilicity factor

$$f_{a}=\frac{c_{1}}{\left( 4a_{2}c_{2} \right)^{0.5}},$$

which can range from above +1 if the structure is completely disordered down to -1 for a perfectly aligned lamellar phase, with the typical peak in the scattering pattern occurring when the amphiphilicity factor decreases below $f_{a}=0$, which is when the so-called Lifshitz line is crossed.^10,12^

For the scaling of the SANS length scales, the surfactant volume fraction in the interface, $\phi_{C,i}$, is required. Under the assumption that the monomeric solubility of C_10_E_4_ in water ^13,14^ as well as that of the block copolymer in both water and oil are negligible, its value can be calculated via

$$\phi_{C,i}=\phi_{C}-\phi_{mon,B}\phi_{B},$$

where $\phi_{C}$ and $\phi_{B}$ are surfactant and oil volume fraction, respectively, and $\phi_{mon,B}$ is the monomeric solubility of C_10_E_4_ in *n*-decane.^13^

1. Monomer Synthesis

**Hydrogenation of Farnesol.**

**
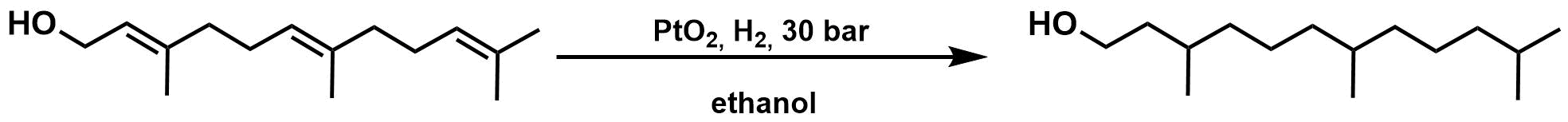
**

Scheme S 1: Hydrogenation of farnesol utilizing PtO_2_ under 30 bar H_2_, yielding hexahydrofarnesol_._

To synthesize hexahydrofarnesol (HHF, IUPAC: 3,7,11-trimethyldodecan-1-ol), we altered a literature-known hydrogenation procedure deploying platinum (IV)-oxide (PtO_2_) as catalyst.^15^ Farnesol (IUPCAC: 3,7,11-Trimethyl-2,6,10-dodecatrien-1-ol; 15.00  g, 16.85 mL, 0.068 mol, 1.00 equiv.), dis­solved in 6.75 mL ethanol, and PtO_2_ (0.055 g, 0.241 mmol, 0.0012 mol% per double bond, 0.004 equiv.) were introduced in a high pressure autoclave reactor, equipped with a stirring bar, and the hydrogenation was performed under 30 bar H_2_ at r.t.. The reaction conversion was tracked via ^1^H NMR spectroscopy and quantitative conversion achieved after 30 h. The hydrogenation mixture was filtered and residual ethanol was removed via rotary evaporation to yield HHF as a colorless liquid in quantitative amounts (90 %). NMR characterization reveals a stereoisomeric mixture after hydrogenation and is in agreement with previously reported literature (Figure S1 - Figure S4).^16^


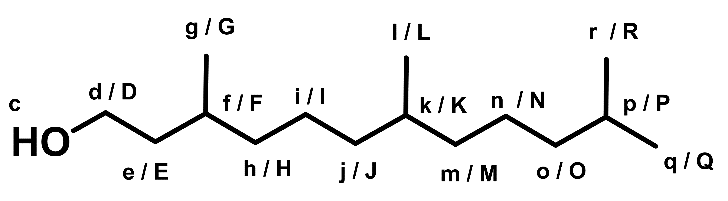


^1^H NMR (400 MHz, chloroform-*d*_1_): δ (ppm) = 3.73 – 3.62 (m, 2H, H_d_), 1.66 – 0.95 (m, 17H, H_e_, H_f_ H_h_, H_i_, H_j_, H_k_, H_m_, H_n_, H_o_, H_p_), 0.91 – 0.80 (m, 12H, H_g_, H_l_, H_r_, H_q_).

^13^C NMR (100 MHz, chloroform-*d*_1_): δ (ppm) = 61.39 (C_D_), 40.16 + 40.08 (d, C_E_), 39.50 (C_O_), 37.63 + 37.59 (d, C_M_), 37.50 (C_H_), 37.46 + 37.40 (d, C_J_), 32.92 (C_K_), 29.66 + 29.64 (d, C_F_), 28.12 (C_P_), 24.95 + 24.93 (C_N_), 24.51 (C_I_), 22.86 + 22.76 (C_Q_ + C_R_), 19.87 (d) + 19.82 + 19.75 (d) (C_G_ + C_L_). d denotes a doublet signal of the carbon resonance due to the stereoisomeric mixture.

**Phase transfer catalysis and methylation of protic impurities in the terpenyl glycidyl ether.**


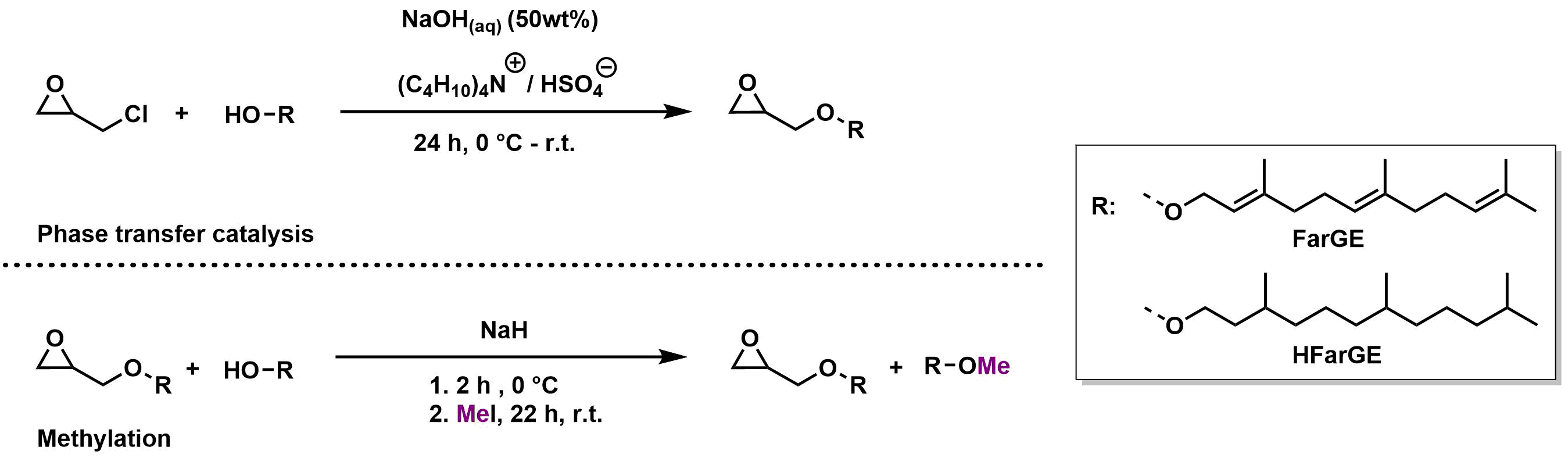


Scheme S 2: Phase transfer catalysis of terpenoids with subsequent methylation of potential protic impurities.

The terpenyl glycidyl ethers (TGEs) such as FarGE and its novel hydrogenated derivative HHFarGE were synthesized in a literature-modified phase transfer catalysis.^17,18^ In the following, the procedure is described exemplarily for HHFarGE. A 250 mL three-necked flask, equipped with a dropping funnel and mechanical stirrer, was charged with epichlorohydrin (ECH, 32.8 mL, 38.65 g, 0.418 mol, 5.30 equiv.), tetrabutylammonium hydrogen sulfate (TBAHS, 1.07 g, 0.003 mol, 0.04 equiv.) and an aqueous sodium hydroxide solution (*V*_solution_ = 40.03 mL, NaOH: 19.93 g, 0.50 mol, 12.7 equiv.). While keeping the reaction mixture to 0 °C with an ice bath, HHF (20.5 mL, 18.00 g, 0.079 mol, 1 equiv.) was added dropwise within 30 min under vigorous stirring. Subsequently, the reaction was stirred at r.t. until completion (*t*: 20 - 24 h) and quenched with ice-water mixture. The aqueous layer was extracted with diethyl ether (DEE) three times and washed with saturated sodium chloride until neutrality. After combining the organic phase and drying over magnesium sulfate, DEE was removed under reduced pressure. After an initial facile Kugelrohr distillation, 3-Chloroallyl glycidyl ether (3‑Chloro-AGE), which is a known side product for the phase transfer catalysis, was removed via in vacuo distillation (*p*= 5∙10^‑3^mbar, *T*_b,3-Chloro-AGE_ = 30 °C). A final purification was performed via Kugelrohr distillation and liquid HHFarGE (*p* = 5∙10^-3^ mbar, *T*_Kugelrohr, HHFarGE_ = 160 °C) was isolated in yields of 78 to 80 %.

Protic impurities in the monomer were deactivated by treatment with methyl iodide (MeI) and sodium hydride (NaH), resulting in *O*-methylation of any residual terpenoid (Scheme S 2).^18,19^ Thus, a flame-dried Schlenk flask, filled with NaH (0.272 g, 11.0 mmol, 0.22 equiv.), was cooled to 0 °C while the HHFarGE monomer (14.635 g, 51.5 mmol, 1.00 equiv.) was added. Hydrogen gas formation was complete after 2 h and MeI (0.73 g, 5.0 mmol, 0.10 equiv.) was syringed into the flask and the reaction mixture was allowed to warm up to r.t.. After stirring for 22 h, hexane was added to the dilute the reaction suspension before filtration, which removes accrued sodium iodide during methylation. After a Kugelrohr distillation HHFarGE was isolated as a colorless liquid in quantitative yields. The pretreatment reaction simultaneously serves as extensive drying process.

**Characterization of HHFarGE.**


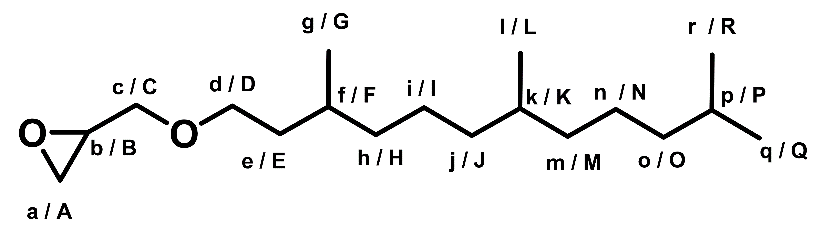


^1^H NMR (400 MHz, chloroform-*d*_1_): δ (ppm) = 3.72 – 3.67 (dt, *J* = 11.5, 3.5 Hz, 1H, H_c_), 3.59 – 3.43 (m, 2H, H_d_), 3.42 – 3.30 (m, 1H, H_c_), 3.20 – 3.05 (m, 1H, H_b_), 2.85 – 2.71 (m, 1H, H_a_), 2.67 – 2.52 (m, 1H, H_a_), 1.71 – 0.98 (m, 17H, H_e_, H_f_ H_h_, H_i_, H_j_, H_k_, H_m_, H_n_, H_o_, H_p_), 0.93 – 0.77 (m, 12H, H_g_, H_l_, H_r_, H_q_).

^13^C NMR (100 MHz, chloroform-*d*_1_): δ (ppm) = 71.61 (C_C_), 70.14 + 70.11 (C_D_), 51.04 (C_B_), 44.48 + 44.46 (C_A_), 39.48 (C_O_), 37.61 + 37.57 (C_M_), 37.49 (C_H_), 37.45 + 37.38 (C_J_), 36.86 + 36.77 (C_E_), 32.90 (C_K_), 29.96 + 29.94 (C_F_), 28.10 (C_P_), 24.94 + 24.92 (C_N_), 24.47 (C_I_), 22.85, 22.76 (C_Q_ + C_R_), 19.86 + 19.82 + 19.79 + 19.76 (C_G_ + C_L_).

**Characterization of FarGE.**

A detailed NMR characterization and the respective NMR spectra have been previously published.^18^ In accordance, the stereochemistry-induced carbon shifts are labelled according to the respective farnesol stereoisomer, using *E* (*trans*) and *Z* (*cis*) nomenclature for the C-2 and C-6 carbon in lower cases. Hence, carbon atoms are distinguished as follows: X*_EE_* (2*E*,6*E*‑farnesol), X*_EZ_* (2*E*,6*Z*-farnesol) and X*_ZE_* (2*Z*,6*E*-farnesol).


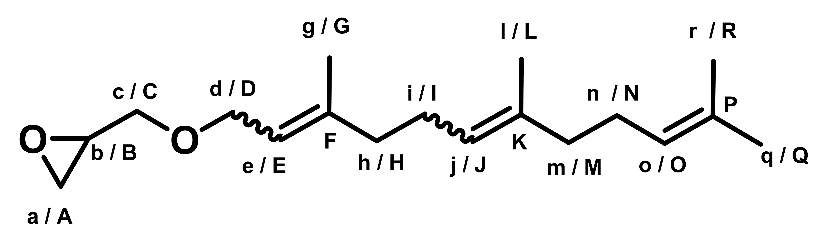


Purification by Kugelrohr distillation: *p* = 5∙10^-3^ mbar, *T*_Kugelrohr, FarGE_ = 170 °C

^1^H NMR (400 MHz, chloroform-*d*_1_): δ (ppm) = 5.36–5.29 (m, 1H, H_e_), 5.11–5.03 (m, 2H, H_j,_ H_o_),

4.09–3.97 (m, 2H, H_d_), 3.71–3.60 (m, 1H, H_c_), 3.39–3.32 (m, 1H, H_c_), 3.17–3.08 (m, 1H, H_b_), 2.79–2.70 (m, 1H, H_a_), 2.62–2.51 (m, 1H, H_a_), 2.18–1.88 (m, 8H, H_h_, H_i_ H_m_, H_n_), 1.73+1.85+1.57 (s, 12H, H_g_, H_l_ H_r_, H_q_, *J* = 5.0, 4.2 Hz).

^13^C NMR (100 MHz, chloroform-*d*_1_): δ (ppm) = 140.93 + 140.68 (C_F(_*_ZE_*_)_), (C_F(_*_EE_*_)_), 140.64 (C_F(_*_EZ_*_)_), 135.61 (C_K(_*_ZE_*_)_), 135.46 (C_K(_*_EZ_*_)_), 135.31 (C_K(_*_EE_*_)_), 131.54 (C_P(_*_EZ_*_)_), 131.34 (C_P(_*_ZE_*_)_), 131.29 (C_P(_*_EE_*_)_), 124.67(C_J(_*_EZ_*_)_), 124.37 + 124.34 (C_O(_*_EE_*_,_ *_EZ_*_,_ *_ZE_*_)_), 123.86(C_J(_*_EE_*_)_), 123.66 (C_J(_*_ZE_*_)_), 121.53 (C_E(_*_ZE_*_)_), 120.55 (C_E(_*_EE_*_,_ *_EZ_*_)_), 70.73(C_C(_*_ZE_*_)_), 70.66 (C_C(_*_EE,EZ_*_)_), 67.79 (C_D(_*_EE, EZ_*_)_), 67.54 (C_D(_*_ZE_*_)_), 50.90 (C_B_), 44.44 (C_A_), 39.92 (C_H(_*_EZ_*_)_), 39.74 (C_M(_*_EE_*_,_*_ZE_*_)_), 39.63 (C_H(_*_EE_*_)_), 32.27 (C_H(_*_ZE_*_)_), 32.01 (C_M(_*_EZ_*_)_), 26.76 (C_N(_*_EE_*_)_), 26.71 (C_N(_*_ZE_*_)_), 26.63 (C_N(_*_EZ_*_)_), (C_I(_*_ZE_*_)_), 26.31 (C_I(_*_EE_*_)_), 26.18 (C_I(_*_EZ_*_)_), 25.76 (C_Q(_*_EZ_*_)_), 25.73 (C_Q(_*_EE,ZE_*_)_), 23.55 (C_G(_*_ZE_*_)_), 23.41 (C_L(_*_EZ_*_)_), 17.71 (C_R(_*_EE_*_,_*_ZE_*_)_), 17.67 (C_R(_*_EZ_*_)_), 16.52 (C_G(_*_EE,EZ_*_)_), 16.03 (C_L(_*_EE,ZE_*_)_).

1. Polymerization Procedures

**Synthesis procedure for diblock copolymerization of and FarGE and HHFarGE with mPEG_114_**

Two series of diblock copolymers, mPEG_114_-*b*-FarGE­_m_ and mPEG_114_-*b*-HHFarGE­_m_ respectively, were synthesized using mPEG_114_ as macroinitiator in a crown-ether-assisted anionic ring opening polymerization.^18^ Protic impurities contained in the TGEs were methylated using MeI if not noted otherwise in Table 1. Note that a detailed mPEG_114_-*b*-PFarGE_m_ and PFarGE characterization will not be discussed as it has been published previously.^18^

The following protocol illustrates the general procedure for the diblock copolymer synthesis, exemplarily for mPEG_114_-*b*-HHFarGE_8_ (Table 1, entry 12). mPEG_114_ (1.500 g, 0.30 mmol, 1.0 equiv.), potassium *tert*-butoxide (KO^t^Bu, 0.027 g, 0.24 mmol, 0.8 equiv.) and 18-crown-6 (0.127 g, 0.48 mmol, 2.0 equiv.) were mixed in a dry Schlenk flask applying positive nitrogen flow, suspended in benzene and stirred for 1 h at 60 °C under vacuum to generate the initiator salt. Following, the initiator salt was dried azeotropically overnight at 60 °C. The dry monomer HHFarGE (0.83 mL, 0.770 g, 2.7 mmol, 9.0 equiv) was syringed into the flask and the reaction mixture was stirred for 24 h at 80 °C. Note that the polymerization temperature was increased up to 120 °C (with incremental *P*_n_) as the viscosity increase impeded sufficient stirring at 80 °C. The polymerization was terminated after cooling to r.t. by adding 1 mL of methanol and the polymer was dissolved in dichloromethane (DCM). Three-fold precipitation in cold diethyl ether (‑20 °C) allowed for the removal of homopolymer residues. After dialysis (MWCO = 1000 g∙mol^-1^) in Milli-Q®, enabling the removal of traces of 18-crown-6 and potassium salts, the polymer was obtained in yields of 70 – 95 %.


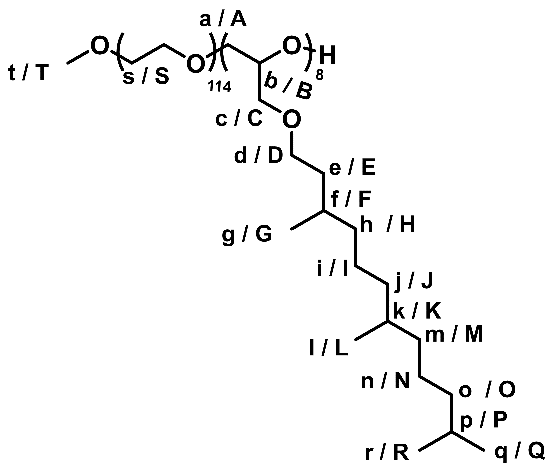


^1^H NMR (400 MHz, chloroform-*d*_1_): δ (ppm) = 3.87 – 3.34 (m, H_a_, H_b_, H_c_, H_d_, H_s_), 3.34 (s, H_t_), 1.67 – 0.94 (m, H_e_, H_f_, H_h_, H_i_, H_j_, H_k_, H_m_, H_n_, H_o_), 0.94 – 0.68 (m, H_g_, H_l_ H_r_, H_q_).

^13^C NMR (100 MHz, chloroform-*d*_1_): δ (ppm) = 78.84, 78.63, 78.37 (C_B_), 71.98, 71.84, 70.83, 70.78, 70.67, 70.61, 70.58, 70.07, 70.02, 69.99 (C_A_, C_C_, C_D_, C_S_), 59.09 (C_T_), 39.41 (C_O_), 37.60, 37.53, 37.48, 37.36, 37.33, 37.32 (C_M_, C_H_, C_J_), 36.82, 36.74, 36.66 (C_E_), 32.85 (C_K_), 30.06 + 29.98 (C_F_), 28.01 (C_P_), 24.86, 24.48, 24.43 (C_N_, C_I_), 22.79 + 22.68 (C_Q_, C_R_), 19.78, 19.71 (C_G_, C_L_,).

SEC analysis (RI detector, eluent: DMF, standard: PEG): *M*_n_ = 6600 g·mol^-1^; *Đ* = 1.06

*T*_g_ = -71 °C *T*_m_ = 56 °C

The following characterization refers to mPEG_114_-*b*-FarGE­_9_ (Table 1 and 2, entry 5). The polymer characterization and the respective NMR spectra have been previously published.^18^


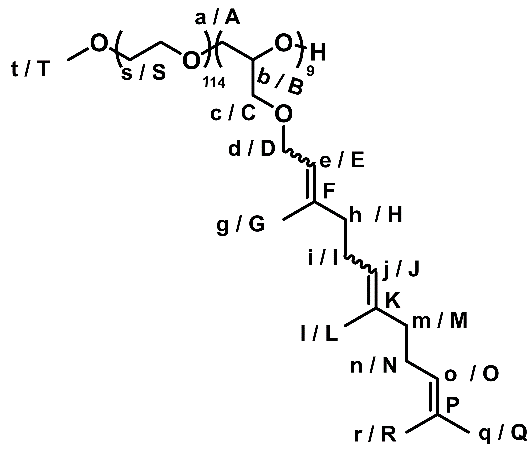


^1^H NMR (400 MHz, chloroform-*d*_1_): δ (ppm) = 5.39–5.22 (m, H_e_), 5.20–4.98 (m, H_j,_ H_o_), 4.04–3.86 (m, H_d_), 3.83–3.38 (m, H_a_, H_b_, H_c_, H_s_), 3.36 (s, H_t_), 2.13–1.84 (m, H_h_, H_i_, H_m_, H_n_), 1.75–1.41 (m, H_g_, H_l_ H_r_, H_q_).

^13^C NMR (100 MHz, chloroform-*d*_1_): δ (ppm) = 140.01, 139.83, 139.71 (C_F_), 135.51, 135.40, 135.25 (C_K_),

131.54, 131.31 (C_P_), 124.74, 124.40, 123.95, 123.73 (C_O_, C_J_), 121.24, 121.14, 121.07, 120.75 (C_E_), 78.87, 78.68, 78.37 (C_B_), 71.98, 71.20, 71.07, 70.82, 70.61, 70.14, 69.87, 69.70 (C_A_, C_C_, C_S_), 67.93, 67.62 (C_D_), 59.09 (C_T_), 40.01, 39.76, 39.73, 32.34, 32.01 (C_H_, C_M_), 26.78, 26.64, 26.53, 26.46, 26.43, 26.27, 26.21 (C_N_, C_I_), 25.80, 25.76 (C_Q_), 23.62, 23.59, 23.45 (C_G_, C_L_,), 17.75, 17.71 (C_R_),16.59, 16.51 (C_G_) 16.06 (C_L_,).

SEC analysis (RI detector, eluent: DMF, standard: PEG): *M*_n_ = 7100 g·mol^-1^; *Đ* = 1.05

*T*_g_ = -71 °C *T*_m_ = 56 °C

**Synthesis procedure for PHHFarGE and PFarGE homopolymer**

The homopolymerization was performed with 2‑(benzyloxy)ethanol (BzEtOH) as initiator while following the diblock copolymer synthesis protocol. Purification was achieved either via threefold precipitation in cold methanol (-20 °C) or dialysis, followed by drying under reduced pressure overnight. PHHFarGE_10_ is characterized exemplary in the following (Table 1 and 2, entry 13).


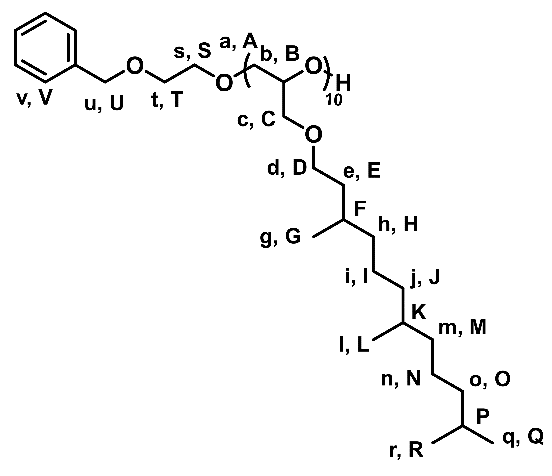


^1^H NMR (400 MHz, chloroform-*d*_1_): δ (ppm) = 7.38 – 7.31 (m, H_v_), 4.56 (s, H_u_), 3.97 – 3.26 (m, H_a_, H_b_, H_c_, H_d_, H_s,_ H_t_), 1.69 – 0.94 (m, H_e_, H_f_, H_h_, H_i_, H_j_, H_k_, H_m_, H_n_, H_o_), 0.94 – 0.70 (m, H_g_, H_l_ H_r_, H_q_).

^13^C NMR (100 MHz, chloroform-*d*_1_): δ (ppm) = 128.49, 127.80, 127.70 (C_V_), 78.95, 78.77 (C_B_), 73.36, 72.05, 71.96, 71.45, 71.06, 70.77, 70.26, 70.19, 69.89, 69.71, 69.58 (C_A_, C_C_, C_D_, C_S_, C_T_), 39.52 (C_O_), 37.81, 37.71, 37.65, 37.58, 37.56, 37.48, 37.45, 37.03 (C_M_, C_H_, C_J_), 36.94, 36.89, 36.86, 36.78 (C_E_), 32.96 (C_K_), 30.18, 30.15, 30.10 (C_F_), 28.09 (C_P_), 24.98, 24.58, 24.55, 24.51 (C_N_, C_I_), 22.88, 22.79 (C_Q_, C_R_), 19.88, 19.81 (C_G_, C_L_,).

SEC analysis (RI detector, eluent: THF, standard: PEG): *M*_n_ = 3300 g·mol^-1^; *Đ* = 1.12

*T*_g_ = -74 °C


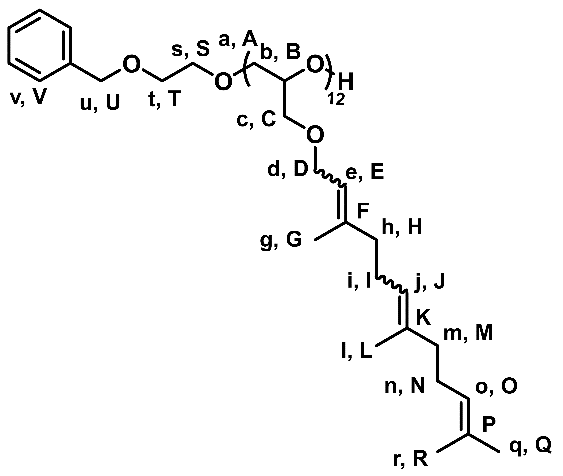


^1^H NMR (400 MHz, chloroform-*d*_1_): δ (ppm) = 7.36 – 7.29 (m, H_v_), 5.38 – 5.25 (m, H_e_), 5.17 – 5.02 (m, H_j,_ H_o_), 4.55 (s, H_u_), 4.09 – 3.88 (m, H_d_), 3.88 – 3.32 (m, H_a_, H_b_, H_c_, H_s,_ H_t_), 2.19 – 1.85 (m, H_h_, H_i_, H_m_, H_n_), 1.83 – 1.43 (m, H_g_, H_l_ H_r_, H_q_).

^13^C NMR (100 MHz, chloroform-*d*_1_): δ (ppm) = 140.14, 139.90, 139.82, 139.67 (C_F_), 135.50, 135.44, 135.36 (C_K_), 131.64, 131.41 (C_P_), 128.48, 127.80, 127.68 (C_V_), 124.84, 124.49, 124.47, 124.07, 124.05, 123.87 (C_O_, C_J_), 121.37, 121.25, 121.17, 121.09, 121.02, 120.93, 120.85, 120.82 (C_E_), 79.10, 78.98, 78.81 (C_B_), 73.34 (C_U_), 71.45, 71.22, 71.03, 70.48, 70.40, 70.25, 70.03, 69.92, 69.82, 69.57 (C_A,_ C_C,_ C_S,_ C_T_), 68.06, 68.03, 67.73 (C_D_), 40.11, 40.06, 39.86, 39.83, 39.79, 32.44, 32.11 (C_H_, C_M_), 26.88, 26.86, 26.74, 26.64, 26.61, 26.56, 26.53, 26.37, 26.34 (C_N_, C_I_), 25.88, 25.85 (C_Q_), 23.70, 23.65, 23.54 (C_G_, C_L_,), 17.83, 17.80 (C_R_), 16.68, 16.66, 16.60 (C_G_), 16.14 (C_L_).

SEC analysis (RI detector, eluent: THF, standard: PEG): *M*_n_ = 3500 g·mol^-1^; *Đ* = 1.20

*T*_g_ = -75 °C

1. NMR and IR Spectra of HHF and HHFarGE


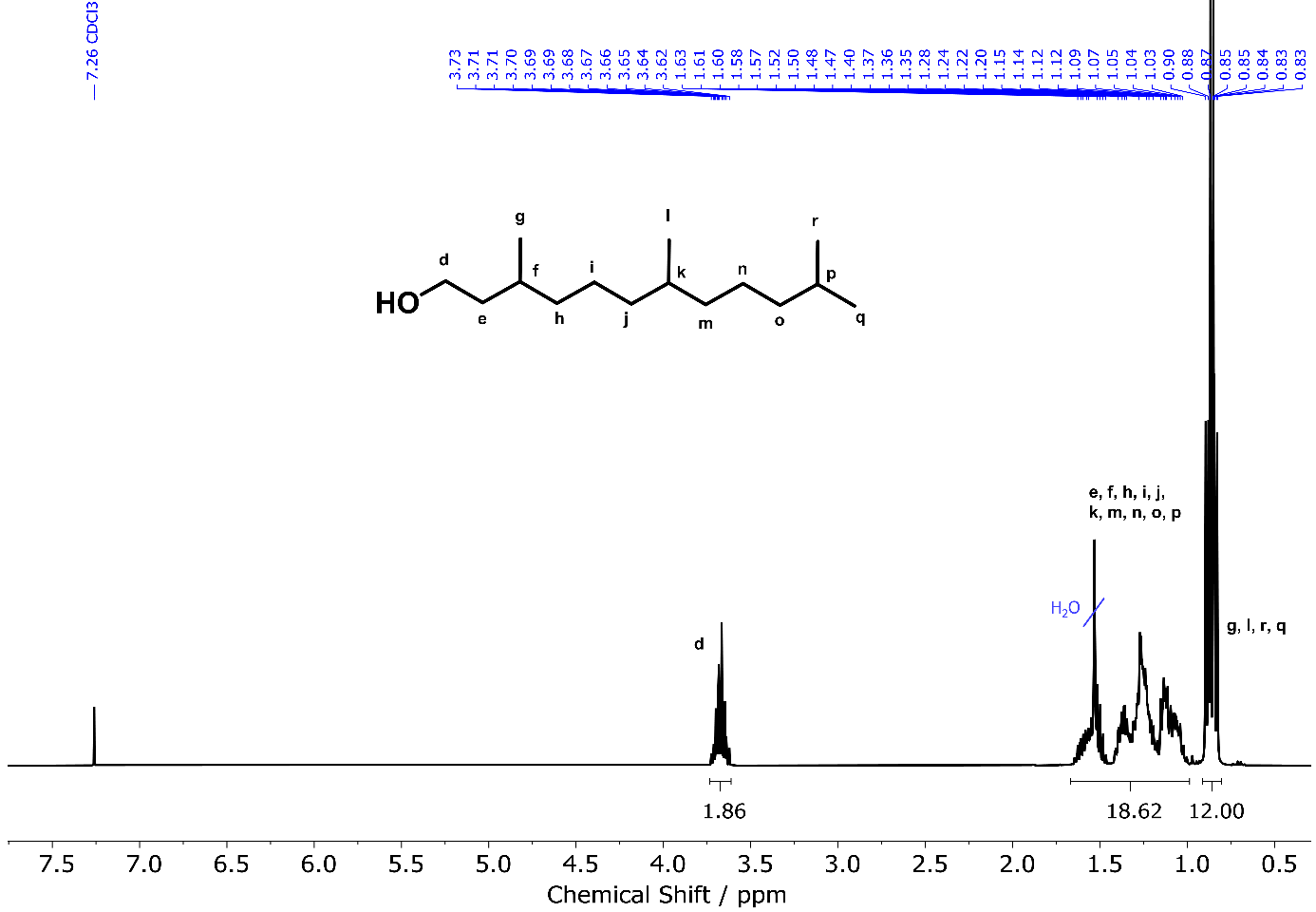


Figure S1: ^1^H NMR spectrum (400 MHz, CDCl_3_) of HHF.


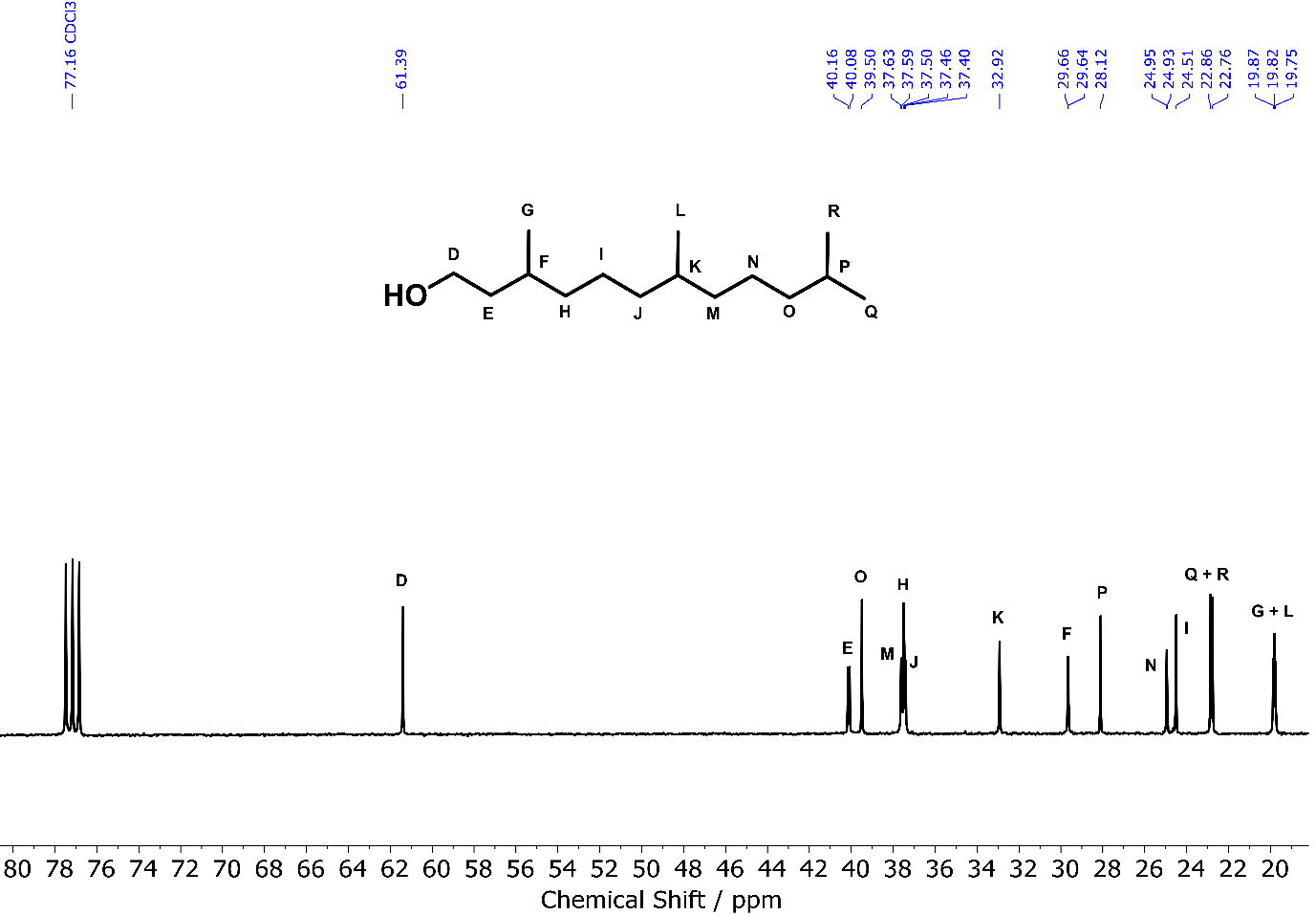


Figure S2: ^13^C NMR spectrum (100 MHz, CDCl_3_) of HHF.


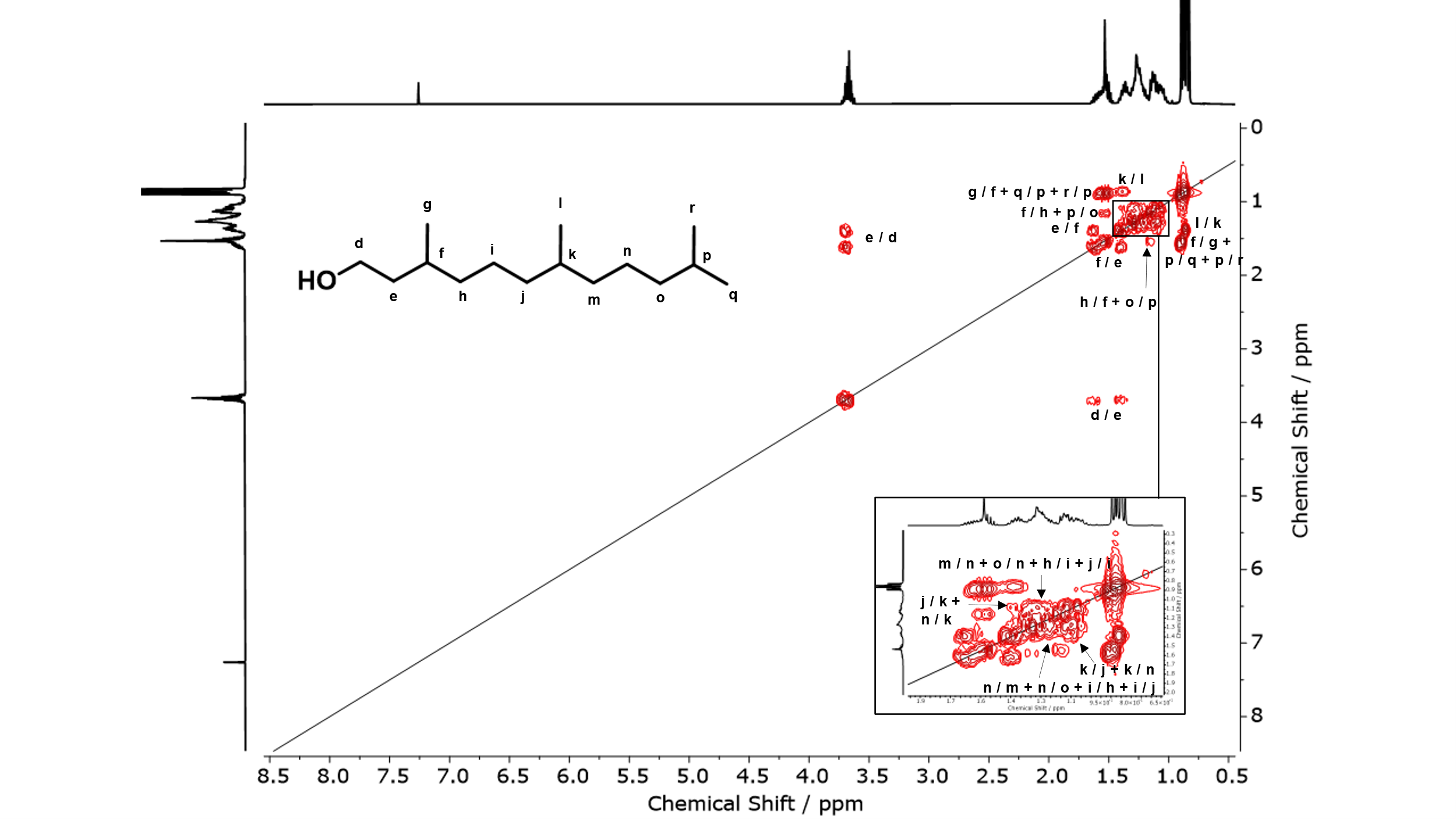


Figure S3: ^1^H-^1^H COSY NMR spectrum (400 MHz, CDCl_3_) of HHF.


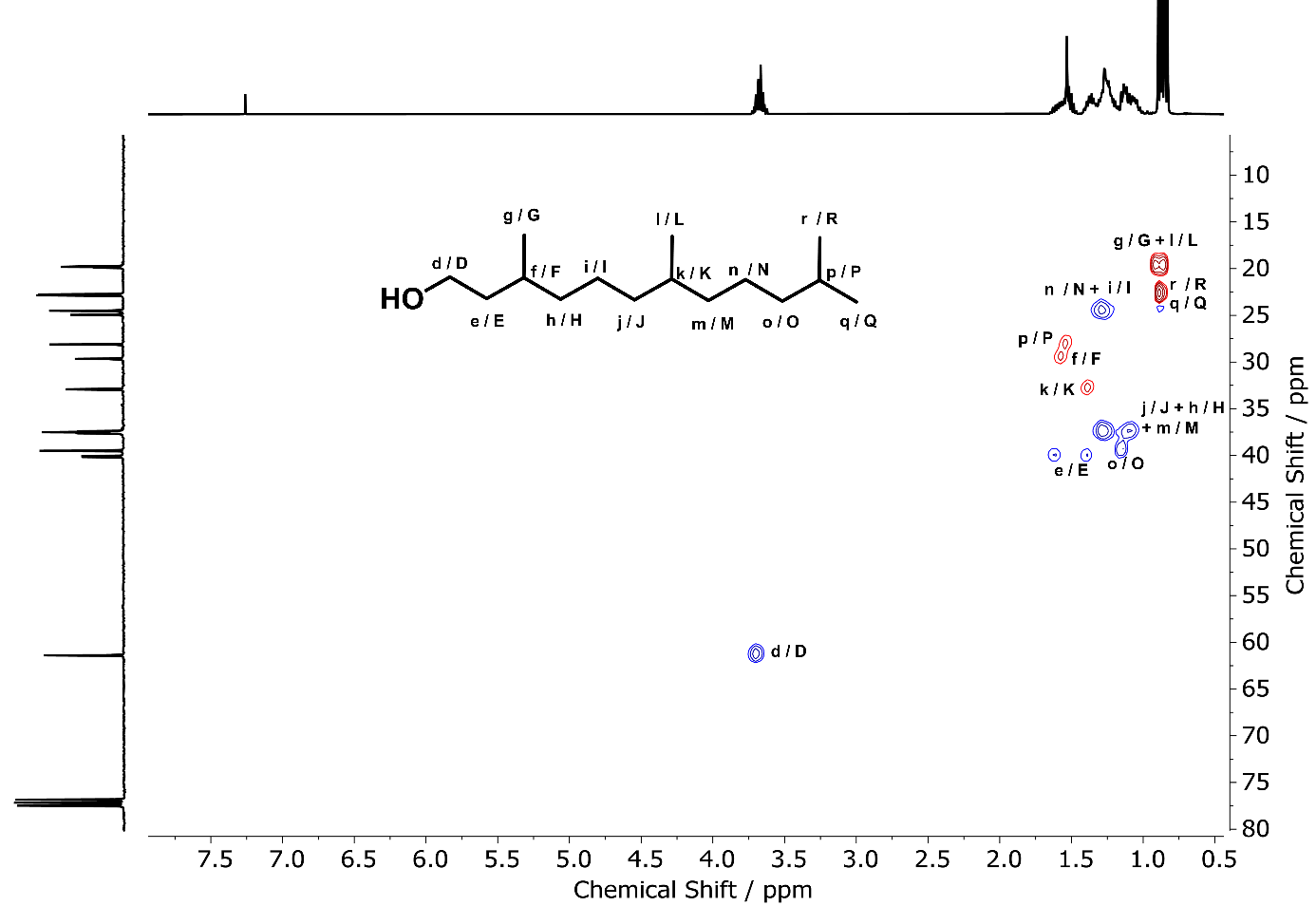


Figure S4: ^1^H-^13^C HSQC NMR spectrum (400 MHz/ 100 MHz, CDCl_3_) of HHF.


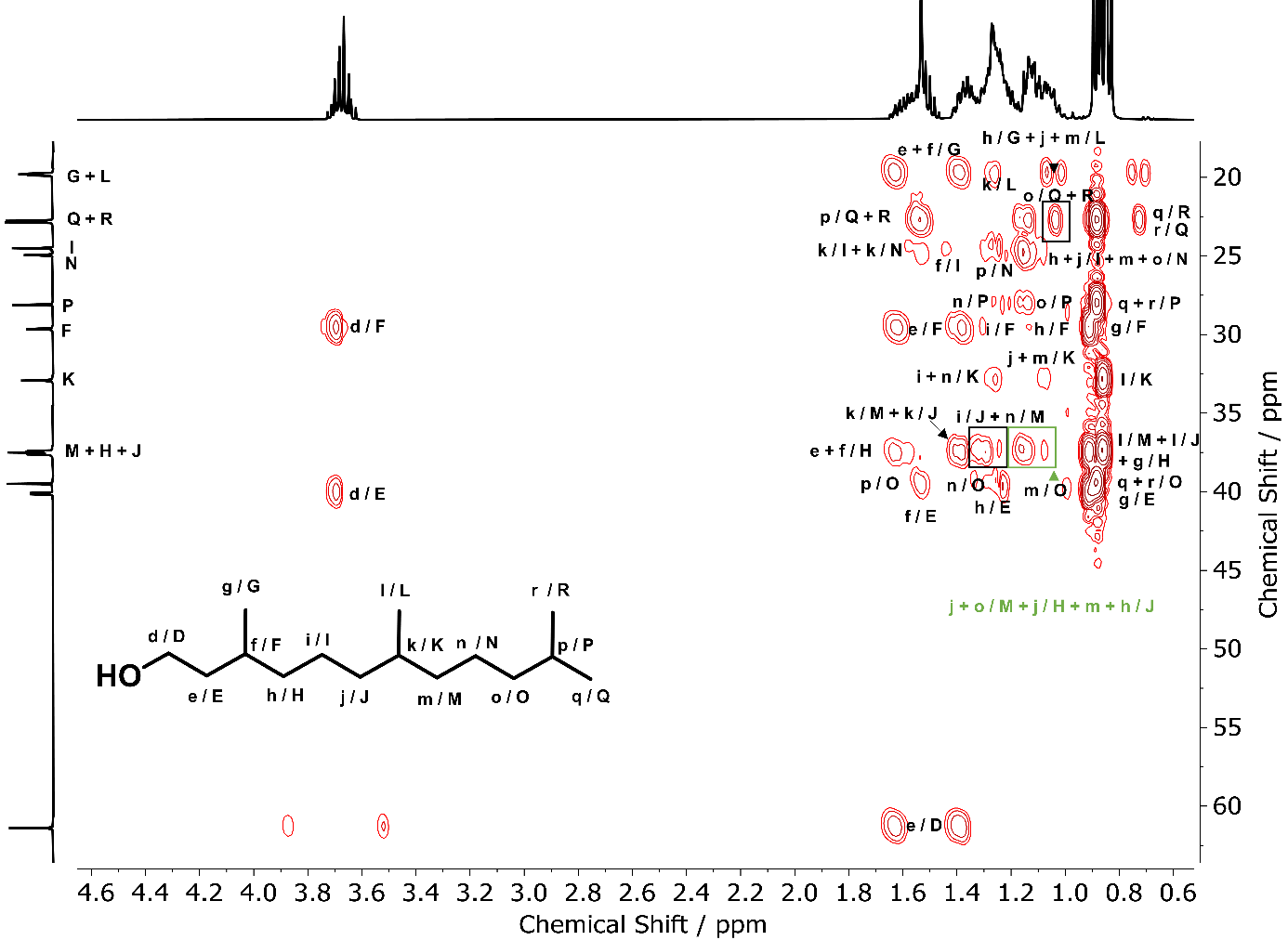


Figure S5: ^1^H-^13^C HMBC NMR spectrum (400 MHz/ 100 MHz, CDCl_3_) of HHF.


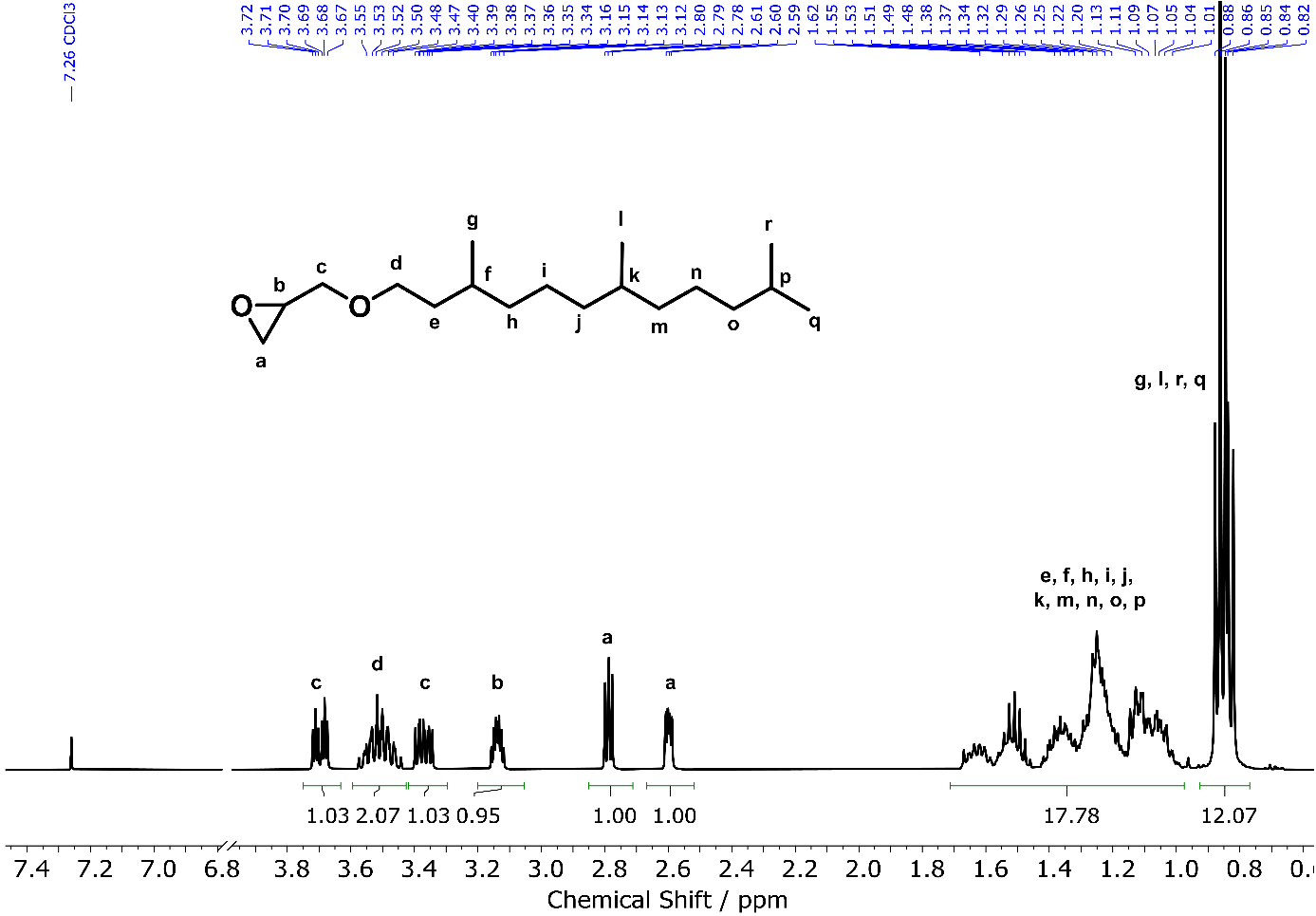


Figure S6: ^1^H NMR spectrum (400 MHz, CDCl_3_) of HHFarGE.

**
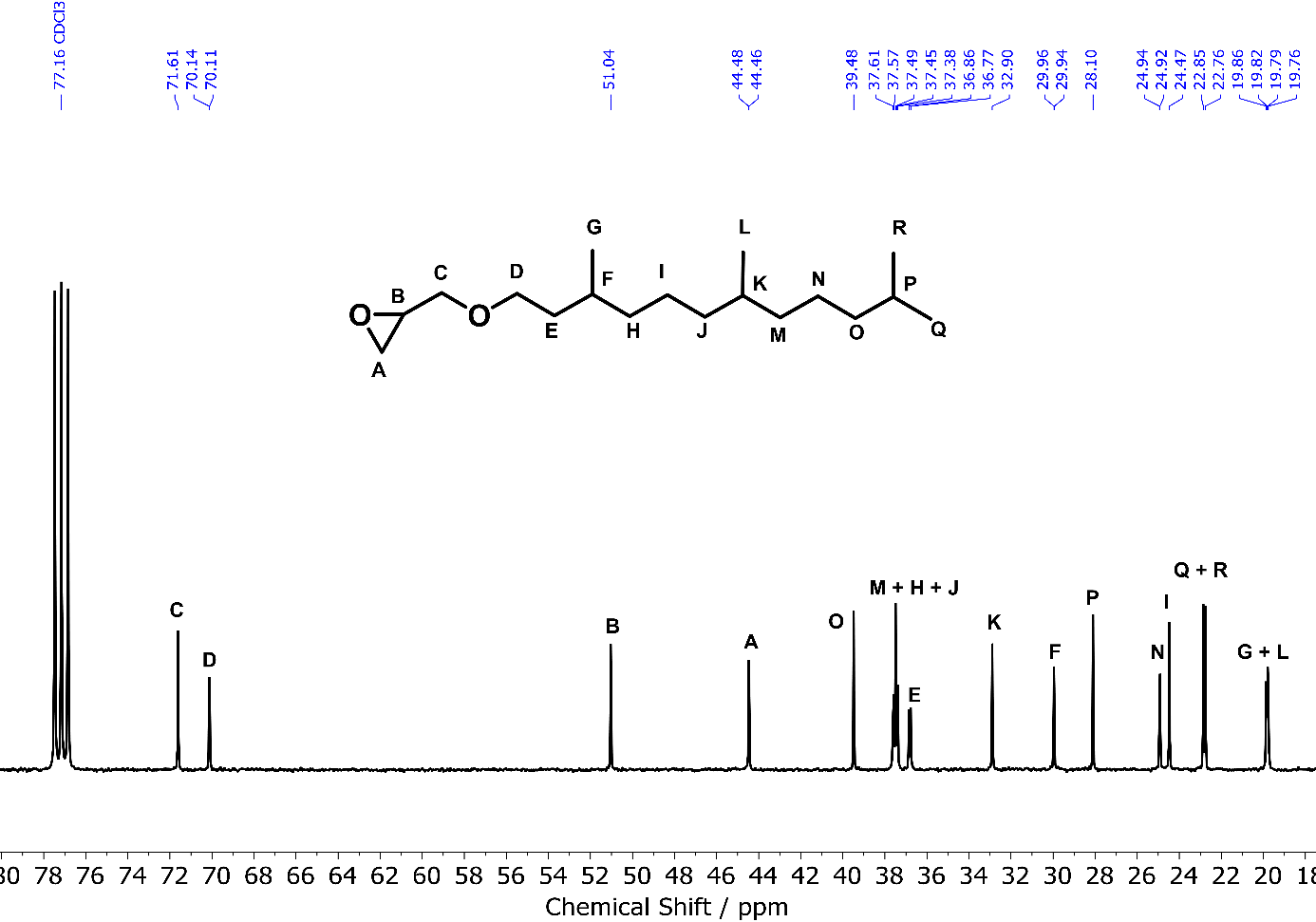
**

Figure S 7: ^13^C NMR spectrum (100 MHz, CDCl_3_) of HHFarGE.


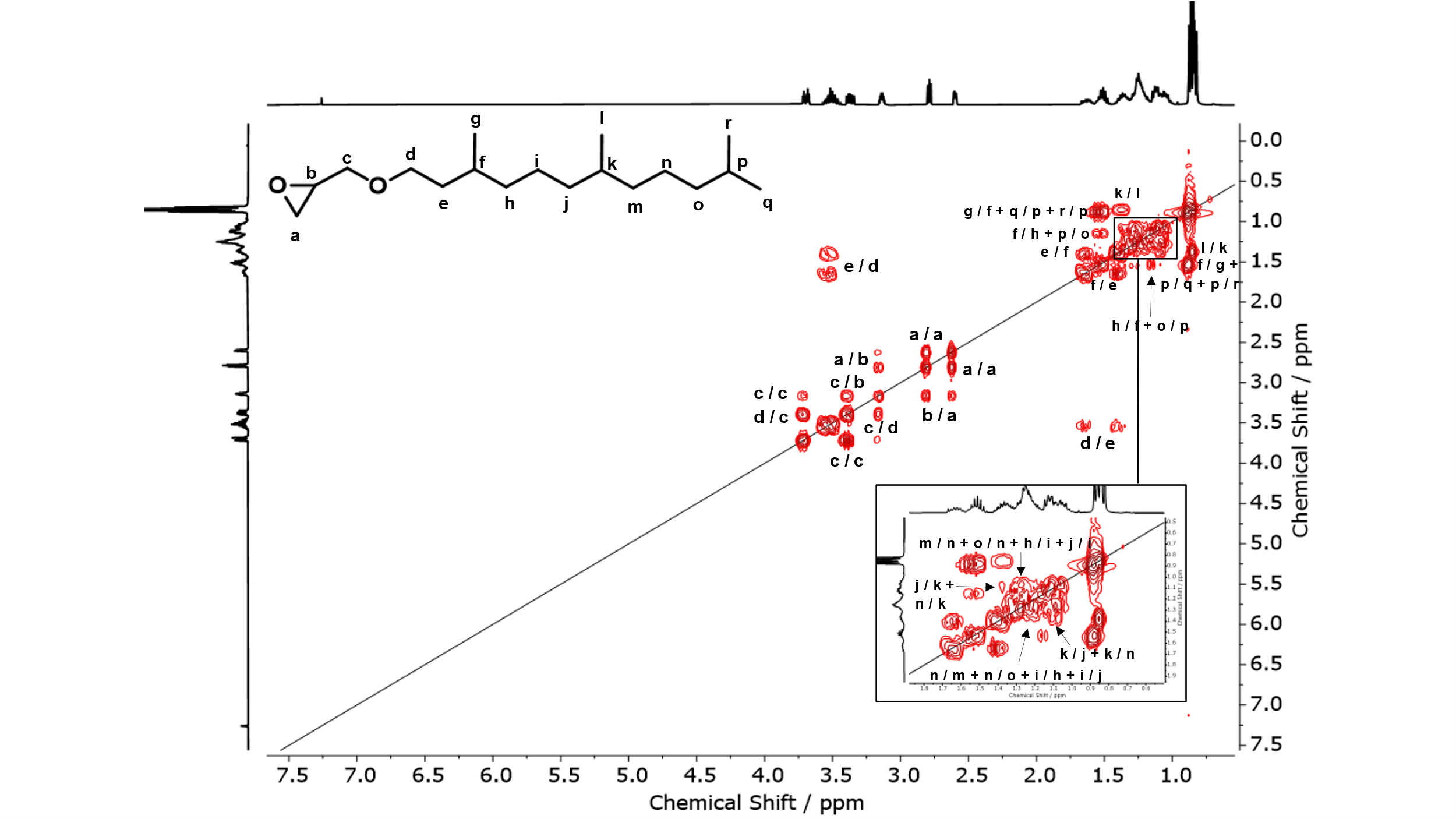
Figure S 8: ^1^H-^1^H COSY NMR spectrum (400 MHz, CDCl_3_) of HHFarGE.


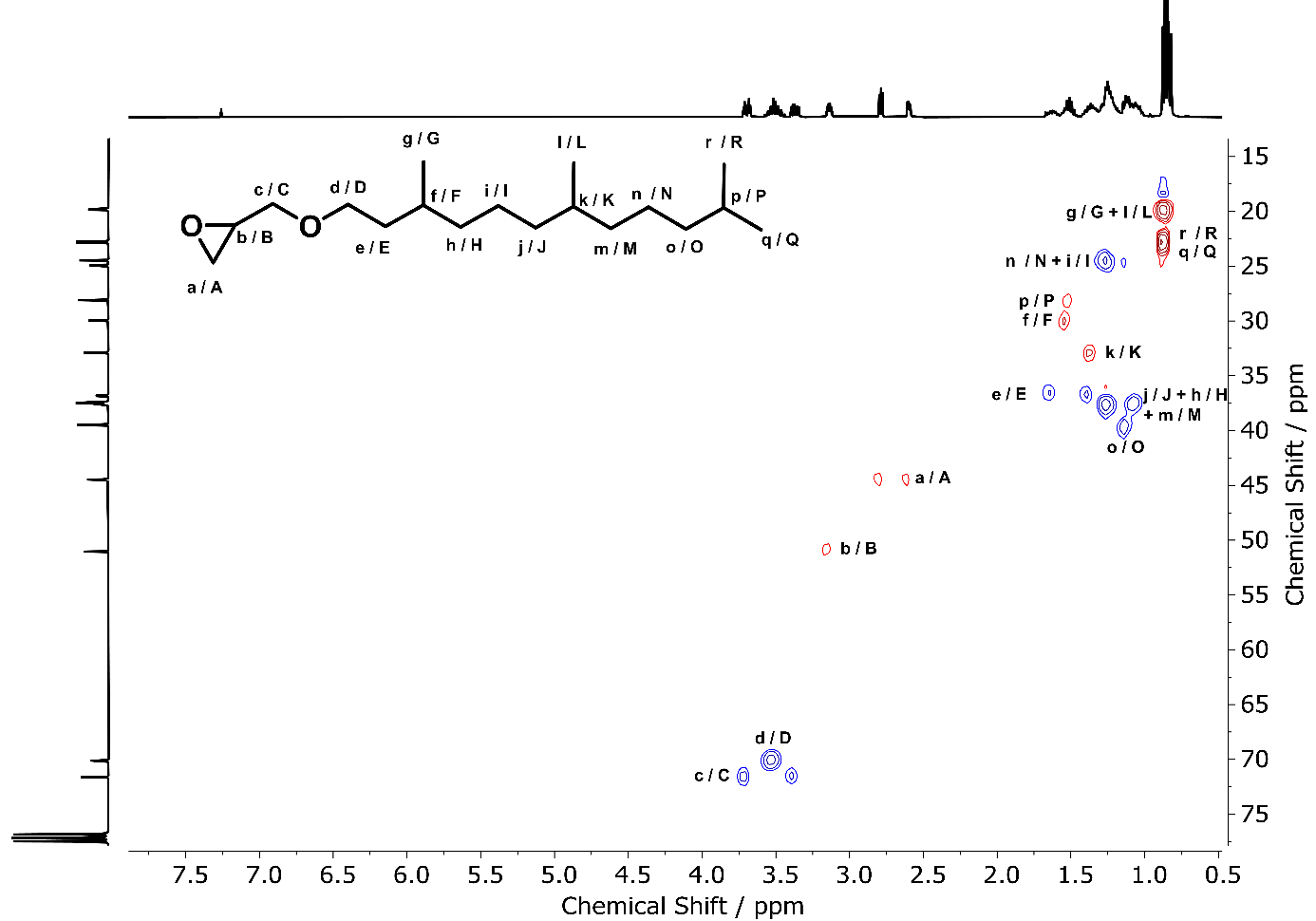


Figure S9: ^1^H-^13^C HSQC NMR spectrum (400 MHz/ 100 MHz, CDCl_3_) of HHFarGE.


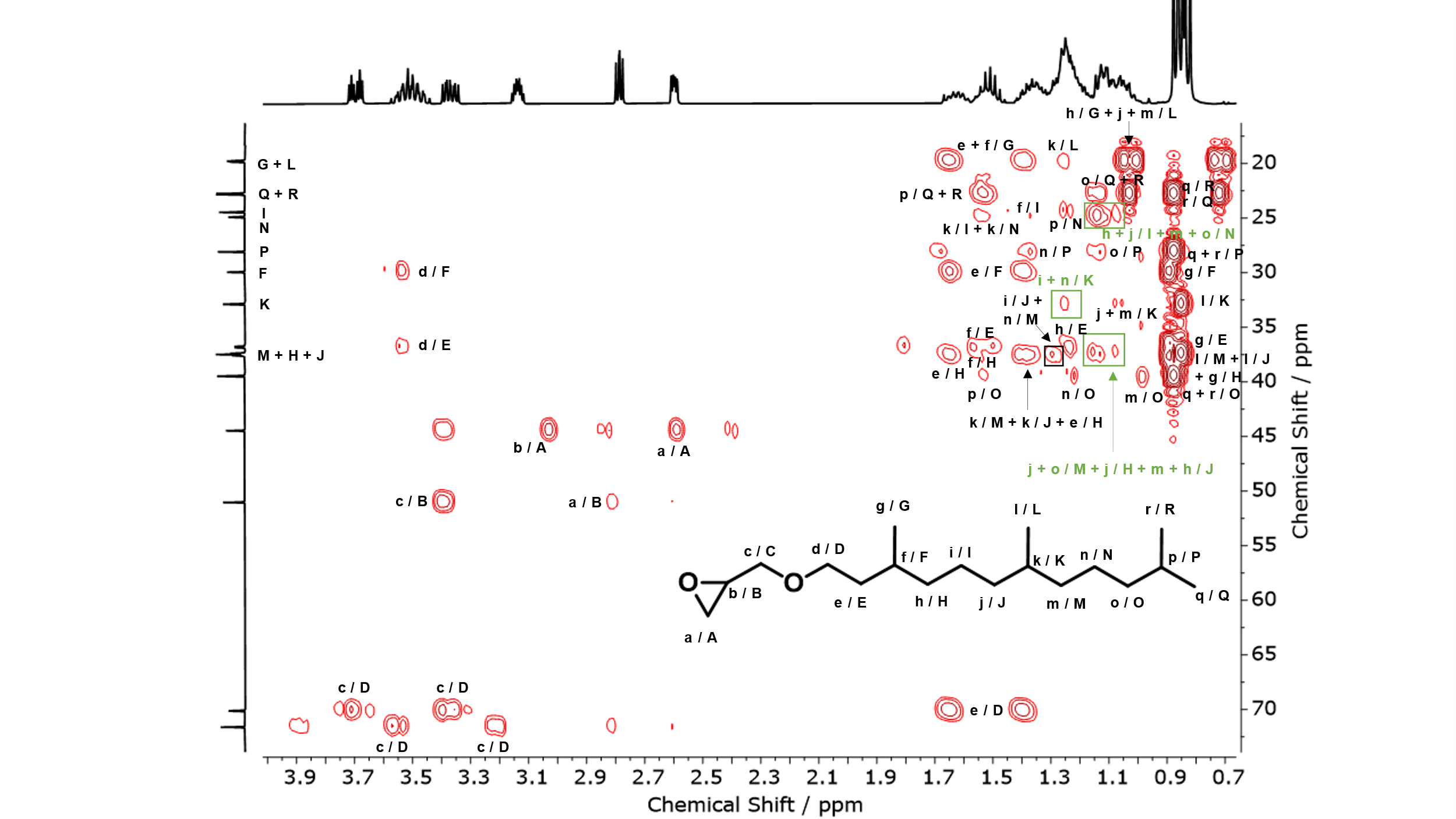


Figure S10: ^1^H-^13^C HMBC NMR spectrum (400 MHz/ 100 MHz, CDCl_3_) of HHFarGE.

1. NMR Characterization of Diblock Copolymers of HHFarGE and mPEG_114_


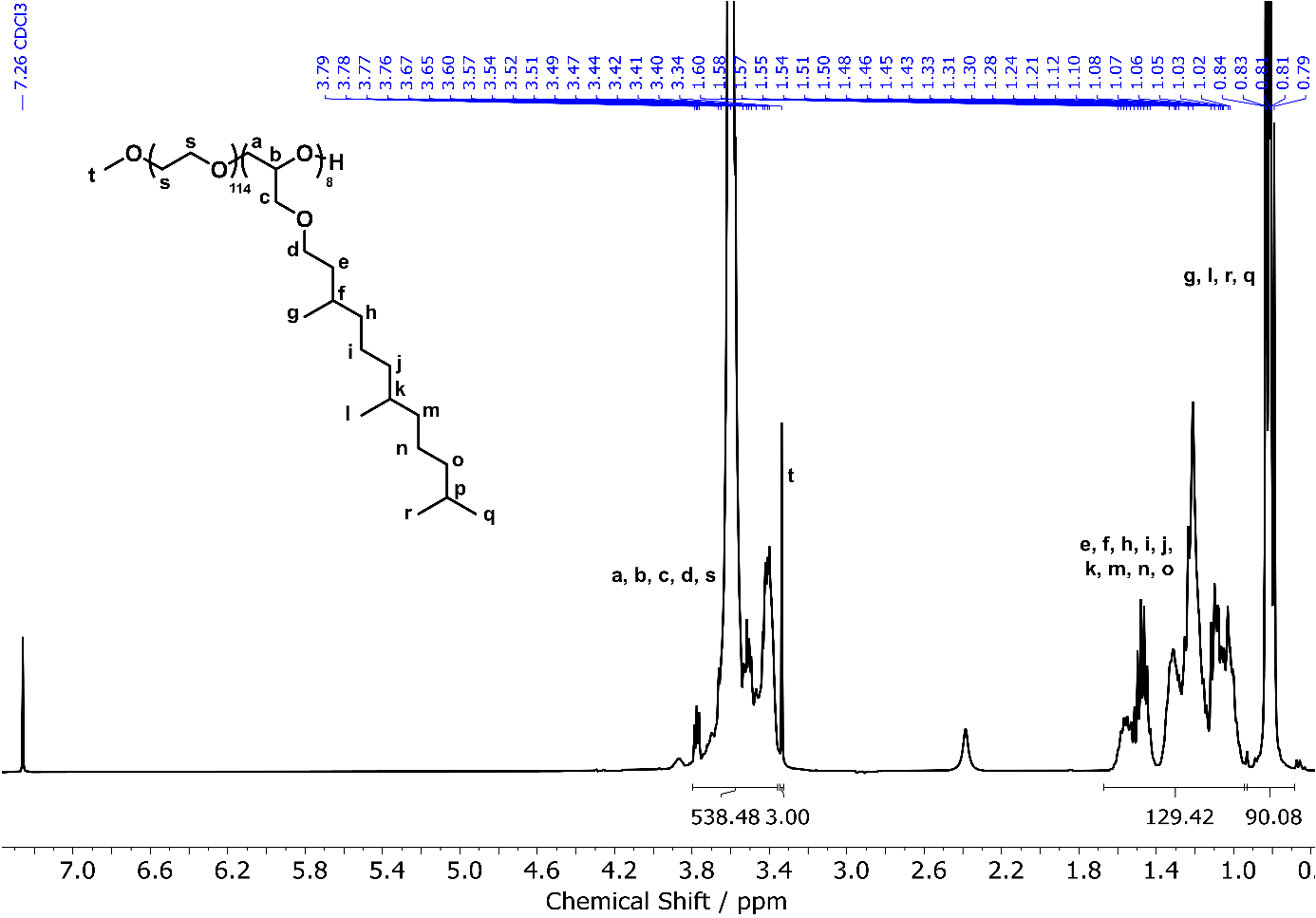


Figure S11: ^1^H NMR spectrum (400 MHz, CDCl_3_) of mPEG_114_-*b*-PHHFarGE_8_.


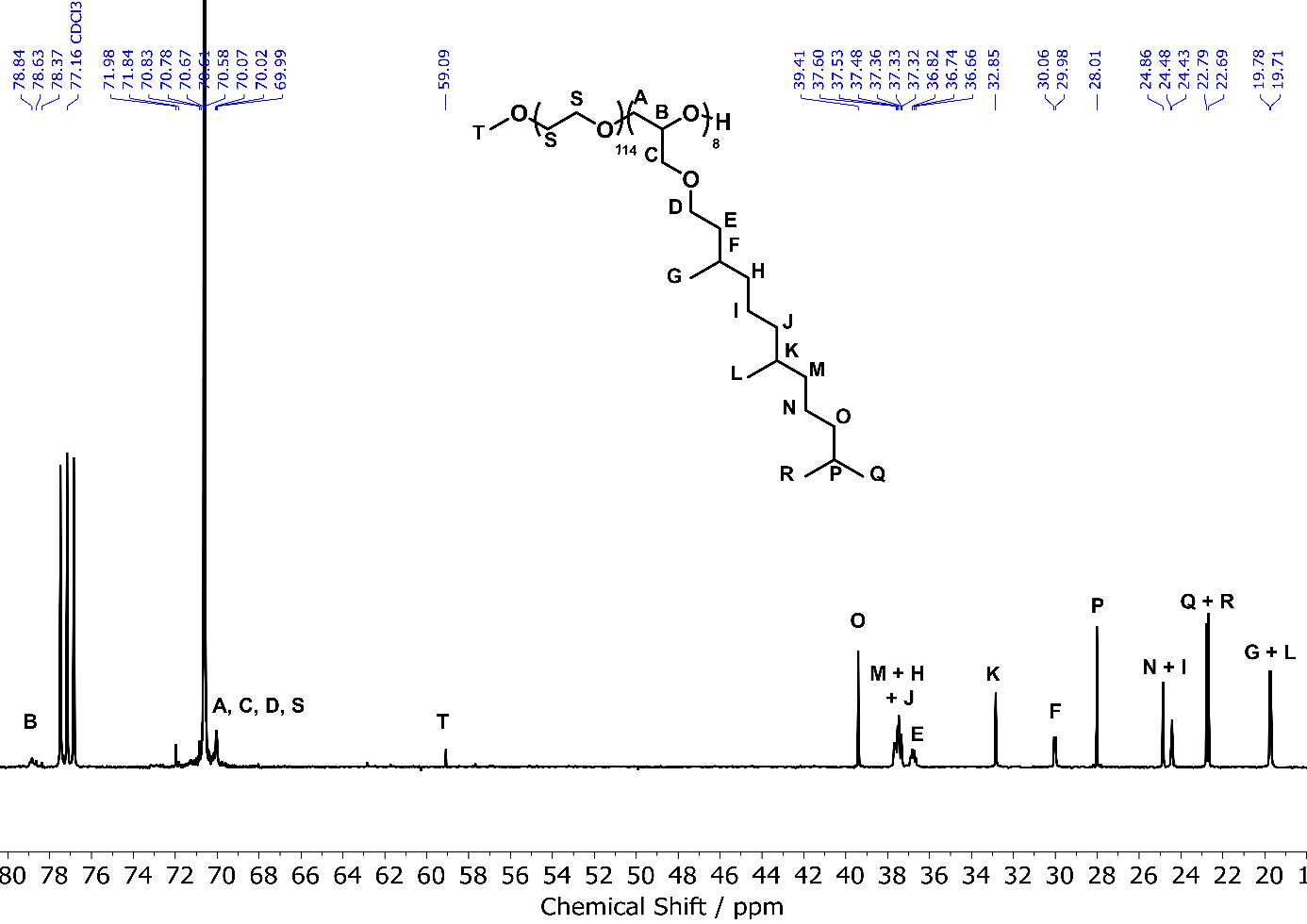


Figure S12: ^13^C NMR spectrum (100 MHz, CDCl_3_) of mPEG_114_-*b*-PHHFarGE_8_.


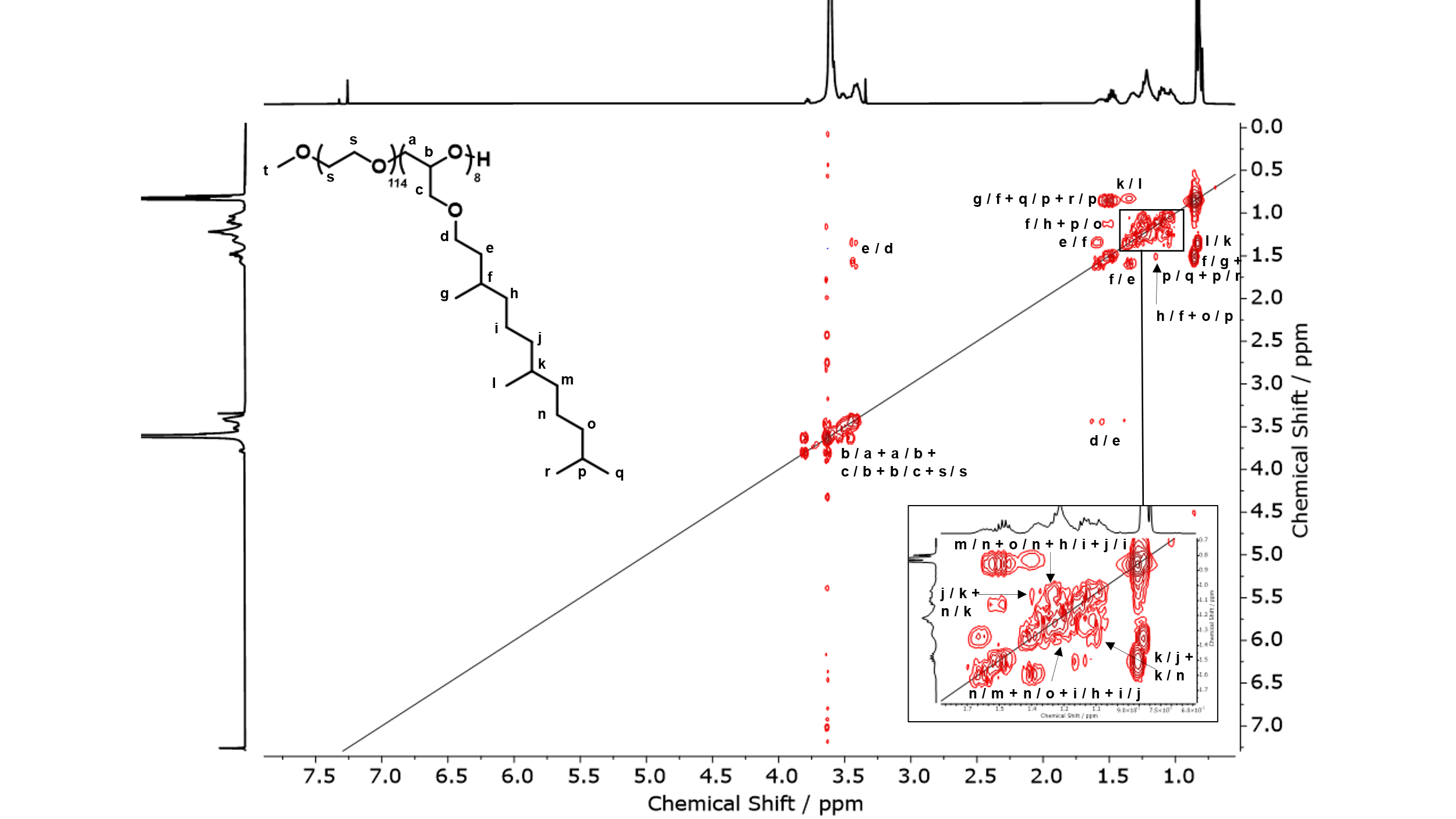


Figure S13: ^1^H-^1^H COSY NMR spectrum (400 MHz, CDCl_3_) of mPEG_114_-*b*-PHHFarGE_8_.


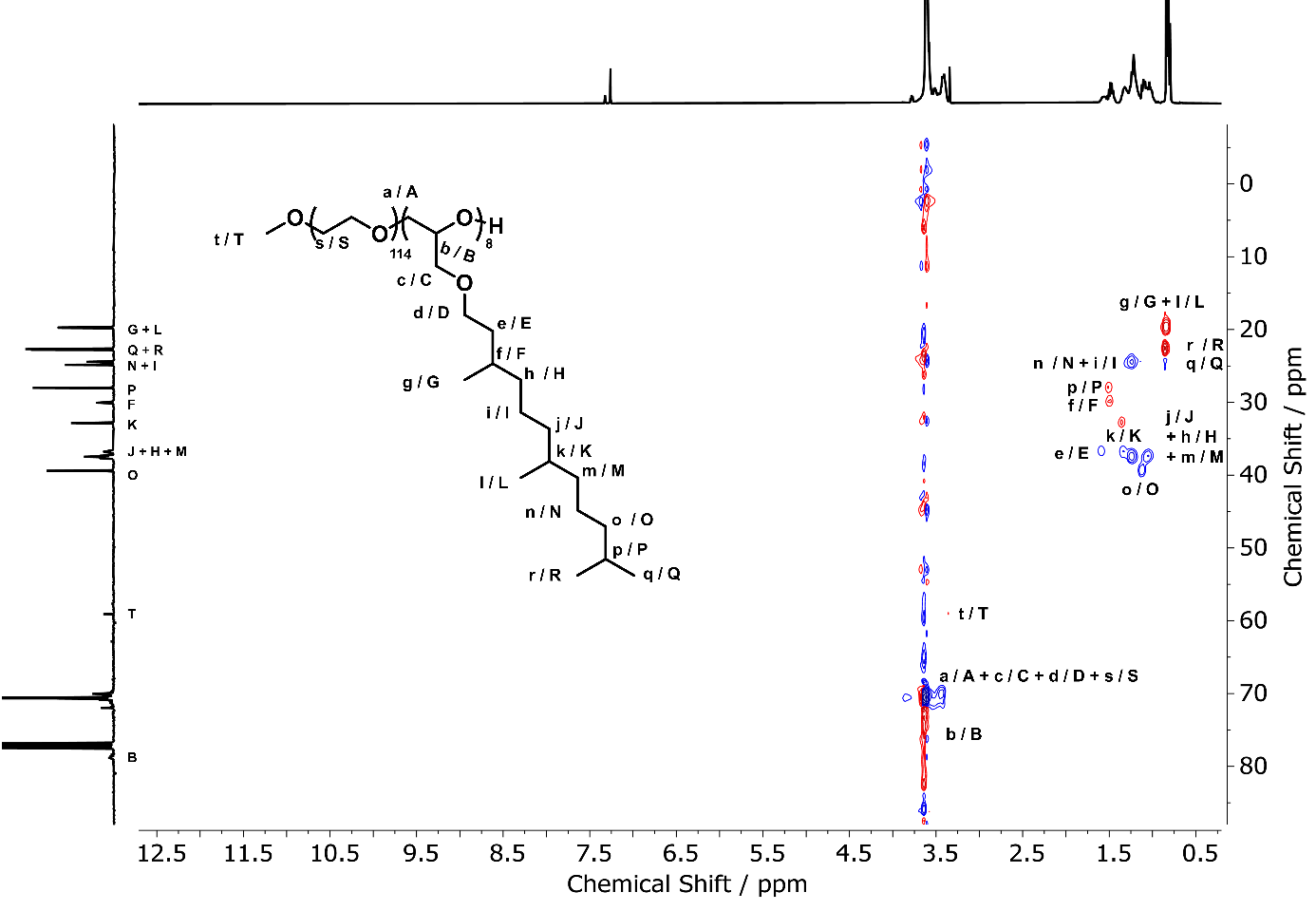


Figure S14: ^1^H-^13^C HSQC NMR spectrum (400 MHz/100 MHz, CDCl_3_) of mPEG_114_-*b*-PHHFarGE_8_.

**
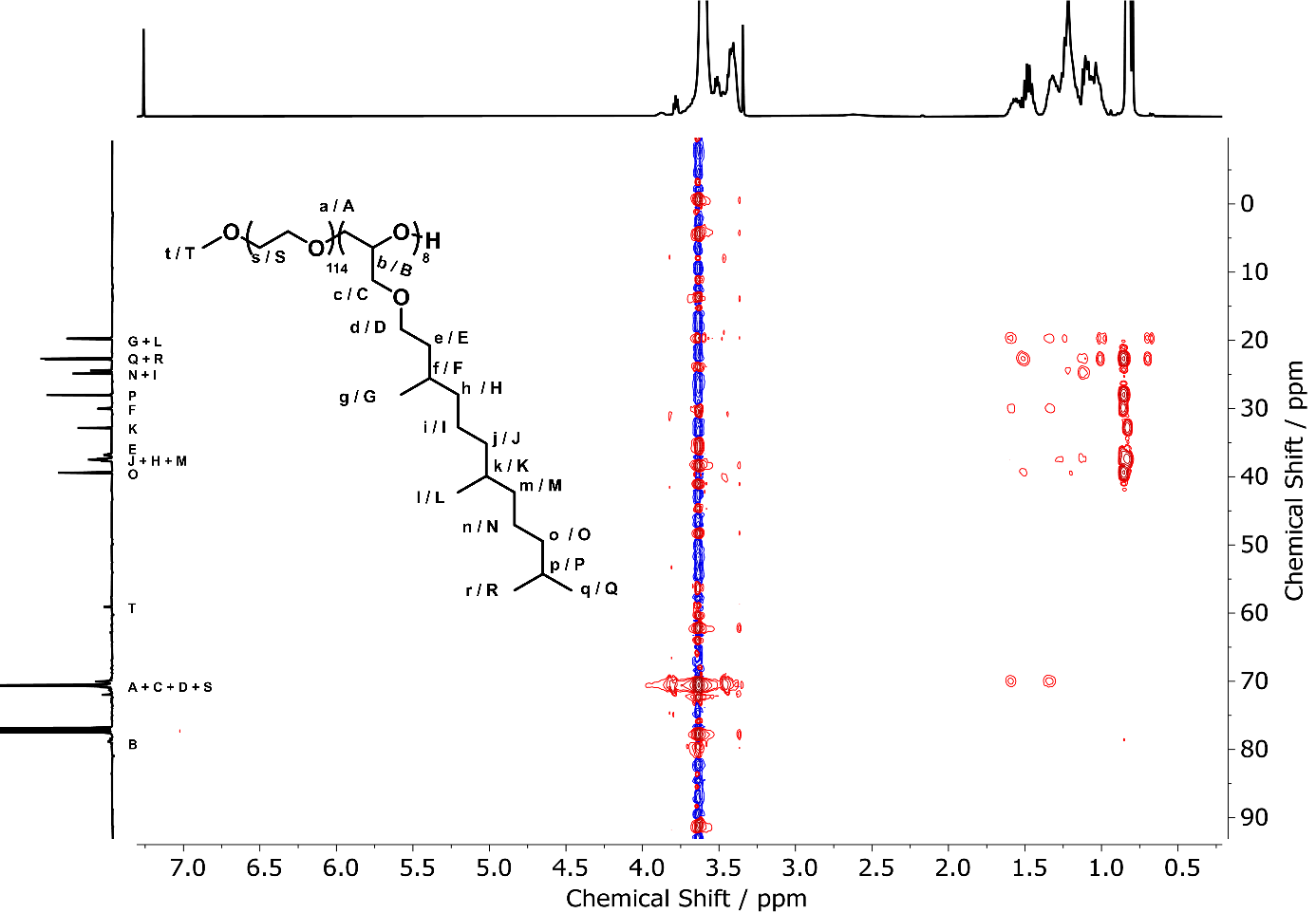
**

Figure S15: ^1^H-^13^C HMBC NMR spectrum (400 MHz/ 100 MHz, CDCl_3_) of mPEG_114_-*b*-PHHFarGE_8_. Coupling assignment was omitted for clarity reasons. Compare Figure S10.


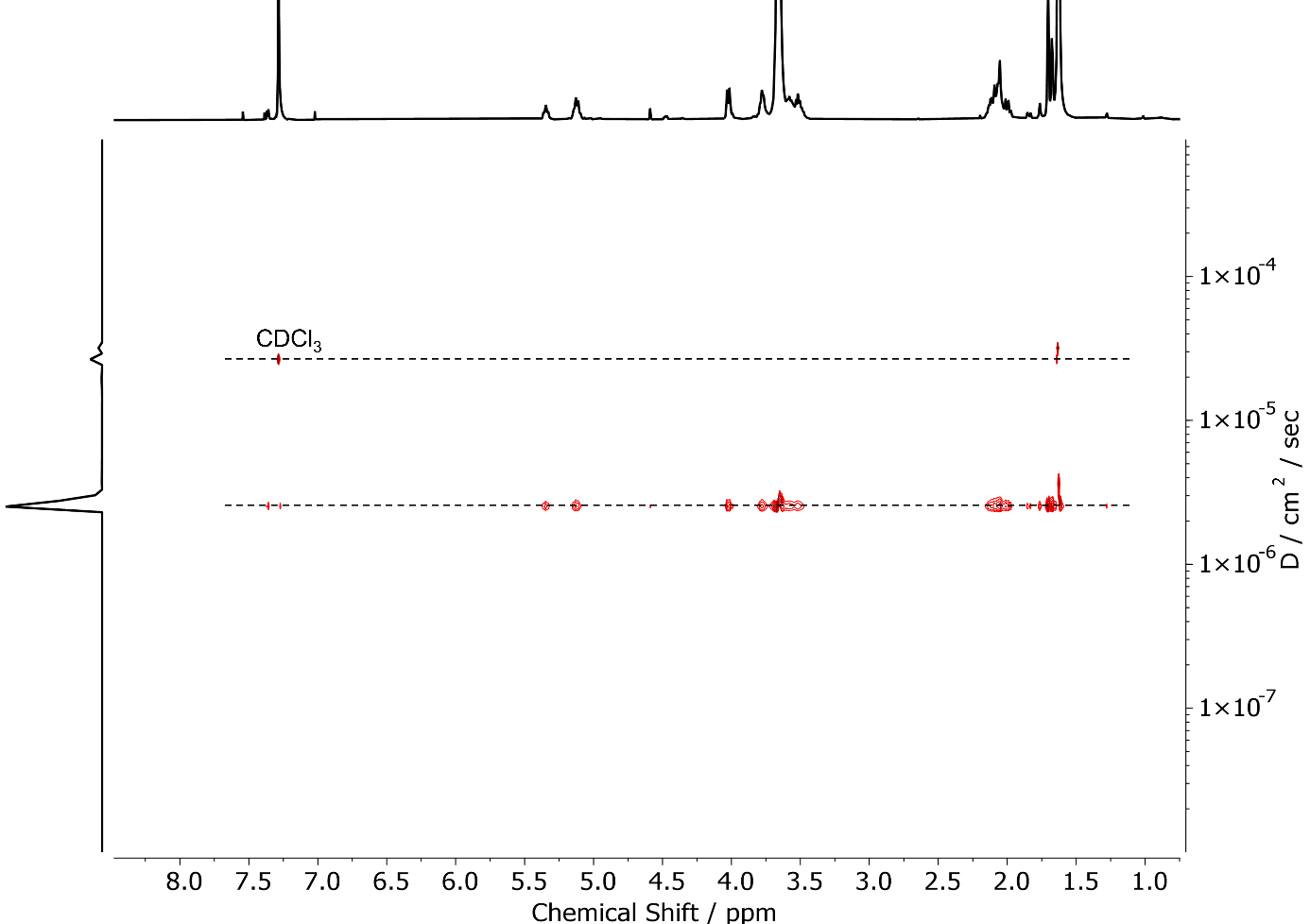


Figure S16: DOSY NMR spectrum (400 MHz MHz, CDCl_3_) of mPEG_114_-*b*-PHHFarGE_5_, featuring a single diffusion coefficient for the block copolymer. The signal at 7.26 ppm and 1.60 ppm with a higher diffusion coefficient belong to the CDCl_3_ solvent and water, respectively.

1. NMR Characterization of PHHFarGE_m_


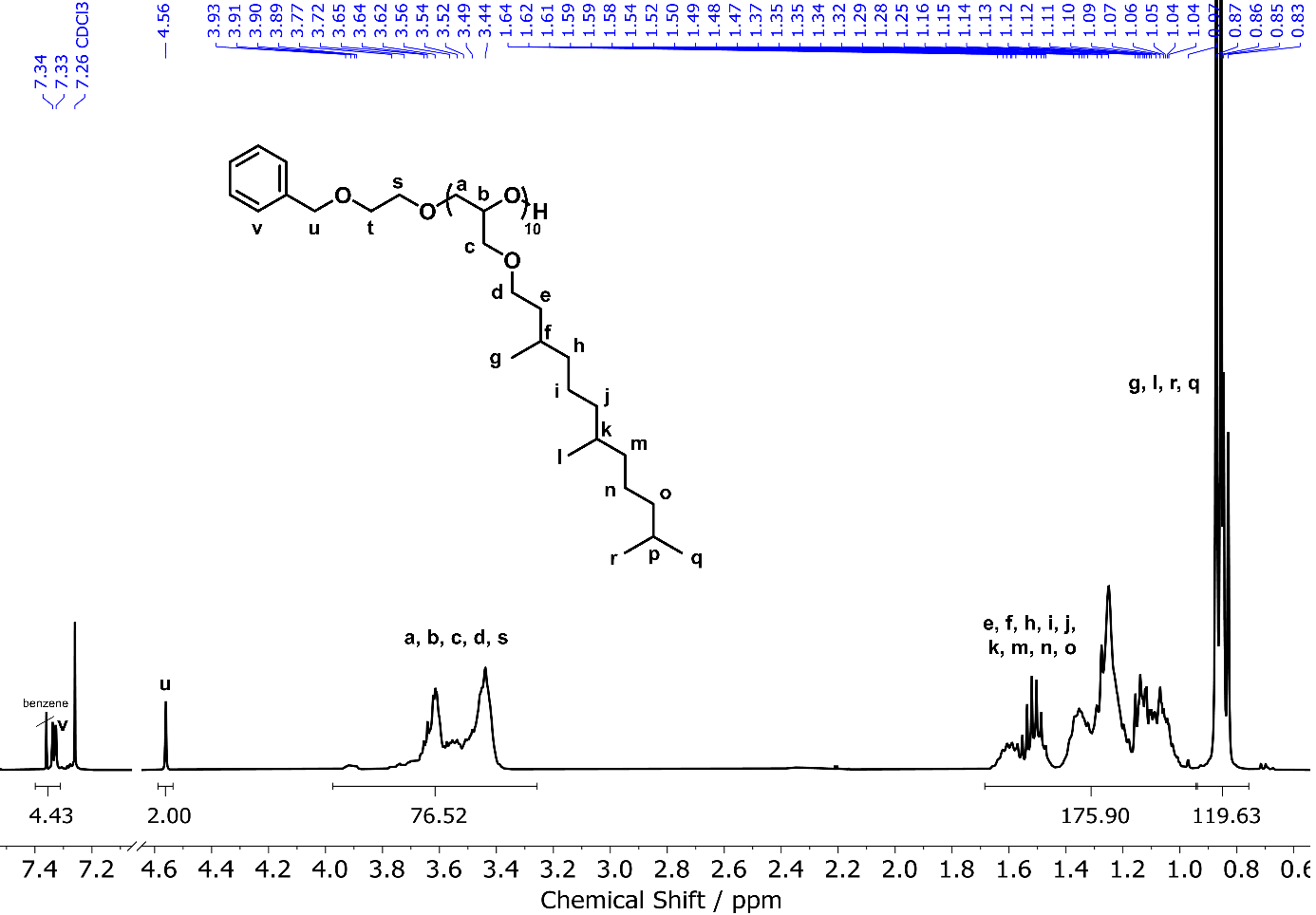


Figure S17: ^1^H NMR spectrum (400 MHz, CDCl_3_) of PHHFarGE_10_.


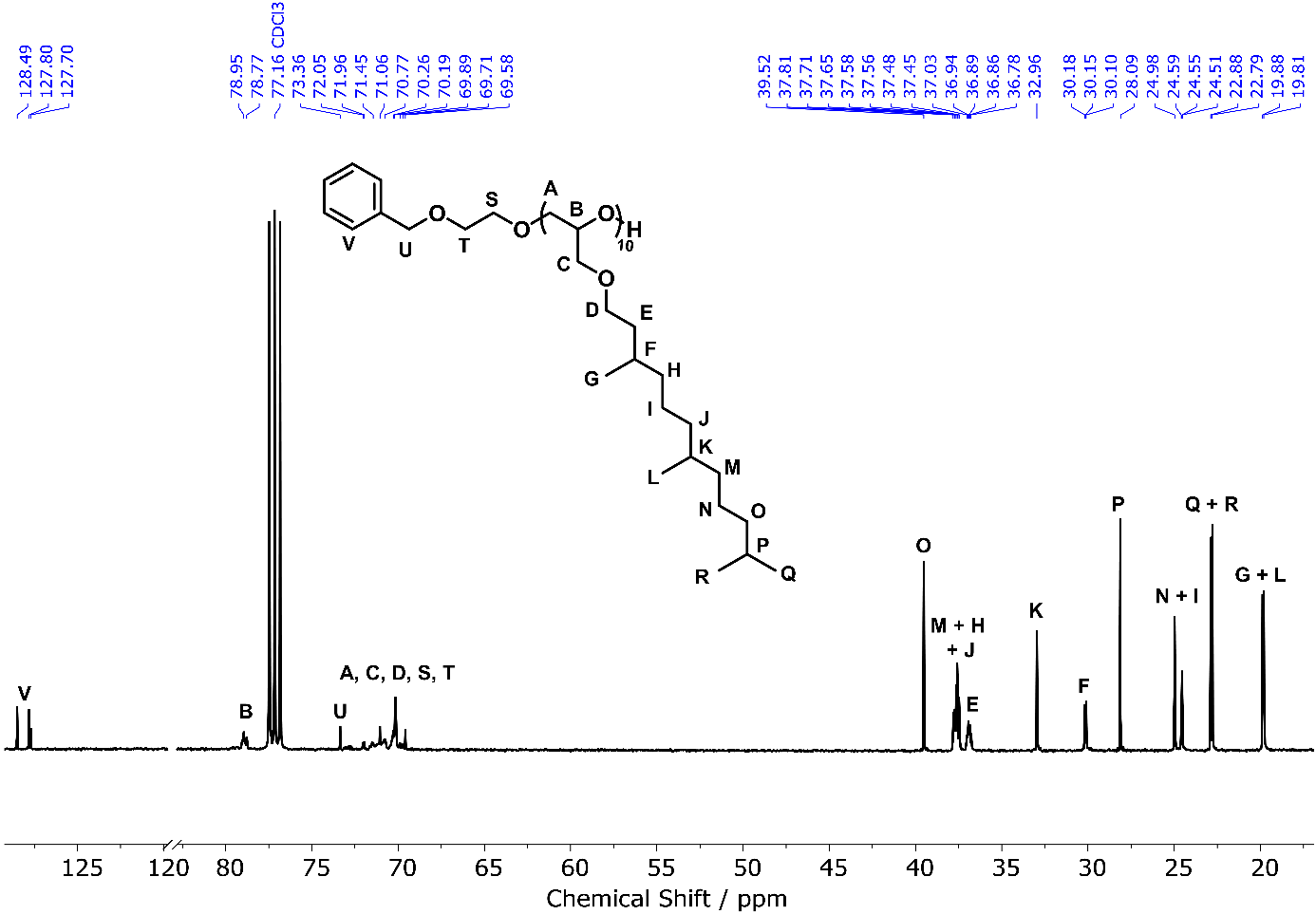


Figure S18: ^13^C NMR spectrum (100 MHz, CDCl_3_) of PHHFarGE_10_.


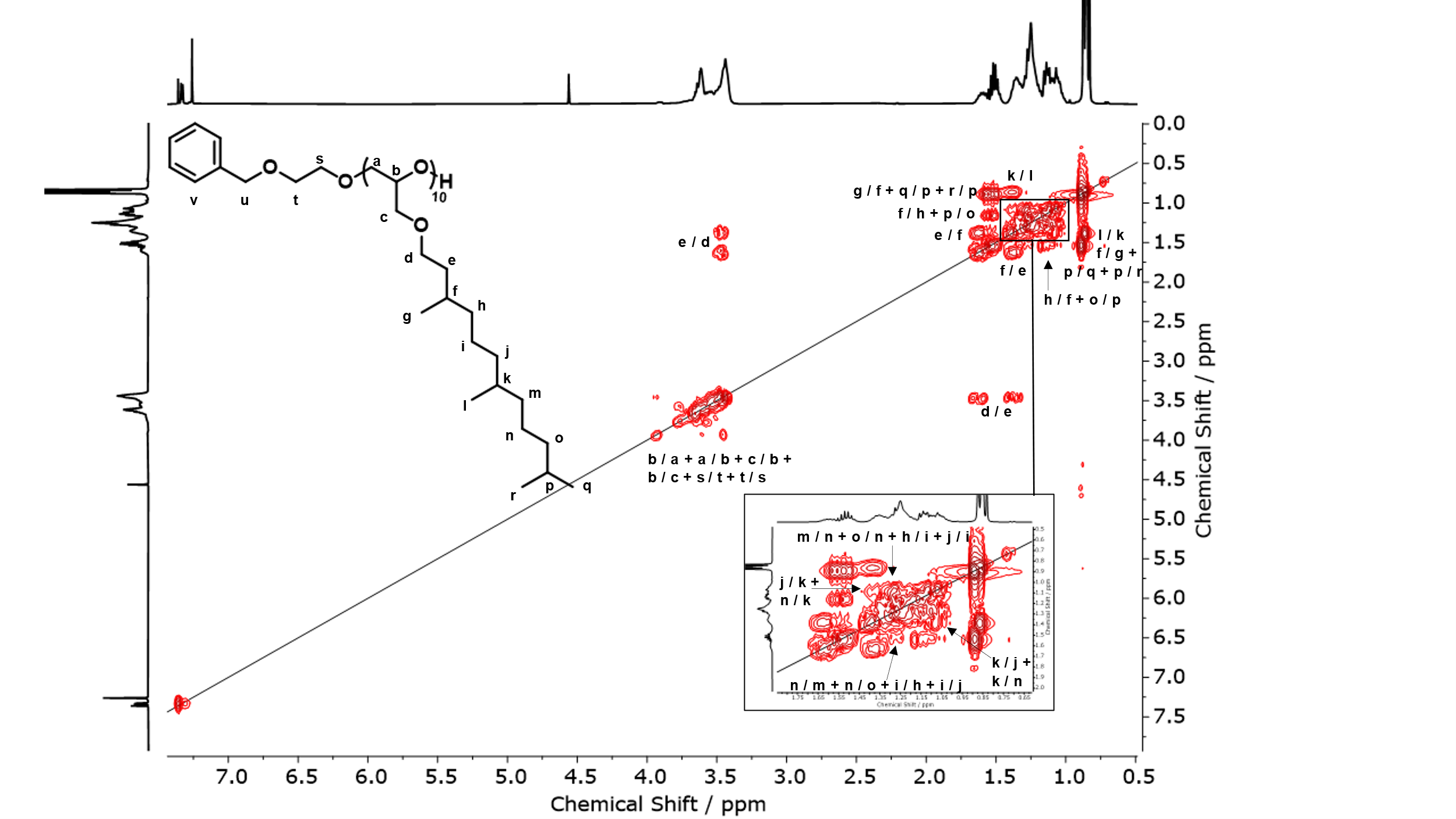


Figure S19: ^1^H-^1^H COSY NMR spectrum (400 MHz, CDCl_3_) of PHHFarGE_10_.


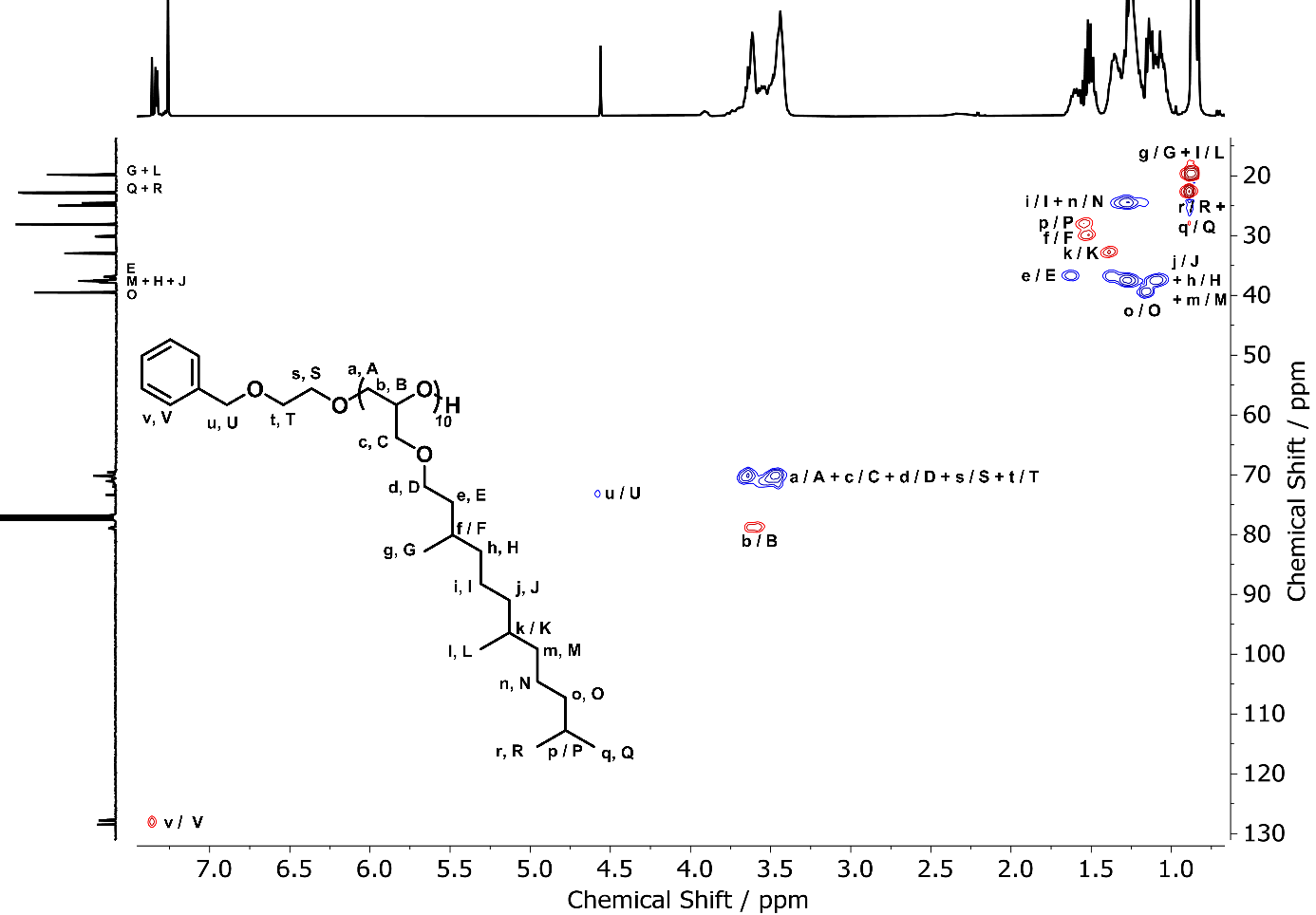


Figure S20: ^1^H-^13^C HSQC NMR spectrum (400 MHz/100 MHz, CDCl_3_) of PHHFarGE_10_.


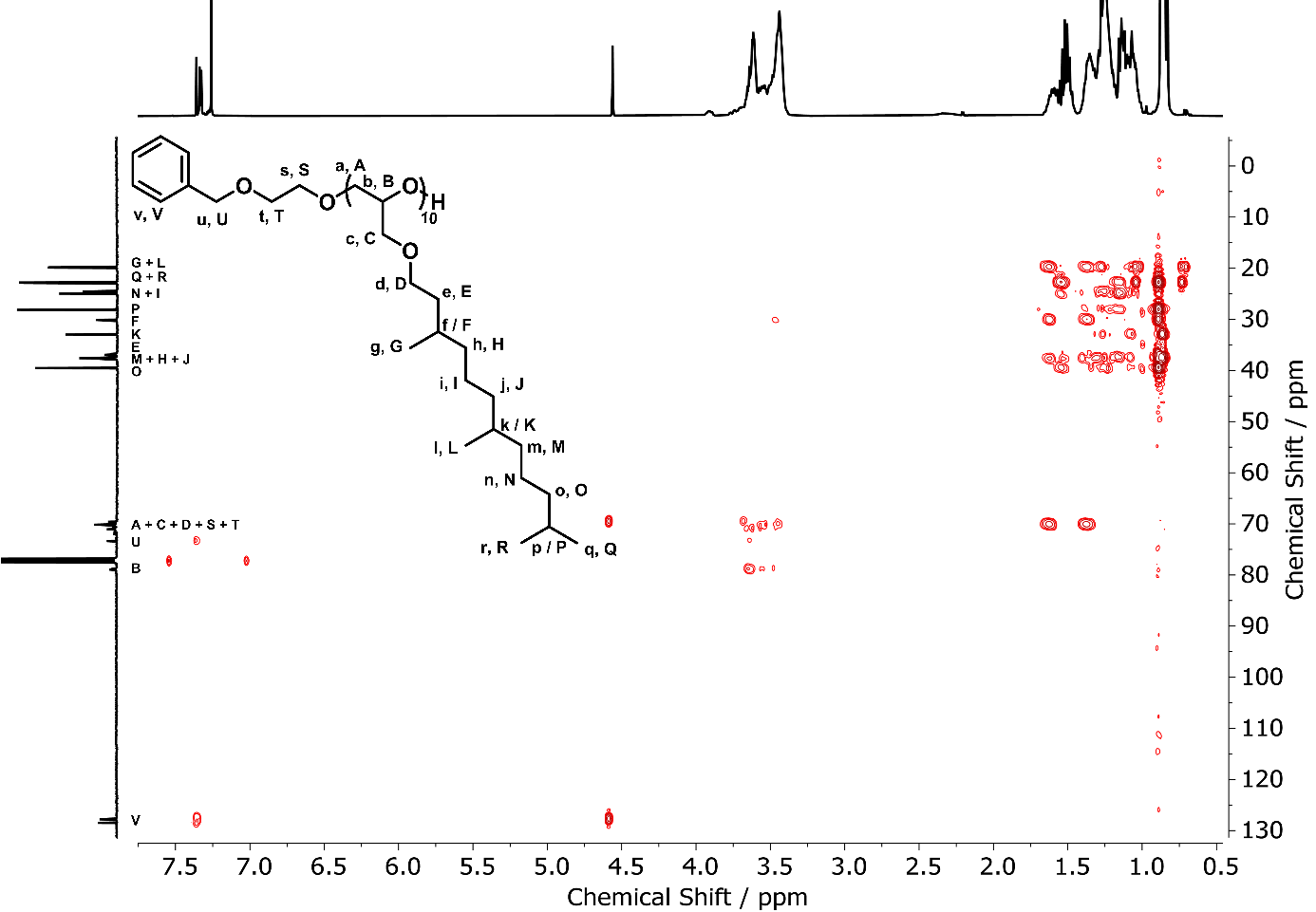


Figure S21: ^1^H-^13^C HMBC NMR spectrum (400 MHz/ 100 MHz, CDCl_3_) of PHHFarGE_10_. Coupling assignment was omitted for clarity reasons. Compare Figure S10.

1. Characterization of PHHFarGE_m_ and PFarGE_m_ Homopolymers

Table S1 summarizes characterization data for homopolymers of HHFarGE and FarGE, which are to be investigated via SANS measurements. Due to the high hydrophobicity of HHFarGE_m_, SEC analysis was conducted in THF as eluent (Figure S22). Owing to the SEC resolution limits, the SEC traces of the homopolymers with a *P*_n_=5 (Table S1, entry 1 and 3) feature several humps towards higher elution volumes (smaller molecular weights), which can be ascribed to the discrete oligomeric species.

Table S1: Characterization data of synthesized PHHFarGE_m_ and PFarGE_m_ homopolymers with respect to molecular weights, dispersity and thermal properties.

| **Entry** | **Sample** | ***M*_n_^a^/ g**∙**mol^-1^** | ***M*_n_^b^ / g**∙**mol^-1^** | ***Ð*^b^** | ***T*_g_^c^ / °C** |
| --- | --- | --- | --- | --- | --- |
| 1 | PHHFarGE_5_ | 1580 | 1800 | 1.12 | -73 |
| 2 | PHHFarGE_10_ | 3000 | 3300 | 1.12 | -74 |
| 3 | PFarGE_5_ | 1540 | 1800 | 1.13 | -74 |
| 4 | PFarGE_12_ | 3490 | 3500 | 1.20 | -75 |

^a^Number-averaged molecular weight determined via ^1^H NMR spectroscopy. ^b^Determined via SEC measurements using THF as eluent and PEG as standard. ^c^Glass transition temperature determined by DSC at a heating rate of 10 °C/min. Extrapolation for entry 1 and 3 was limited due to the lower limit of the measurement. Errors are estimated as ± 2 °C.


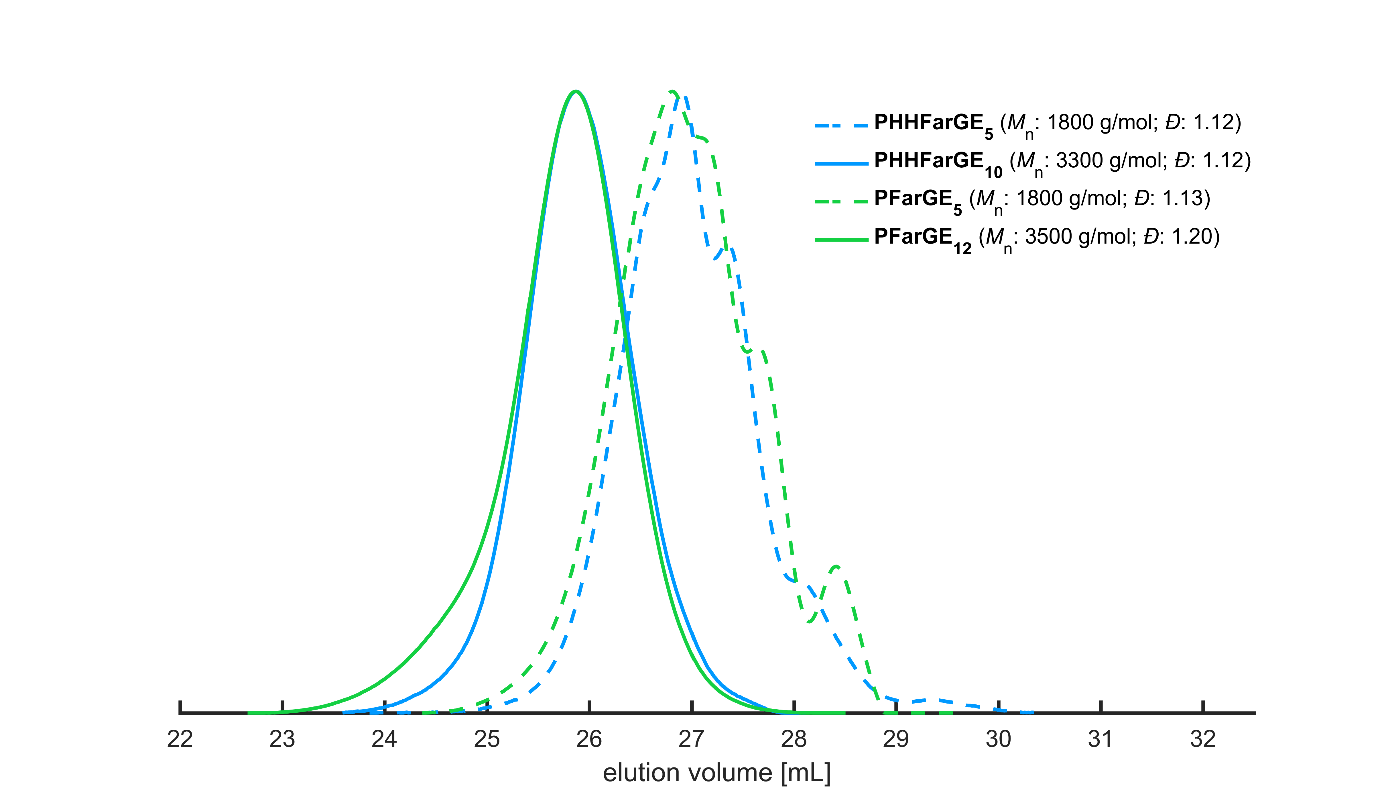


Figure S22: SEC elution traces of the synthesized PHHFarGE_m_ and PFarGE_m_ homopolymers using BzEtOH as initiator (RI detector, eluent: THF, PEG calibration).


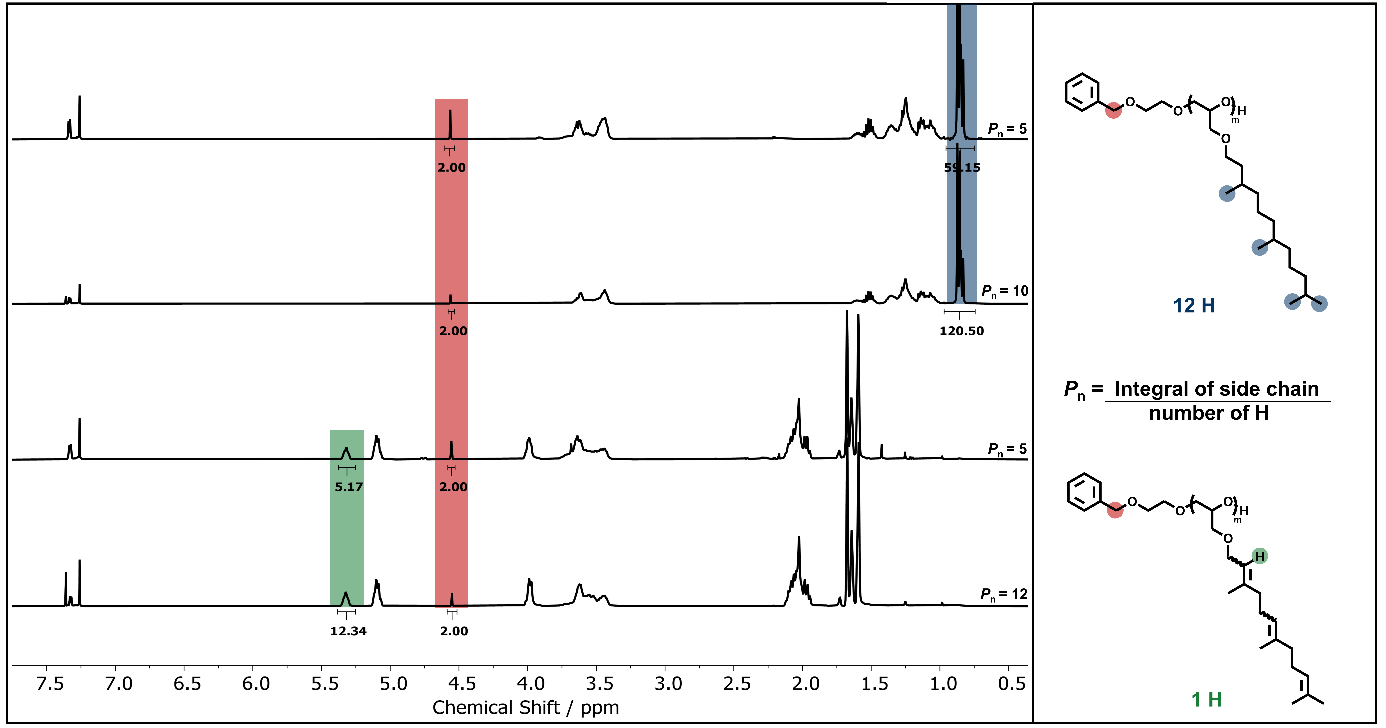


Figure S23: Overlay of ^1^H NMR spectra (400 MHz, CDCl_3_) of PHHFarGE_m_ and PFarGE_m_ homopolymers.

1. SEC Measurements of Diblock Copolymers of FarGE and mPEG_114_


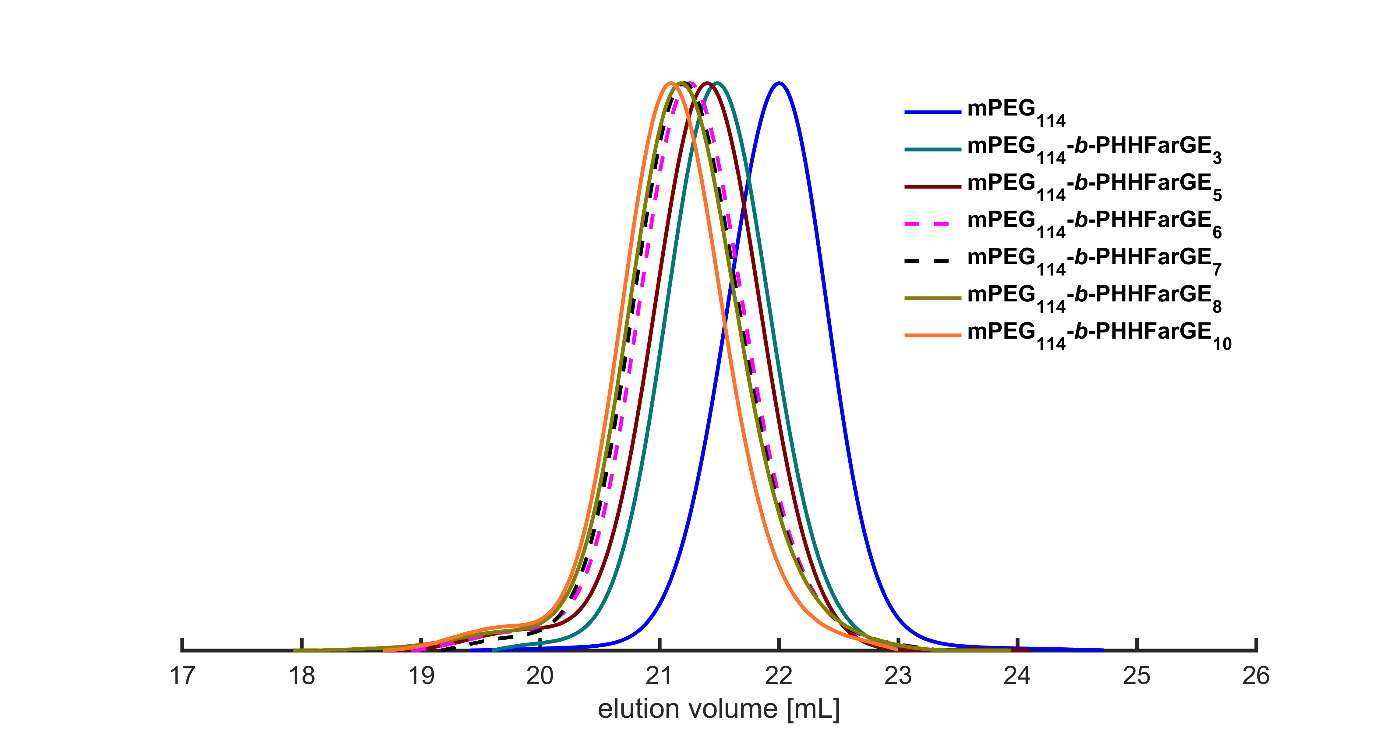


Figure S24: SEC elution traces of selected mPEG_114_-*b*-PHHFarGE_m_ diblock copolymers compared to the mPEG_114_ macroinitiator (RI detector, eluent: DMF, calibration: PEG standard).


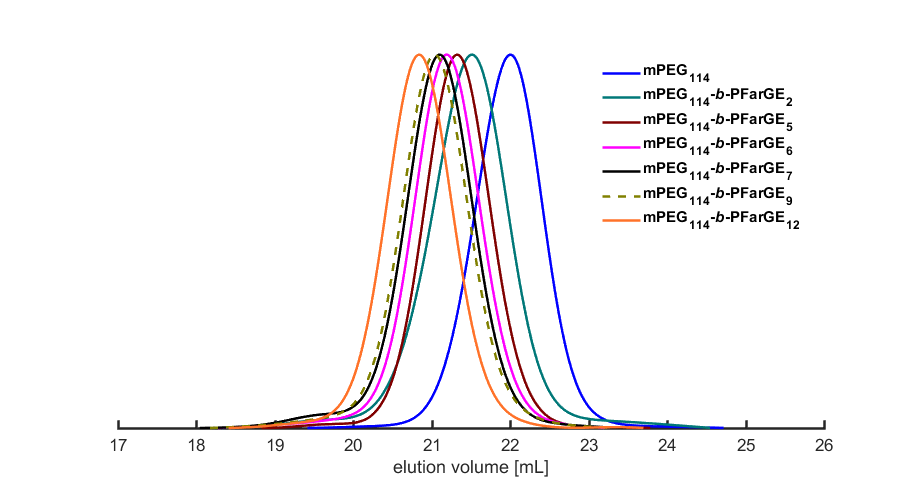


Figure S25: SEC elution traces of selected mPEG_114_-*b*-PFarGE_m_ diblock copolymers compared to initial the mPEG_114_ macroinitiator (top) (RI detector, eluent: DMF, PEG calibration).^18^

1. DSC Thermograms of the Synthesized Diblock Copolymers and Homopolymers

All results of the thermal analysis are given in Table 1.


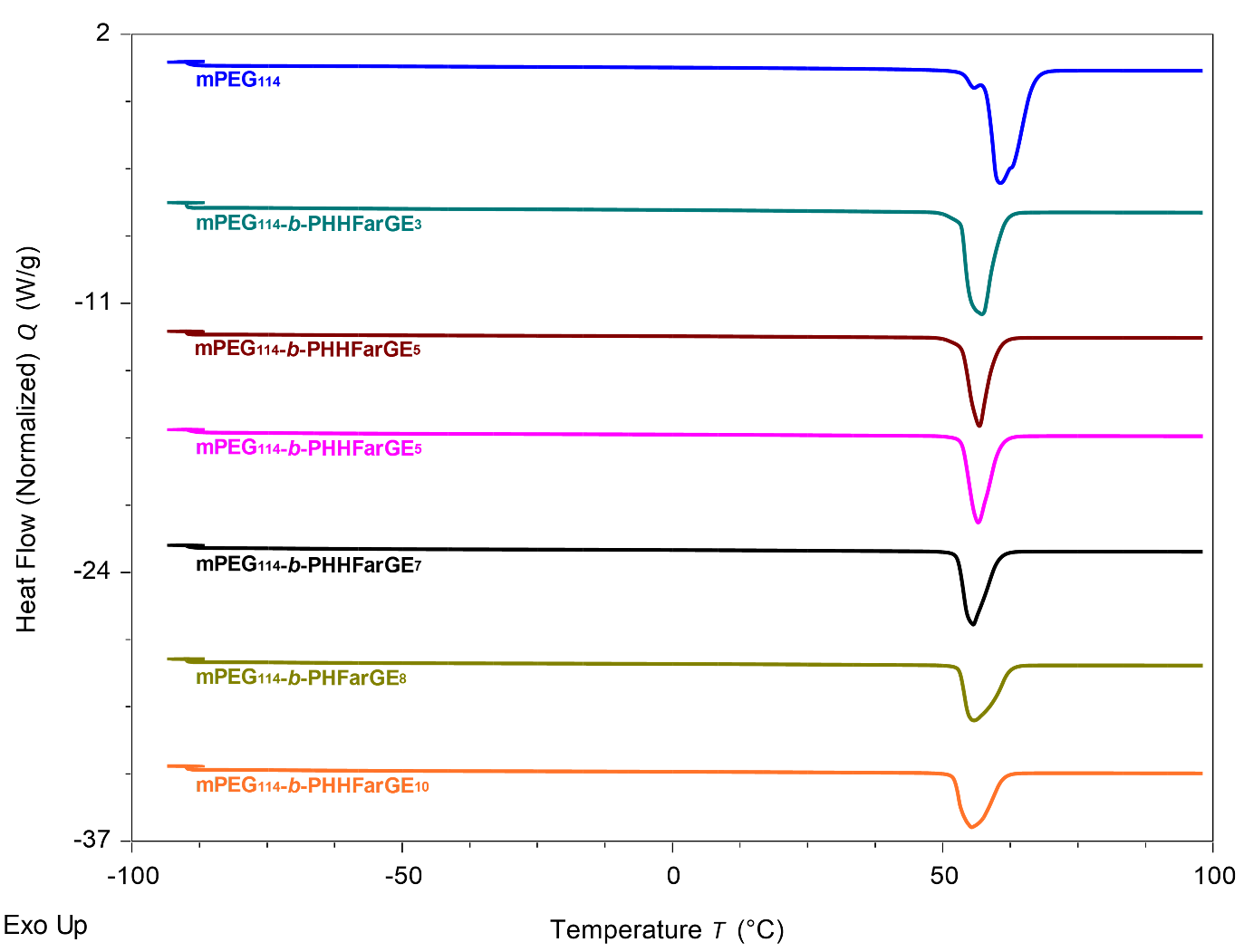


Figure S26: DSC thermograms of mPEG_114_-*b*-PHHFarGE_m_ diblock copolymers (second heating cycle, from -90 to 100 °C, 10 °C·min^-1^).


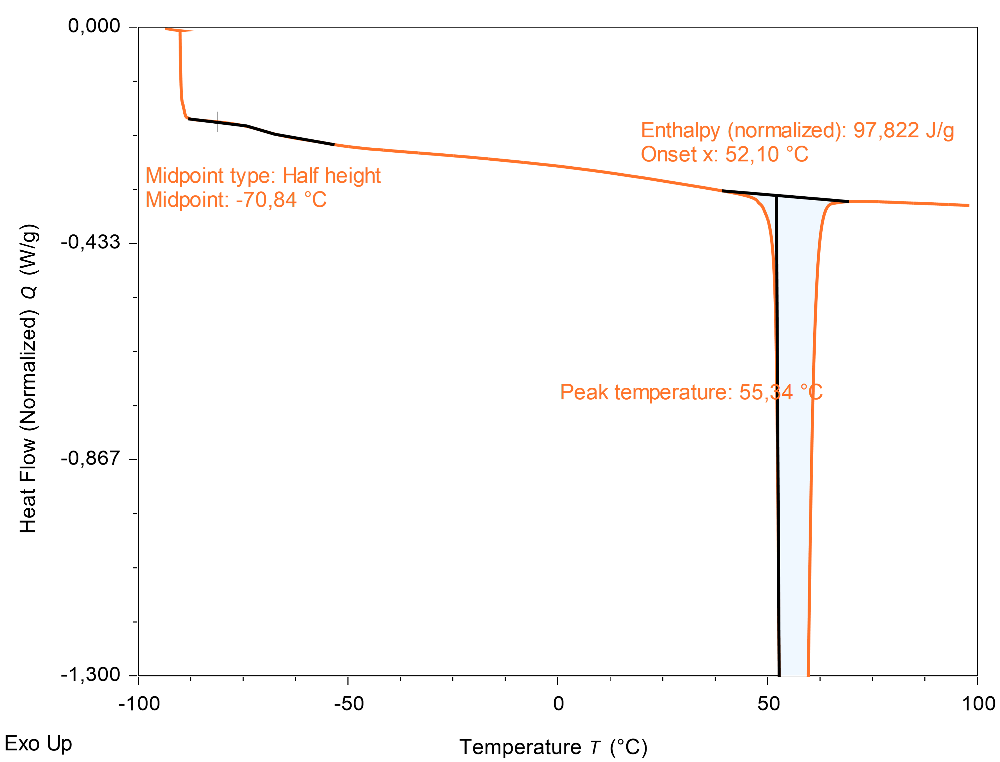


Figure S27: Exemplary DSC thermogram of mPEG_114_-*b*-PHHFarGE_10_ diblock copolymer exhibiting a weak glass transition feature (second heating cycle, from -90 to 100 °C, 10 °C·min^‑1^).


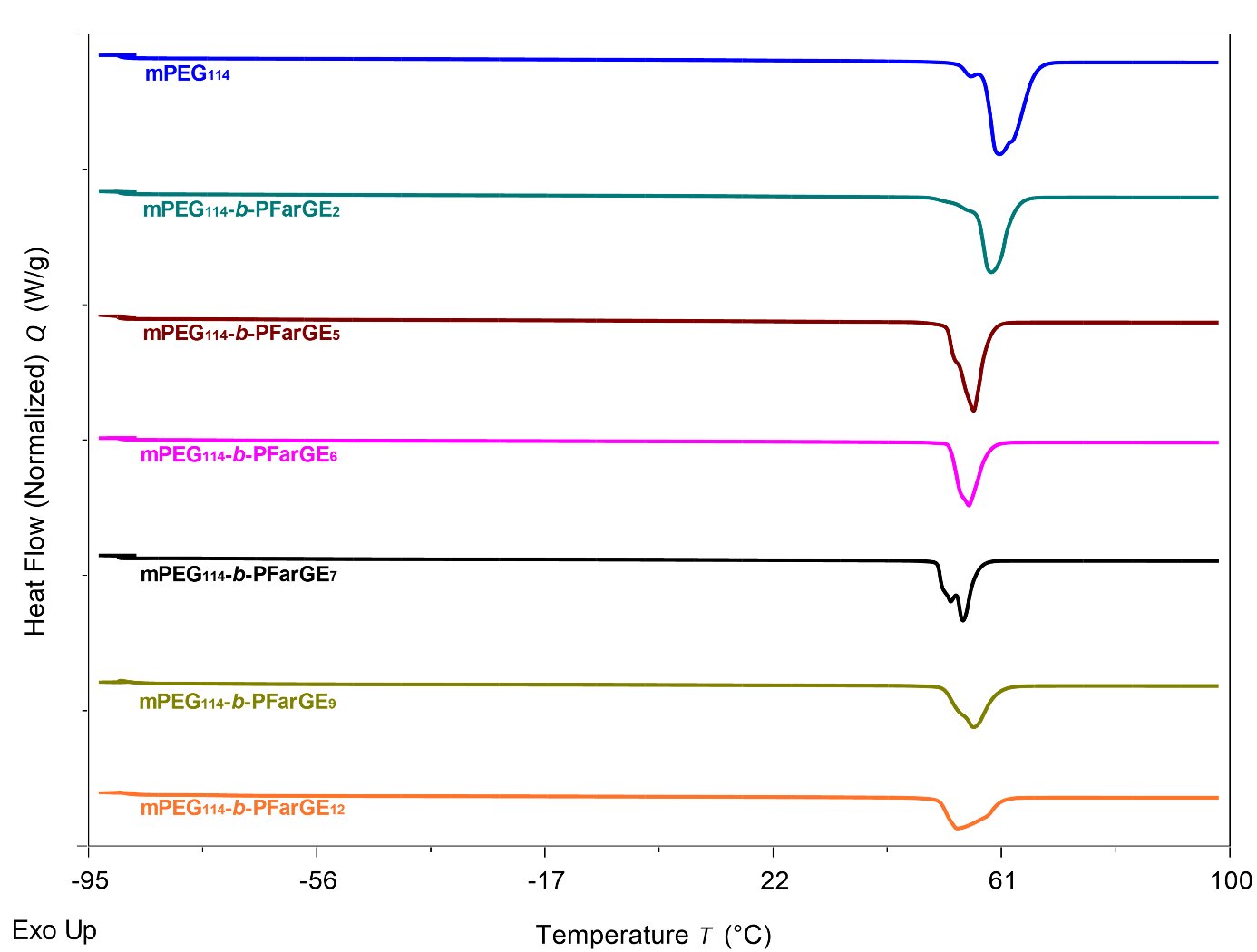


Figure S28: DSC thermograms of mPEG_114_-*b*-PFarGE_m_ diblock copolymers (second heating cycle, from -90 to 100 °C, 10 °C·min^-1^). Thermal analyses have been previously reported.^18^

**
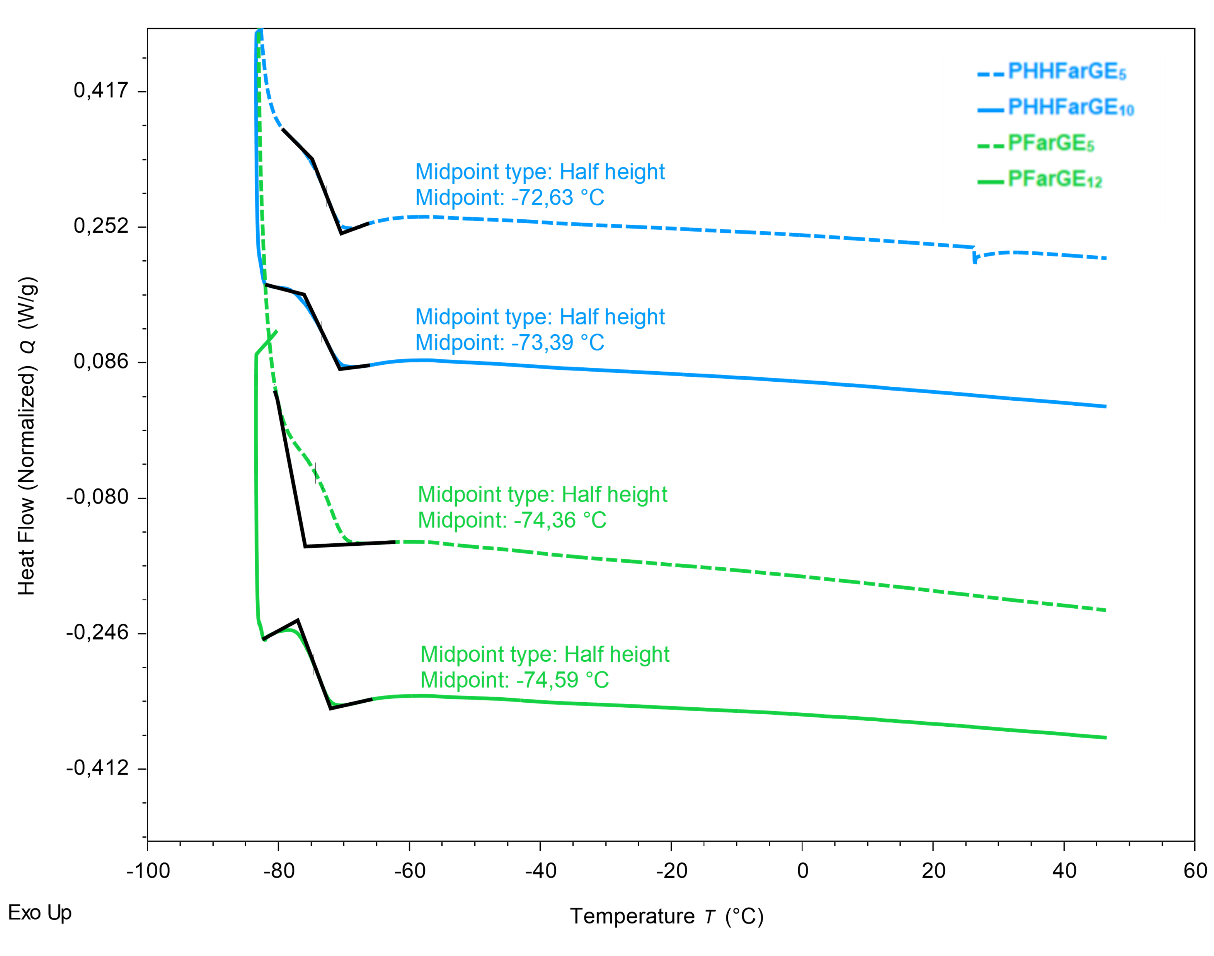
**

Figure S29: DSC thermograms of PHHFarGE_x_ and PFarGE_x_ homopolymers (second heating cycle, from -90 to 50 °C, 10 °C·min^‑1^). Extrapolation for PHHFarGE_5_ and PFarGE_5_ was limited due to the lower limit of the measurement. Errors are estimated as ± 2 °C.

1. Determination of CMCs via Fluorescence Spectroscopy (FS)

We previously reported on a power law equation suitable for very low amphiphile concentrations, allowing for the determination of the diblock copolymer CMC:^18^

| *I*_1_/*I*_3_ $=\frac{A_{1}-A_{2}}{1+{(\frac{c}{c_{0}})}^{f}}+$ $A_{2},$ | Eq. 1 |
| --- | --- |

$A_{1}$ and $A_{2}$ are the upper and lower limits of the sigmoid (*I*_1_/*I*_3_ for *c* → 0 and *c* → $\infty$, respectively), $f$ represents the steepness of the sigmoid and $c_{0}$ is the concentration at the inflection point of the sigmoid and equal to the CMC. The advantage of this altered version of the exponential-based approach by Aguiar et al. is its applicability to determine solely positive concentrations and lower $\chi^{2}$.^5^

FS data for all FarGE (Figure S30) and HHFarGE polymers (Figure S31) are shown below. The hydrophobicity of the pyrene surroundings influences the fluorescence intensity ratio *I*_1_/*I*_3_. However, the *I*_1_/*I*_3_ ratio approaches a value of *I*_1_/*I*_3_ ≈ 1.2 for all diblock copolymers of varying length as the hydrophobic core always consists of FarGE and HHFarGE, which are similar in their side chain structure. The dependence of the CMC on copolymer hydrophobicity is presented in Figures S32 and S33. All CMC values are summarized in Table 2 of the main manuscript.


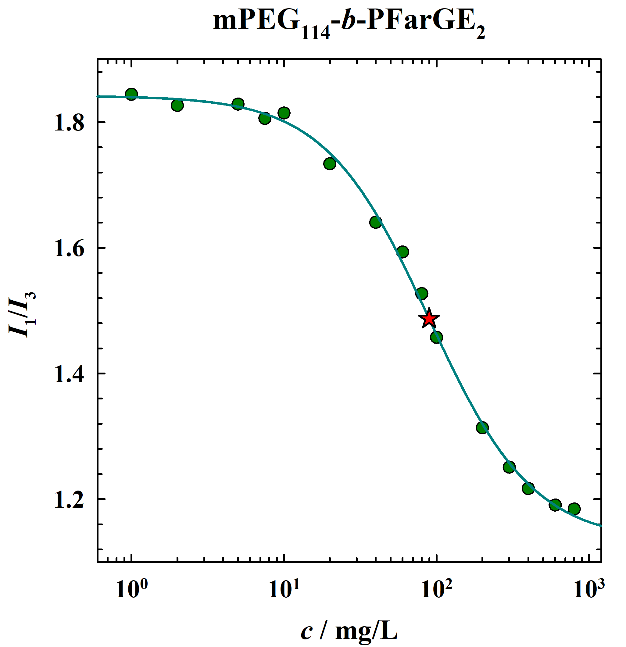

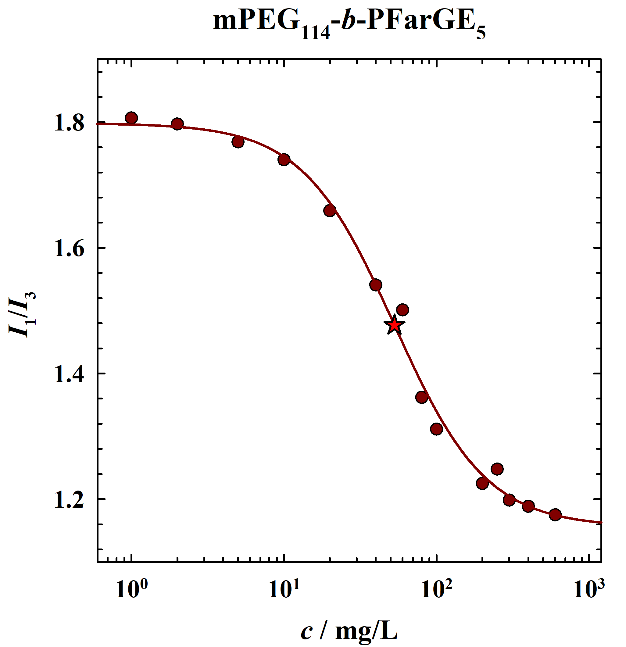

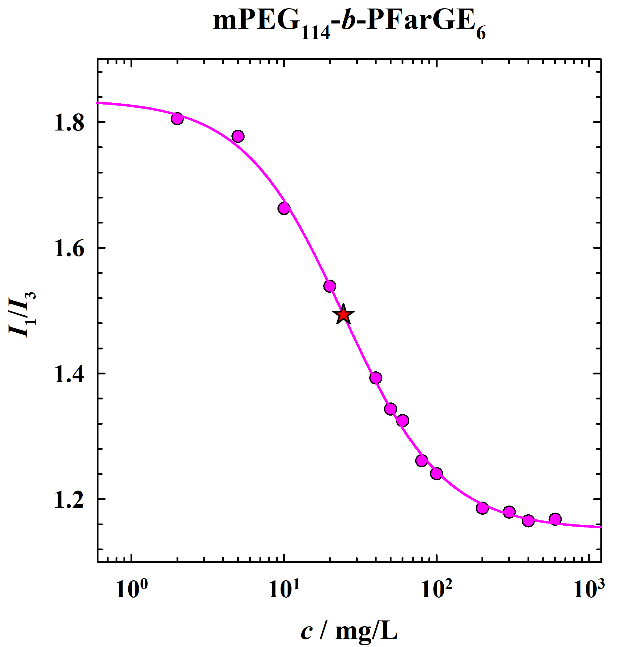

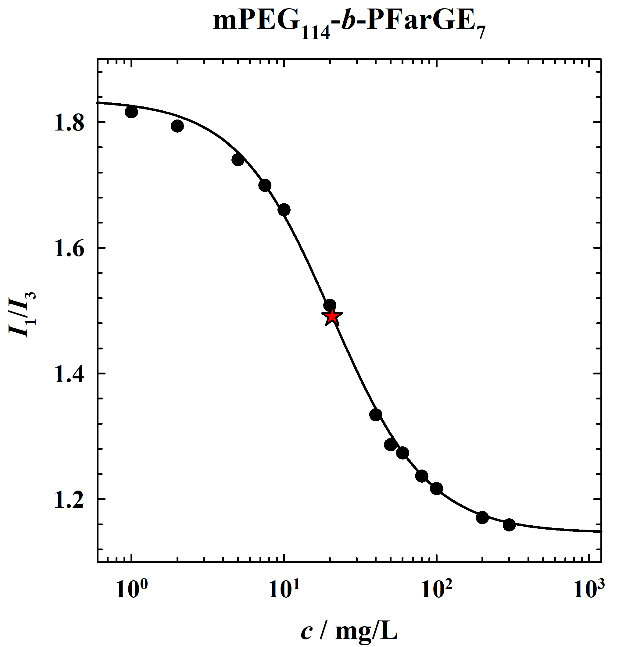

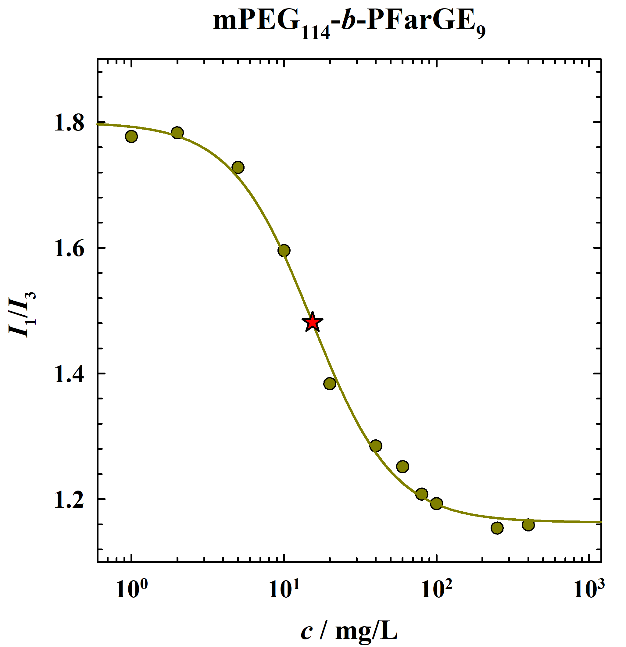

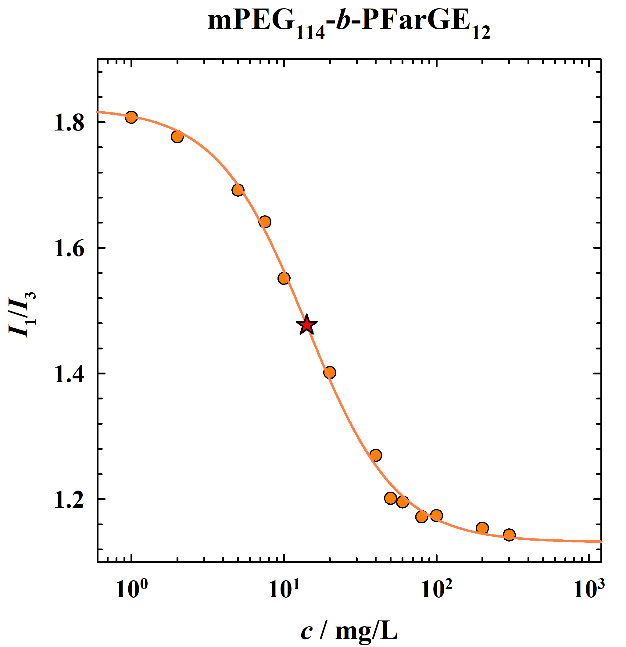


**Figure** S30: Plots of the pyrene intensity ratio *I*_1_/*I*_3_ (*c*_pyrene_ = 7·10^-7^ M) versus polymer concentration, allowing for determination of the CMCs via fluorescence spectroscopy for the series of mPEG_114_-*b*-PFarGE_m_ diblock copolymers. CMCs are depicted with stars. Data for mPEG_114_-*b*-PFarGE_5_ and mPEG_114_-*b*-PFarGE_9_ are modified from one of our earlier works.^18^


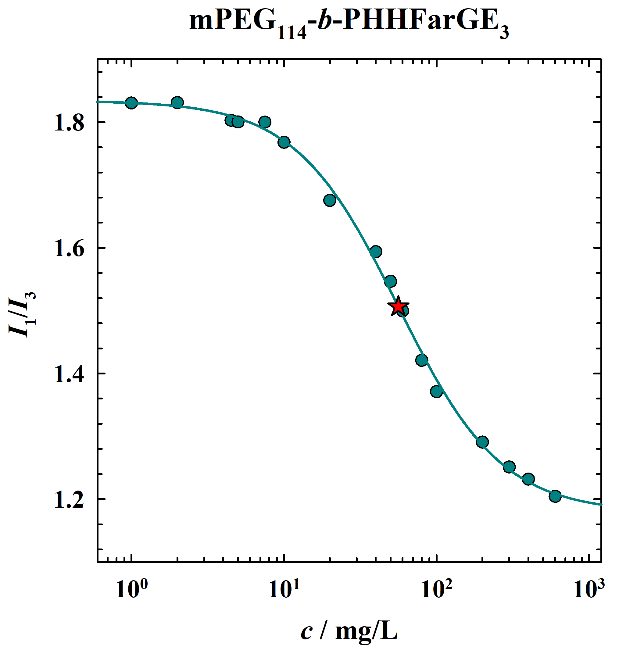

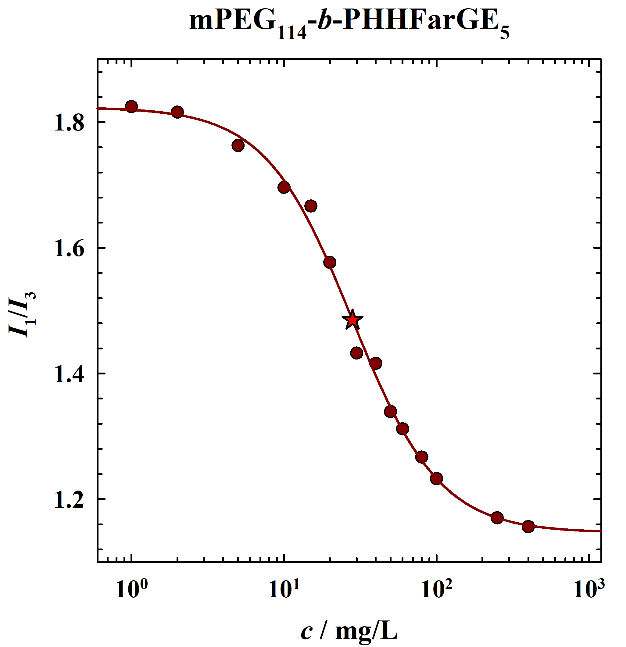

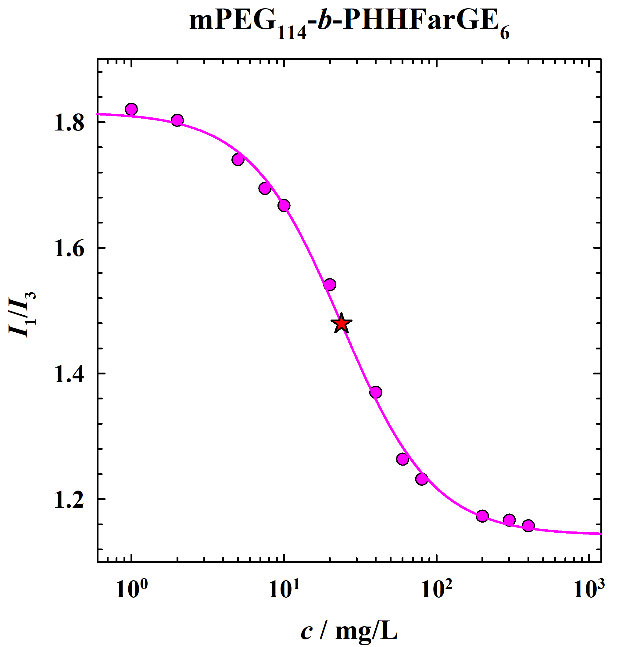

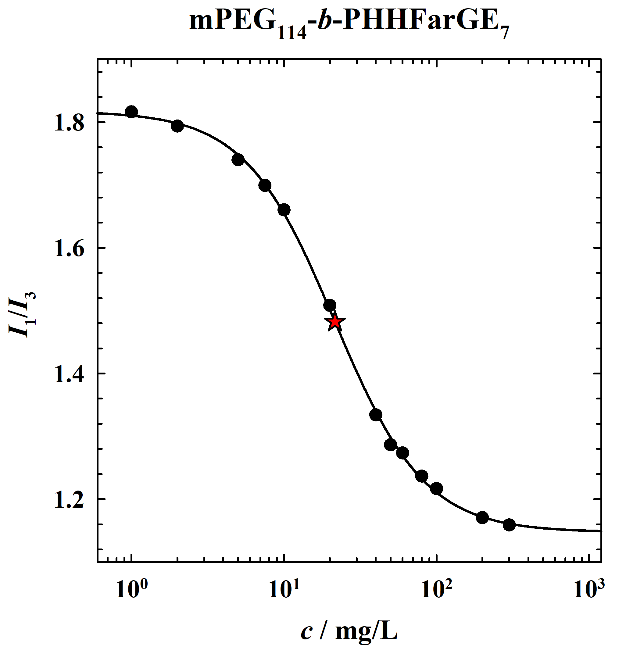

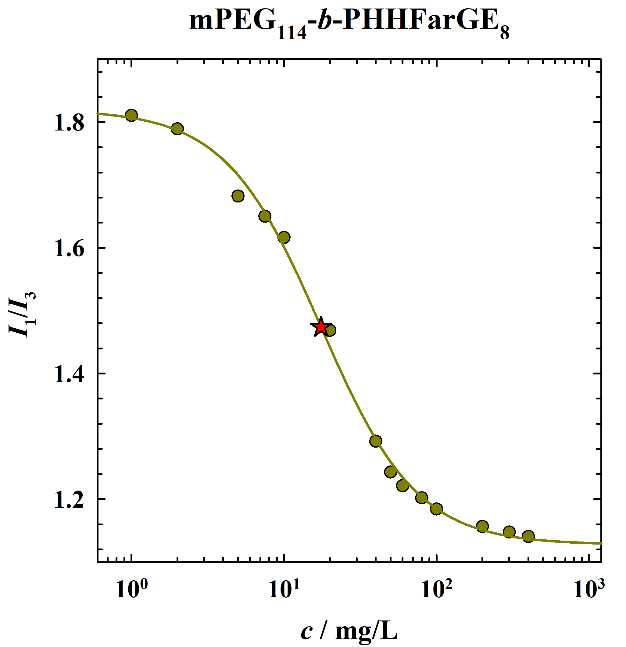

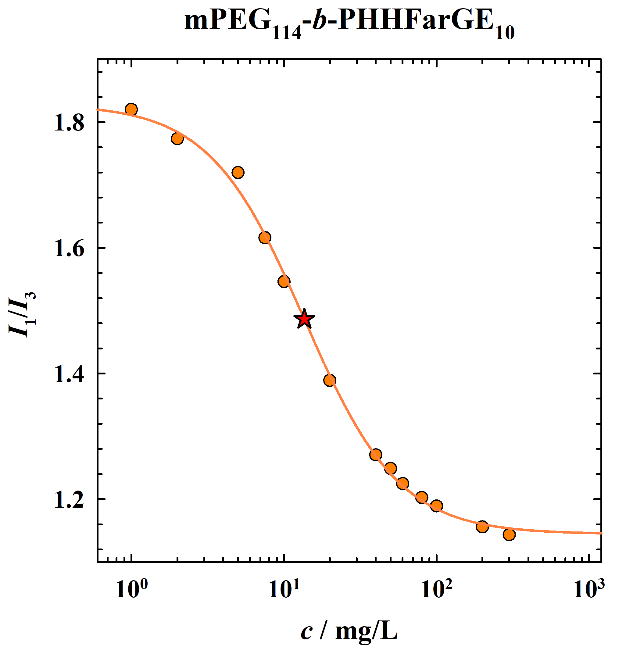


**Figure** S31: Plots of the pyrene intensity ratio *I*_1_/*I*_3_ (*c*_pyrene_ = 7·10^-7^ M) versus polymer concentration, allowing for determination of the CMCs via fluorescence spectroscopy for the series of mPEG_114_-*b*-PHHFarGE_m_ diblock copolymers. CMCs are depicted with stars.


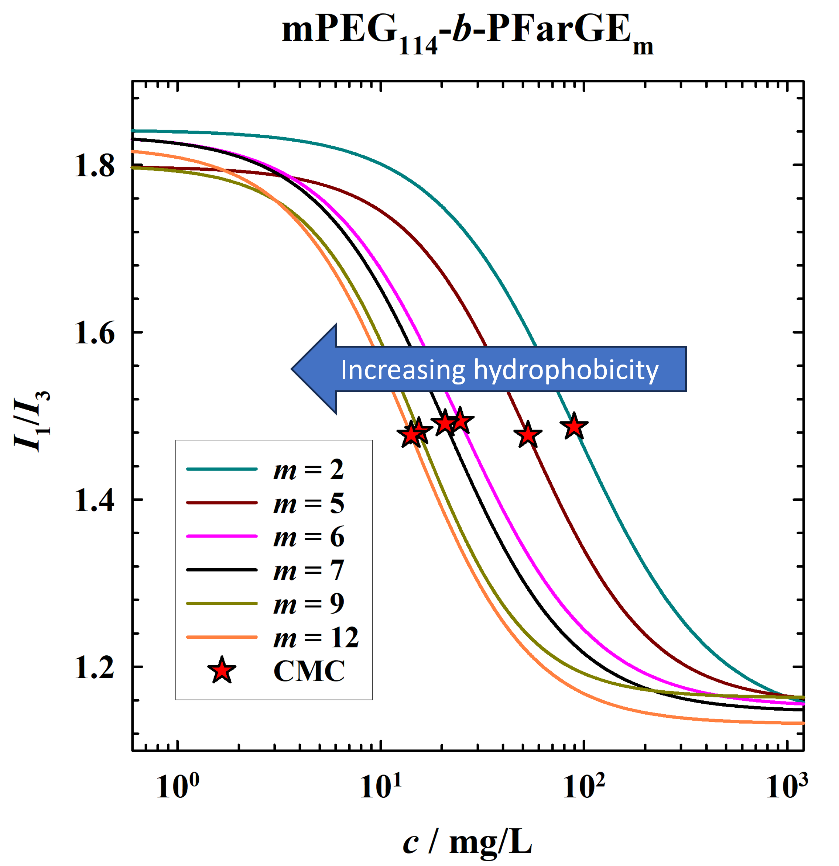


Figure S32: Overlay of intensity ratio *I*_1_/*I*_3_ plots versus diblock copolymer concentration for the mPEG_114_-*b*-PFarGE_m_ diblock copolymer series, visualizing the shift to lower CMC values with increasing FarGE block size. CMCs are depicted with stars. To maintain clarity, only the fitting curves are depicted without the respective data points. Underlying data for mPEG_114_-*b*-PFarGE_5_ and mPEG_114_-*b*-PFarGE_9_ are taken from one of our earlier works, cf. Figure S30.^18^


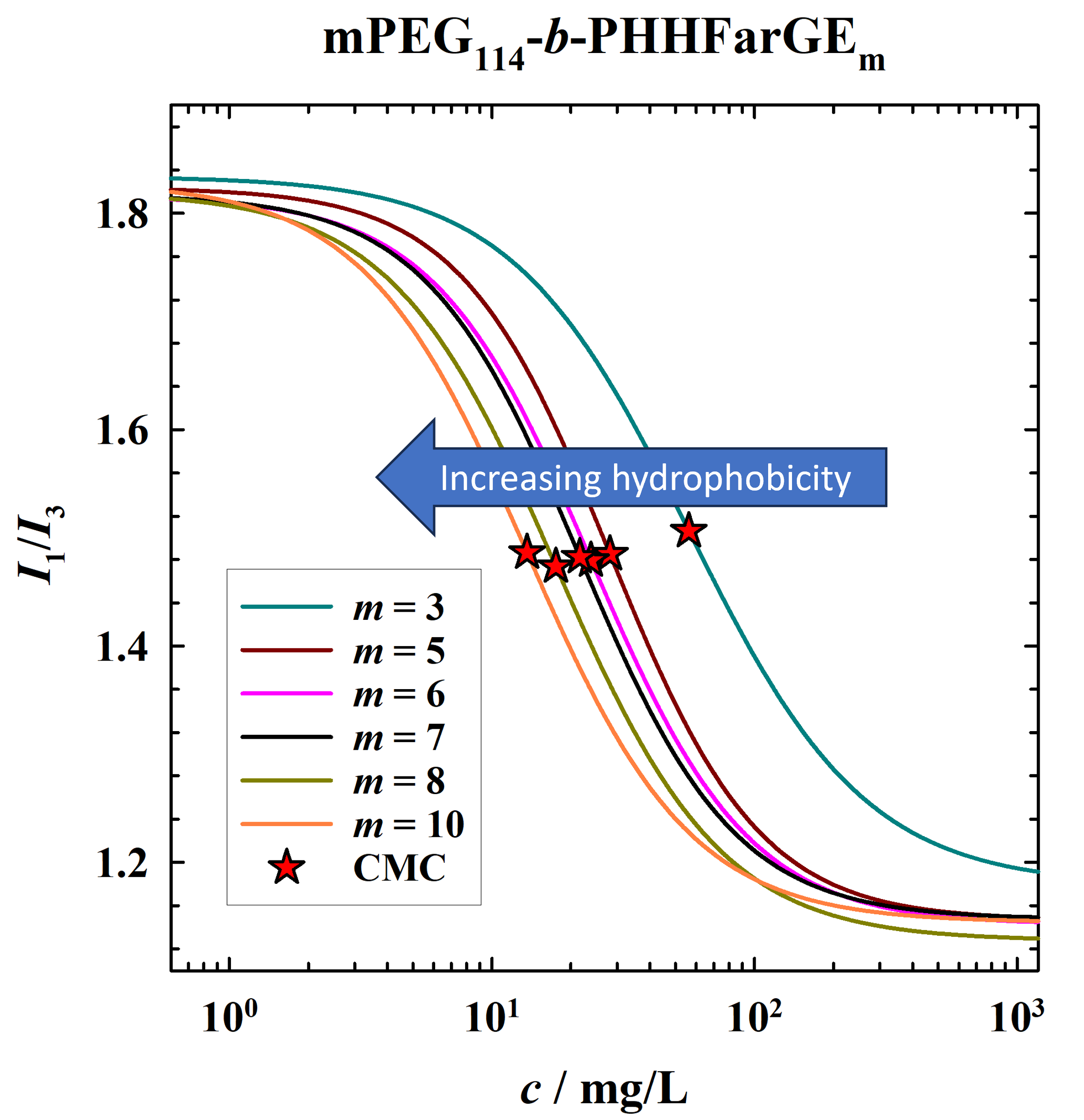


Figure S33: Overlay of intensity ratio *I*_1_/*I*_3_ plots versus diblock copolymer concentration for the mPEG_114_-*b*-PHHFarGE_m_ diblock copolymer series, visualizing the shift to lower CMC values with increasing HHFarGE block size. CMCs are depicted with stars. To maintain clarity, only the fitting curves are depicted without the respective data points.

1. Determination of CMCs via Static and Dynamic Light Scattering (SLS/DLS)

Additional static and dynamic light scattering experiments (SLS/DLS, measurement details can be found above) were performed for some copolymers to verify the results obtained from fluorescence spectroscopy. Results are shown here exemplarily for mPEG_114_-*b*-PHHFarGE_5_ in comparison to the FS data. The concentration-dependent count rate obtained from SLS was converted into the excess Rayleigh ratio *R*_vv_, determined with toluene as reference scatterer, and plotted against the mPEG_114_-*b*-PHHFarGE_5_ concentration *c*. Before micelle formation takes place, only unimers exist in the aqueous medium and the scattering signal is therefore weak (teal line, right). This is in line with noisy and strongly fluctuating intensity-time autocorrelation functions (g^2^($\tau$)) (middle, bottom). Upon exceeding the CMC, the correlation function shows a typical trend with a high intercept, indicating good measurement quality (middle top). Concomitantly, copolymer self-aggregation leads to a much stronger scattering intensity (dark yellow line, right). In agreement with the fluorescence results, a CMC of 24 $\pm$5 mgL^-1^ is obtained (red star).


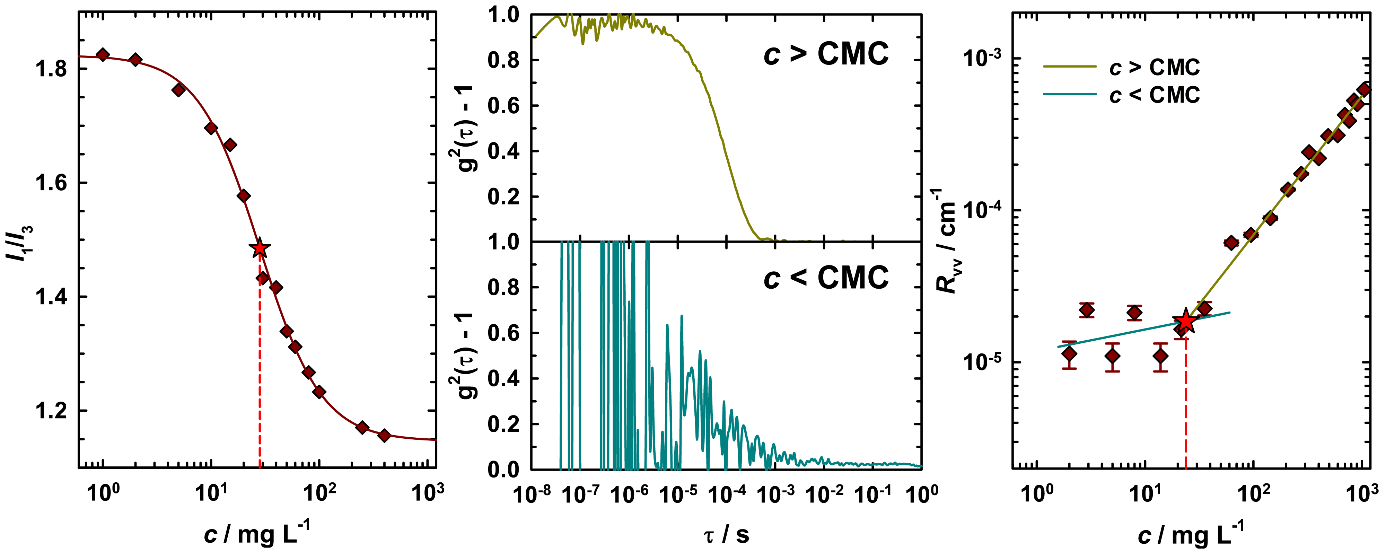


Figure S34: Concentration-dependent micellization of the terpenoid-based polyether mPEG_114_-*b*-PHHFarGE_5_ in water, studied by light scattering and fluorescence spectroscopy measurements at *T* = 23 $\boldsymbol{\pm}$ 1 °C. The CMC is denoted with a red star. Left: Pyrene *I*_1_/*I*_3_ ratio determined via fluorescence spectroscopy vs. polymer concentration *c*., as shown in Figure S30. Middle: Selected DLS correlation functions g^2^($\boldsymbol{\tau}$)-1 vs. lag time $\boldsymbol{\tau}$ for concentrations above (top, green) and below (bottom, gray) the CMC. Right: Excess Rayleigh ratio *R*_vv_ determined via SLS vs. mPEG_114_-*b*-PHHFarGE_5_ concentration *c*, recorded at *θ* = 90°.

Figure S35 shows the excess Rayleigh ratios *R*_vv_ determined via SLS for selected block copolymers of the mPEG_114_-*b*-P(HH)FarGE_m_ series.


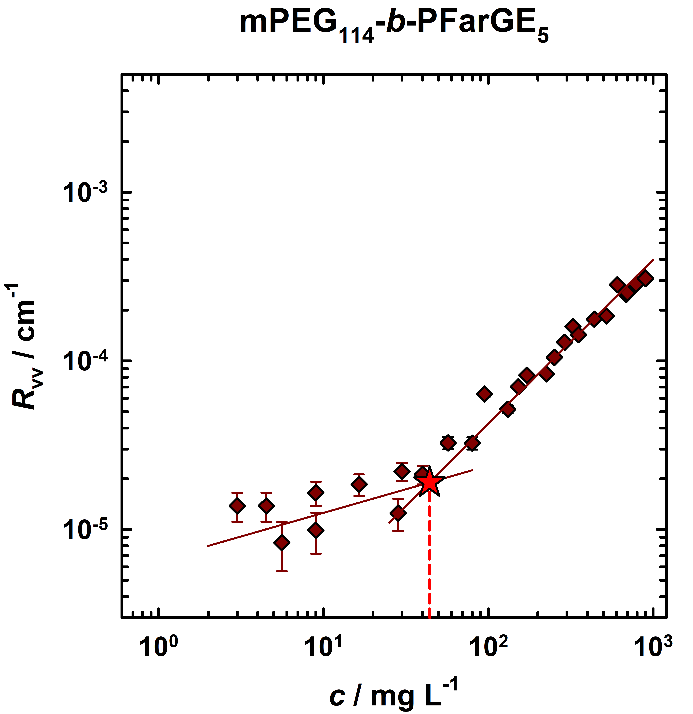

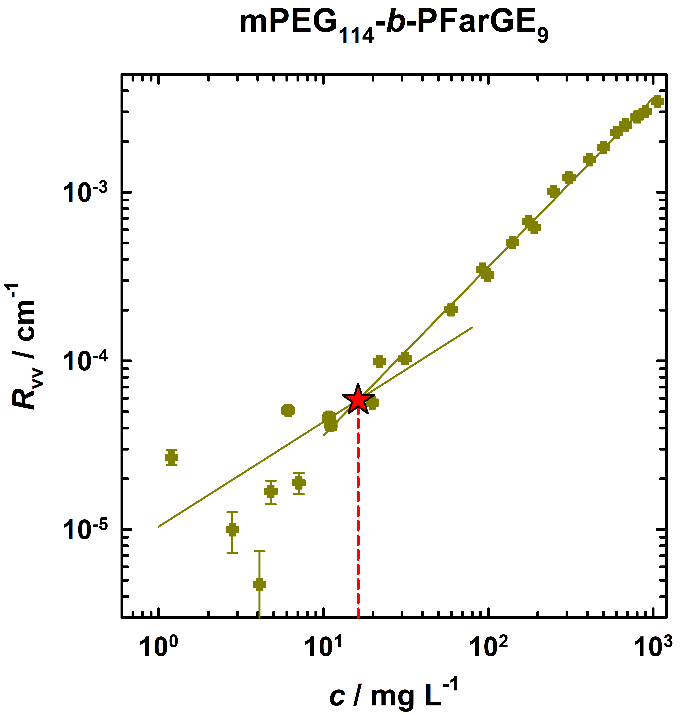

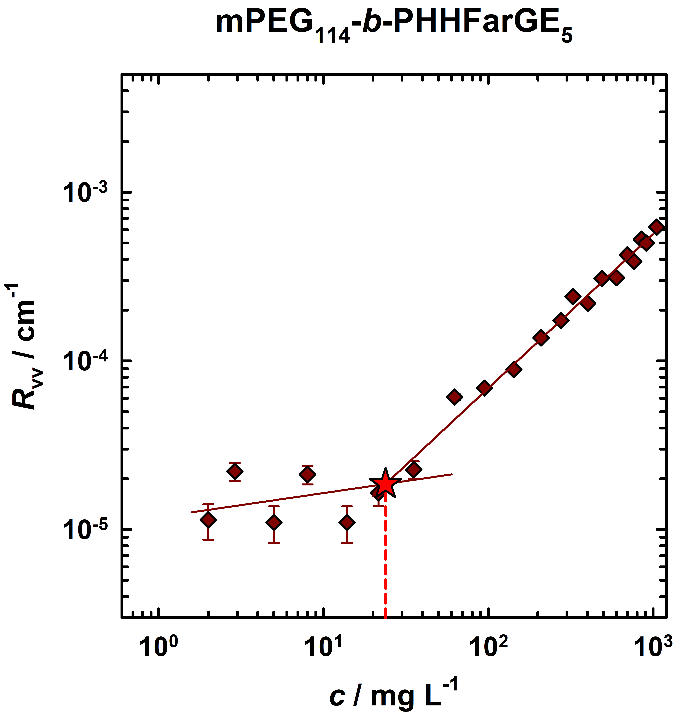

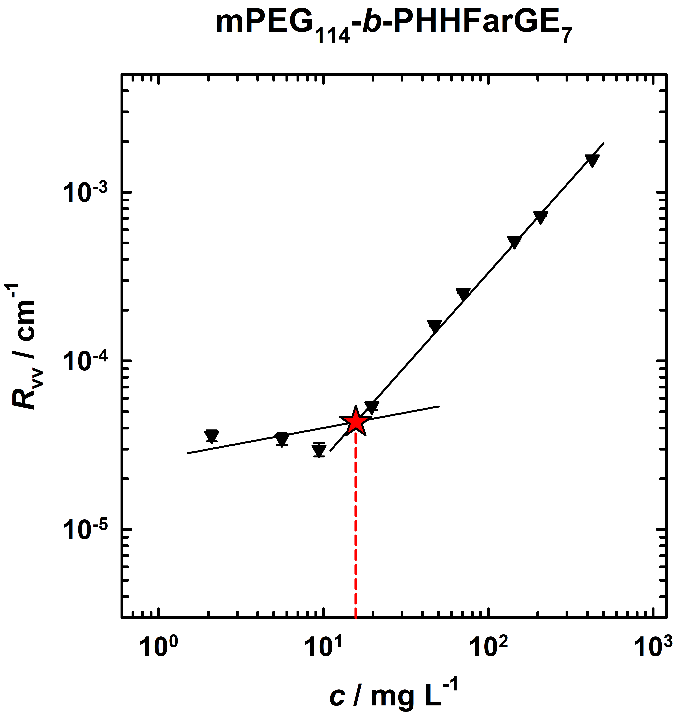


**Figure** S35: Plots of the excess Rayleigh ratio *R*_vv_ versus polymer concentration, allowing for determination of the CMCs via light scattering, shown for selected mPEG_114_-*b*-P(HH)FarGE_m_ diblock copolymers. CMCs are depicted with stars.

1. Phase Behavior of H_2_O/NaCl – *n*-decane – C_10_E_4_ / mPEG_114_-*b*-PFarGE_5_

Phase behavior for different conventional microemulsion systems of the type H_2_O/NaCl – *n*-decane – tetraethylene glycol monodecyl ether (C_10_E_4_) / mPEG_114_-*b*-PFarGE_5_, studied via *T*(γ) cuts at $\epsilon$ = 0.001 and $\phi$ = 0.5. The copolymer-to-surfactant ratio $\delta$ was varied. Details are outlined in the Experimental Section.


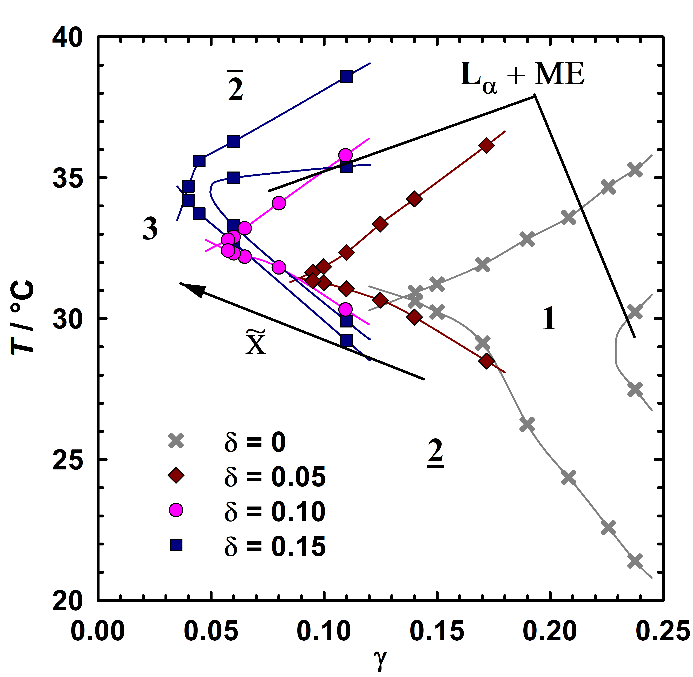


Figure S36: *T*(γ) diagrams of the system H_2_O/NaCl – *n*-decane – C_10_E_4_/mPEG_114_-*b*-PFarGE_5_, recorded with varying amounts of polymer in the amphiphile mixture (*δ*).

Systems with other mPEG_114_-*b*-PFarGE_m_ as well as those with mPEG_114_-*b*-PHHFarGE_m_ copolymers are shown in the main manuscript (Figures 4 and 5). The efficiency boosting effect of the mPEG_114_-*b*-PFarGE_m_ and mPEG_114_-*b*-PHHFarGE_m_ polymers is very similar, achieving solubilization efficiencies down to approximately $\tilde{\boldsymbol{\gamma}}$ $\boldsymbol{\approx}$ 0.09 with 5 wt.% of polymer in the amphiphile mixture (δ = 0.05).

1. References

(1) Le Zhao, C.; Winnik, M. A.; Riess, G.; Croucher, M. D. Fluorescence probe techniques used to study micelle formation in water-soluble block copolymers. *Langmuir* **1990**, *6*, 514–516.

(2) Wilhelm, M.; Le Zhao, C.; Wang, Y.; Xu, R.; Winnik, M. A.; Mura, J. L.; Riess, G.; Croucher, M. D. Poly(styrene-ethylene oxide) block copolymer micelle formation in water: a fluorescence probe study. *Macromolecules* **1991**, *24*, 1033–1040.

(3) Li, H.; Hu, D.; Liang, F.; Huang, X.; Zhu, Q. Influence factors on the critical micelle concentration determination using pyrene as a probe and a simple method of preparing samples. *Royal Society open science* **2020**, *7*, 192092.

(4) Kalyanasundaram, K.; Thomas, J. K. Environmental effects on vibronic band intensities in pyrene monomer fluorescence and their application in studies of micellar systems. *J. Am. Chem. Soc.* **1977**, *99*, 2039–2044.

(5) Aguiar, J.; Carpena, P.; Molina-Bolı́var, J. A.; Carnero Ruiz, C. On the determination of the critical micelle concentration by the pyrene 1:3 ratio method. *J. Colloid Interface Sci.* **2003**, *258*, 116–122.

(6) Hamai, S. Inclusion compounds in the systems of beta-cyclodextrin-alcohol-pyrene in aqueous solution. *J. Phys. Chem.* **1989**, *93*, 2074–2078.

(7) Wu, H. Correlations between the Rayleigh ratio and the wavelength for toluene and benzene. *Chemical Physics* **2010**, *367*, 44–47.

(8) Kaye, W.; McDaniel, J. B. Low-angle laser light scattering - rayleigh factors and depolarization ratios. *Applied optics* **1974**, *13*, 1934–1937.

(9) Teubner, M.; Strey, R. Origin of the scattering peak in microemulsions. *The Journal of Chemical Physics* **1987**, *87*, 3195–3200.

(10) Landau, L. D.; Lifshit︠s︡, E. M.; Pitaevskiĭ, L. P. *Statistical physics*; Pergamon international library of science, technology, engineering, and social studies 5, 9; Pergamon Press: Oxford, New York, 1980.

(11) Schelten, J.; Schmatz, W. Multiple-scattering treatment for small-angle scattering problems. *J Appl Crystallogr* **1980**, *13*, 385–390.

(12) Silas, J. A.; Kaler, E. W. Effect of multiple scattering on SANS spectra from bicontinuous microemulsions. *Journal of Colloid and Interface Science* **2003**, *257*, 291–298.

(13) Burauer, S.; Sachert, T.; Sottmann, T.; Strey, R. On microemulsion phase behavior and the monomeric solubility of surfactant. *Phys. Chem. Chem. Phys.* **1999**, *1*, 4299–4306.

(14) Schneider, K.; Verkoyen, P.; Krappel, M.; Gardiner, C.; Schweins, R.; Frey, H.; Sottmann, T. Efficiency Boosting of Surfactants with Poly(ethylene oxide)-Poly(alkyl glycidyl ether)s: A New Class of Amphiphilic Polymers. *Langmuir* **2020**, *36*, 9849–9866.

(15) Nishida, T.; Satoh, K.; Nagano, S.; Seki, T.; Tamura, M.; Li, Y.; Tomishige, K.; Kamigaito, M. Biobased Cycloolefin Polymers: Carvone-Derived Cyclic Conjugated Diene with Reactive exo-Methylene Group for Regioselective and Stereospecific Living Cationic Polymerization. *ACS Macro Lett.* **2020**, *9*, 1178–1183.

(16) Wang, A.; Wüstenberg, B.; Pfaltz, A. Enantio- and diastereoselective hydrogenation of farnesol and O-protected derivatives: stereocontrol by changing the C=C bond configuration. *Angew. Chem.* **2008**, *47*, 2298–2300.

(17) Mouzin, G.; Cousse, H.; Rieu, J.-P.; Duflos, A. A Convenient One-Step Synthesis of Glycidyl Ethers. *Synthesis* **1983**, 117–119.

(18) Schüttner, S.; Krappel, M.; Koziol, M.; Marquart, L.; Schneider, I.; Sottmann, T.; Frey, H. Anionic Ring Opening Copolymerisation of Terpenoid-derived Farnesyl Glycidyl Ether: A Facile Approach to Amphiphilic Polyethers. *Macromolecules* **2023**, *56*, 6928–6940.

(19) Hauenstein, O.; Reiter, M.; Agarwal, S.; Rieger, B.; Greiner, A. Bio-based polycarbonate from limonene oxide and CO 2 with high molecular weight, excellent thermal resistance, hardness and transparency. *Green Chem.* **2016**, *18*, 760–770.
